# Beyond Barriers, Big Crystallization Hurdles: Atropisomerism in Beyond Rule of Five Compounds Explored by Computational and NMR Studies

**DOI:** 10.1021/acs.molpharmaceut.5c00204

**Published:** 2025-04-27

**Authors:** Nikolaos Angelos Stamos, Benjamin Ries, Regina Schneider, Pavleta Tzvetkova, Florian Montel, Christian Jandl, Ulrike Werthmann

**Affiliations:** † CMC, DDS, Discovery Research, Boehringer Ingelheim Pharma GmbH & Co. KG, 88400 Biberach an der Riss, Germany; ‡ Computational Chemistry, Medicinal Chemistry, Discovery Research, 60325 Boehringer Ingelheim Pharma GmbH & Co. KG, 88400 Biberach an der Riss, Germany; § Analytical Development, Development, Boehringer Ingelheim Pharma GmbH & Co. KG, 88400 Biberach an der Riss, Germany; ∥ Open Innovation, Medicinal Chemistry, Discovery Research, Boehringer Ingelheim Pharma GmbH & Co. KG, 88400 Biberach an der Riss, Germany; ⊥ ELDICO Scientific AG, 4123 Allschwil, Switzerland

**Keywords:** bRo5 compounds, atropisomerism, crystallization, in silico calculations, NMR, rotational barriers

## Abstract

Stereochemical purity, stability, and selection of a suitable solid-state form are pivotal factors in pharmaceutical development, particularly for complex beyond Rule of 5 (bRo5) compounds. In this study, we explore the intricate interplay between atropisomerism and crystallization using two model bRo5 compounds, namely, ACBI1 and BI201335, both violating three of four Lipinski’s rules. One of the tool compounds exhibits Class 2 atropisomeric behavior, and the other is devoid of it. A diverse array of crystallization methods, including solution-phase crystallization, cocrystallization, and salt formation, were applied, revealing the critical role of atropisomerism-induced stereochemistry in polymorphism and nucleation outcomes. *In silico* torsion profile calculations and NMR studies were employed to elucidate the rotational energy barriers and confirm the presence or absence of atropisomerism. This comprehensive analysis highlights the significance of understanding stereochemical phenomena such as atropisomerism in designing and developing bRo5 compounds. By integrating advanced analytical techniques and crystallization strategies, this work provides novel insights into tailoring pharmaceutical properties for next-generation therapeutics.

## Graphical abstract

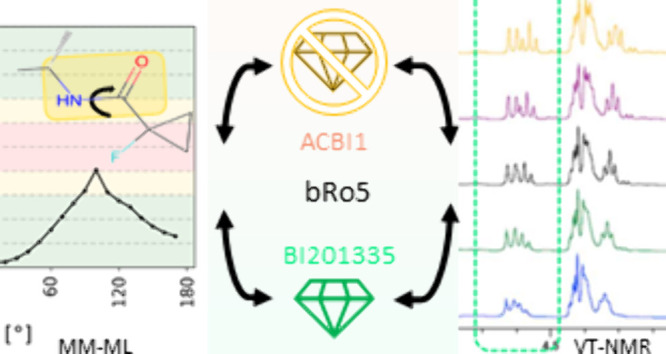

## Introduction

Crystallization is a foundational process in chemical processes where molecules organize into ordered three-dimensional arrays, forming crystalline solids. Usually, crystalline solid-state forms are strongly preferred against amorphous forms, as they avoid risks such as lack of stability or uncontrolled phase-conversions. Solution-phase crystallization typically begins with nucleation from a supersaturated solution, followed by crystal growth.^1^ In particular, heterogeneous nucleation is highly influenced by environmental factors, such as solvent composition, impurities, and contact surfaces. The resulting solid-state landscape of a molecule can be complicated by the existence of multiple crystalline forms, including salts, hydrates, solvates, cocrystals, and polymorphs.^2^ Traditional crystallization techniques, like solvent evaporation, exchange, and diffusion, can take weeks and require significant quantities of analyte.^3–6^

To assess solid-state forms of a compound and to select the best one for pharmaceutical development, knowledge of the crystal structures of the relevant solid-state forms is useful. However, crystallization of molecules, especially beyond Rule of 5 (bRo5) compounds, can be challenging. To address these limitations, innovative techniques such as Fujita’s “crystalline sponge” method have been developed, using a preformed porous host crystal to encapsulate and order small organic guest molecules.^7,8^ Additionally, electron diffraction has emerged as a useful technique for single crystal structure determination in a microcrystalline sample. However, it has limitations, including potential sample degradation from the electron beam and the dynamical effects should be considered.^9,10^ More recently, the Encapsulated Nanodroplet Crystallization (ENaCt) protocol^11,12^ has been developed to use microgram quantities of analyte for high-throughput crystallization. Inspired by protein crystallization techniques, ENaCt operates on the principle of concentration-driven nucleation and involves encapsulating nanoliter droplets of analyte solution in inert oil, allowing controlled solvent loss. Once the critical supersaturation point is reached, spontaneous crystallization occurs. This is achieved through a highly controlled and slow diffusion process, increasing the likelihood of forming more thermodynamically stable polymorphs. This protocol has proven effective in crystallizing various compounds, including *N*-heterocyclic carbenes,^13^ polyketides,^14^ a protease inhibitor,^15^ cannabidiol,^16^ and dihydropyridine APIs,^12^ and has not yet been tested on bRo5 compounds.

The Lipinski Rule of 5 (Ro5) was established as a guideline for predicting the oral bioavailability of drug candidates based on physicochemical properties (Table 1), such as molecular weight, lipophilicity, and hydrogen bonding capacity.^17^ When it comes to challenging drug targets with large binding sites,^18,19^ beyond the Rule of 5 (bRo5) compounds, such as the tool compound BI201335, have proven particularly useful. These compounds often exhibit higher conformational complexity, greater flexibility, and larger surface areas, which are advantageous in building up specificity and potency for targets with, for example, shallow binding pockets or for forming larger complexes, which include more than one target protein.^20^ This extended bRo5 profile includes proteolysis targeting chimeras (PROTACs), such as the tool compound ACBI1, which are designed to selectively target and degrade target proteins. PROTACs hold promise for treating diseases with unmet therapeutic needs^21,22^ but often suffer from unsatisfactory ADME profiles, with risks related to poor solubility,^23^ low permeability, and increased efflux and metabolism.^24^ While Lipinski’s^17^ Rule of 5 provides a framework for small molecule drug design, new chemical modalities falling outside this space necessitate additional profiling and screening filters,^25–33^ and the crystallization behavior of these larger and more flexible new chemical entities requires novel crystallization approaches as described.

**Table 1:** Lipinski’s Rule of 5 and Its Variations

variations of Lipinski’s Rule of 5			
variables	Ro5	extended Ro5 (eRo5)	beyond Ro5 (bRo5)
H-bond donors	*x* ≤ 5	*x* ≤ 5	*x* > 5
H-bond acceptors	*x* ≤ 10	*x* ≤ 10	*x* > 10
MW	*x* ≤ 500 Da	500–700 Da	*x* ≥ 700 Da
cLogP	0 ≤ *x* ≤ 5	0 ≤ *x* ≤ 7.5	*x* < 0 or *x* > 7.5

Many bRo5 molecules exhibit increased molecular weight, flexibility due to a high number of rotatable bonds, and conformational complexity, making them more likely to exhibit atropisomerism. These stereoisomeric forms arise from restricted rotation around chemical bonds.^19^ Atropisomerism, a specific form of axial chirality, was first observed nearly a century ago,^34^ with the term derived from the Greek *άτροπος*, meaning “without turn”.^35^ This type of chirality results from steric hindrance or electronic factors along a bond axis, resulting in conformers that do not readily interconvert. Atropisomers can be isolated as enantiomers or diastereoisomers, each with distinct properties including target binding and potency.^36^ A critical threshold for atropisomerism is a rotational energy barrier of around 20 kcal/mol, above which isomers do not readily interconvert.^19^ This threshold is particularly relevant in drug discovery and development because it helps determine whether a compound should be developed as a racemic mixture, as a single stereoisomer, or not developed given that biological systems are often chiral and can respond differently to each isomer. This was famously illustrated in the case of thalidomide,^37,38^ where stereoisomers exhibited differing biological effects.

Atropisomeric scaffolds frequently used in drug discovery include biaryls, heterobiaryls, benzamides, diarylamines, and anilides.^39–42^ LaPlante’s classification further categorizes atropisomers based on their half-life of racemization at 37 °C:^18,43^ Class 1 (*t*_1/2_ < 60 s, Δ*G* < 20 kcal/mol) atropisomers, which are generally treated as achiral; Class 2 (60 s < *t*_1/2_< 4.5 years, 20 kcal/mol < Δ*G* < 30 kcal/mol) atropisomers, which have intermediate stability creating regulatory challenges due to their tendency to racemize on time scales from minutes to months, earning them the nickname “lurking menace”;^44^ and Class 3 (*t*_1/2_ > 4.5 years, Δ*G* > 30 kcal/mol) atropisomers, which are stable enough to be developed as enantiomers or diastereomers. Examples of FDA-approved Class 3 atropisomers include telenzepine,^45^ colchicine,^46^ lesinurad,^47^ and sotorasib.^48^

Advanced techniques are essential for detecting and analyzing atropisomerism. Solid-state analyses, such as X-ray crystallography (sc-XRD) and electron diffraction (ED), allow for the direct observation of atropisomerism in crystalline structures. *In silico* calculations, including molecular mechanics (MM) supported by machine learning (ML) or quantum mechanics (QM) density functional theory (DFT), are employed to predict rotational barriers and identify stable atropisomers. Variable-temperature nuclear magnetic resonance (VT-NMR) further allows assessment of the energy barriers of atropisomeric rotations, while 2D NMR provides additional structural insights. The two tool compounds in this study, ACBI1 and BI201335, both violate three Ro5 thresholds, namely, molecular weight, lipophilicity, and hydrogen bond acceptors. The first goal was to obtain crystalline forms of both compounds. BI201335 crystallized readily using conventional methods, while ACBI1 exhibited significant resistance to crystallization. A possible explanation lies in the phenomenon of atropisomerism and specifically Class 2 atropisomers, which are particularly challenging in drug discovery due to their intermediate stability. Furthermore, the second target aimed to investigate whether atropisomerism was a key factor influencing the behavior of ACBI1 compared to BI201335. Conventional and nonconventional crystallizations were applied on ACBI1 to address this hypothesis.

*In silico* approaches can be used to predict the conformational behavior of molecules to understand, for example, the flexibility of molecules. Usually, such approaches need to balance accuracy versus calculation speed.^49,50^ Methods on the MM level using generalized AMBER force field^51^ or OpenFF 2.0 ^52,53^ are usually comparably efficient in terms of computation time and hardware demand. In many use cases, they give reasonably accurate insights into molecular properties.^50,54^ Nevertheless, in comparison to the aforementioned methods, the QM level calculations offer a significantly more accurate property estimation at the cost of a significantly increased time and hardware demand cost.^55–57^

A modern compromise between both theory levels is represented by MM-ML methods like ANI-1,^58^ Espaloma,^59^ AIMNet,^60^ or MACE-OFF23 ^61^ that improve the model accuracy and do not increase the calculation costs significantly. Such methods can be nicely employed to efficiently screen a molecule and find degrees of freedom, which would then be investigated more deeply on a QM level.^50,61^ However, these calculations are carried out in vacuum and only serve as predictions of the torsional profile; therefore, we employed NMR studies in order to gain deeper insights into the rotational barriers.

In the case of complex molecules, like bRo5, it is often observed that these molecules show multiple (usually doubled) sets of signals in their NMR spectra at room temperature. This observation could be due to restricted single bond rotations or the presence of diastereoisomers. By performing variable temperature NMR experiments, it is possible to follow the signal movement. If the rotational barrier is overcome, the signals combine into one single signal. The stepwise increase of temperature is continued until the so-called coalescence point is reached, where the previously two separate signals merge into a single broad signal. This means that on the NMR time scale the rotation around this single bond is equivalent to the lifetime of each single state of the rotamer. To discriminate whether the multiple set of signals can be attributed to diastereoisomers or rotamers, proton 2D ROESY experiments are useful. In the case of rotamers, an exchange of signals would be observed. At the coalescence temperature, it is possible to calculate the energy barrier of this single bond rotation using the modified Eyring equation.^62,63^

This equation allows quick evaluation of the rotational energy barrier as follows:

$$\Delta G^{‡}=RT_{\mathrm{coal}}\left[22.96+\ln\left(\frac{T_{\mathrm{coal}}}{\delta \nu }\right)\right]$$

where *R* is the universal gas constant [J/mol·K], *T*_coal_ is the coalescence temperature [K], and δν is the difference of the Larmor frequencies in [Hz] for the two observed signals for the rotameric pair. The energy calculated using this equation is given in J/mol, which is then converted into kcal/mol.

In this study, we have used the combination of crystallization approaches, MM-ML and DFT calculations, and NMR studies to address the challenges in crystallizing the two example molecules, ACBI1 and BI201335, and clarify the possible reasons behind them.

In summary, the crystallization behavior and stereochemical stability of bRo5 molecules like PROTAC compounds^64^ underline the importance of understanding atropisomerism in drug research and development. These stereochemical phenomena not only affect the solubility, permeability, metabolism, pharmacokinetic behaviors, and stability of compounds but also influence their efficacy, selectivity, and overall therapeutic potential. This study explores the solid-state and stereochemical behavior of two model bRo5 compounds (ACBI1 and BI201335), which violate three of Lipinski’s Rule of 5, the H-bond acceptors, molecular weight, and lipophilicity, examining their properties experimentally through various crystallization techniques, computationally through calculations on the MM-ML and QM DFT level, and analytically via VT-NMR and ROESY to predict and analyze their behavior.

## Materials and Methods

### Chemicals

ACBI1 and BI201335, the two bRo5 tool compounds, were provided by opnMe.com and were not further purified (Scheme 1). All reagents and solvents were purchased from commercial suppliers and used without further purification unless otherwise specified, and stock solutions of 0.1 M were prepared with the appropriate solvents.

**Scheme 1:**
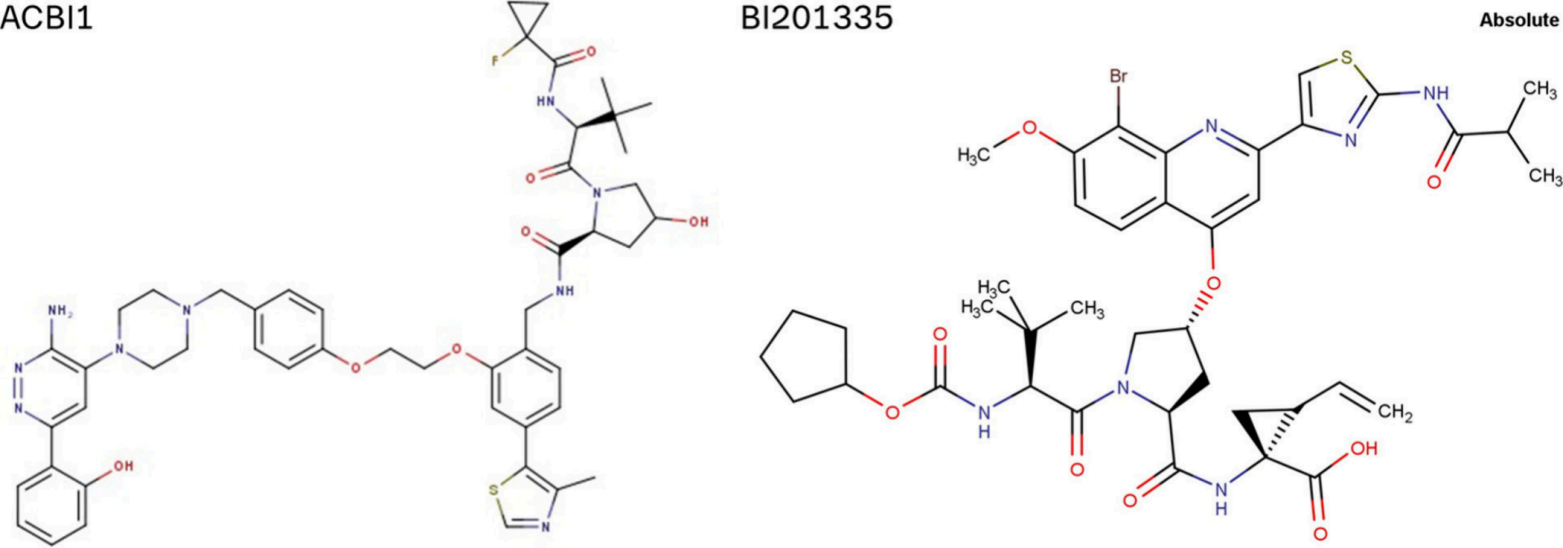
Chemical Structures of the under-Investigation bRo5 Tool Compounds ACBI1 and BI201335

### Instrumentation

#### Powder X-ray Diffraction (p-XRD)

The samples were analyzed using a STOE Stadi P X-ray powder diffractometer in transmission mode equipped with a position sensitive detector (STOE, Darmstadt, Germany) using CuK_α1_ radiation with λ = 1.5406 Å and a divergence slit of 1°. The X-ray tube was run with a power of 40 kV and 40 mA. The samples were prepared on flat sample holders between two acetate foils and were rotated during the measurement. Diagrams were recorded in a 2θ range between 3° and 40°.

#### Single Crystal X-ray Diffraction (sc-XRD)

A .cif file of the low-resolution sc-XRD data for ethanol-solvated BI201335 is included in the Supporting Information. A colorless birefringent prismatic crystal of the dimensions 0.20 × 0.15 × 0.10 mm^3^ was mounted in a nylon loop. All measurements were made on an AFC11 diffractometer equipped with a CuKα Rigaku Micromax 007 using CuKa radiation (λ=1.542 Å) and a Pilatus 200k detector. Integration of intensities and refinement of cell parameters was accomplished using CrysalisPro.^65^ Observation of the crystal after data collection showed no signs of decomposition. Using Olex2,^66^ the structure was solved with ShelXT using intrinsic phasing methods and refined with ShelXL using least-squares minimization (Table 3).^67–70^

#### Electron Diffraction (ED)

The sample of BI201335 was finely dispersed on a standard TEM grid (amorphous carbon on Cu) without grinding and measured on an ELDICO *ED-1* electron diffractometer at room temperature using the software ELDIX.^71^ The device is equipped with a LaB_6_ source operating at an acceleration voltage of 160 kV (λ = 0.02851 Å) and a hybrid-pixel detector (Dectris QUADRO). Suitable crystals were identified in the STEM (scanning transmission electron microscopy) imaging mode, and diffraction was recorded in continuous rotation mode with a beam diameter of ca. 750 nm. A total of 17 particles were measured and 9 were used for structure solution and refinement. Parts of measurements showing significant beam damage or shadowing by the grid were omitted. Further data collection details are given in Table 2.

**Table 2:** ED Data Collection Details of the Crystals of BI201335 Used in Refinement

crystal no.	angular range [°]	rotation per frame [°]	exposure time [s]	total exposure [s]	frames measured	frames used
1	–30 to +30	0.5	0.5	60	120	1–120
2	–45 to +55	0.5	0.5	100	200	21–100
3	0 to +60	0.5	0.5	60	120	1–50
4	–30 to +20	0.5	0.5	50	100	1–55
5	–50 to +40	0.5	0.5	90	180	1–80
6	0 to +60	0.5	0.5	60	120	1–70
7	–60 to +20	0.5	0.5	80	160	1–140
8	–60 to +20	0.5	0.5	80	160	1–120
9	–60 to +20	0.5	0.5	80	160	1–90

Data were processed and evaluated using the APEX4 software package.^72^ After unit cell determination, the frames were integrated separately for each crystal, then merged, scaled, and corrected for Lorentz effects, scan speed, background, and absorption using SAINT and SADABS.^73,74^ Space group determination was based on systematic absences, E statistics, and successful refinement of the structure. The structure was solved using ShelXT and refined with ShelXL in conjunction with ShelXle.^68–70^ Least squares refinements were carried out within the kinematic approximation by minimizing Σ*w*(*F*_obs_^2^ – *F*_calc_^2^)^2^ with the ShelXL weighting scheme and using neutral electron scattering factors.^69,75^ H atoms were placed in calculated positions based on typical distances for neutron diffraction and refined as rigid rotating groups with *U*_iso_(H) = 1.5·*U*_eq_(*C*) for methyl and using a standard riding model with *U*_iso_(H) = 1.5·*U*_eq_(C/N) for other groups. H atom positions on heteroatoms were refined freely if possible and otherwise with a riding model or just a distance restraint, as data quality allowed. Similarity and rigid bond restraints were used to ensure convergence within the physically meaningful limits. The unit cell between the two data acquisitions indicated a minute difference, although the low resolution sc-XRD was an ethanol solvate, and the same crystals were used to perform ED, and the structure was unsolvated, most probably due to the high vacuum (Table 3).

**Table 3:** Structure and Refinement Details for BI201335

CCDC number	2421398	Supporting Information
polymorphic form	unsolvated form I	ethanol solvated form II
method	electron diffraction	low resolution sc-XRD
empirical formula	C_40_H_49_BrN_6_O_9_S	C_40_H_49_BrN_6_O_9_S + EtOH
formula weight	869.81	869.81
temperature [K]	298	100
crystal system	monoclinic	monoclinic
space group	*P*2_1_	*P*2_1_
*a*, *b*, *c* [Å]	6.35(5), 18.41(15), 18.55(16)	6.5326(13), 17.876(4), 18.875(4)
α, β, γ [°]	90, 91.53(4), 90	90, 94.55(3), 90
volume [Å^3^]	2168	2197.2(8)
*Z*	2	2
number of reflections	16132	12204
number of independent reflections	5076	7468
completeness	0.832	N/A
data/restraints/parameters	5076/831/530	N/A
goodness of fit	1.06	1.043
final *R* factors [*I* > 2σ(*I*)]	*R*_1_ = 0.1616, *wR*_2_ = 0.3791	*R*_int_ = 0.1754
final *R* factors [all data]	*R*_1_ = 0.1875, *wR*_2_ = 0.4025	N/A

CCDC deposition number (2421398) contains the supplementary crystallographic data for unsolvated BI201335. The data can be obtained free of charge from The Cambridge Crystallographic Data Centre via www.ccdc.cam.ac.uk/structures/.

#### ThermoGravimetric Analysis (TGA)

Thermogravimetric analysis thermograms were recorded on a TA TGA5500 Discovery Series system at a heating rate of 10 K/min in open γ-Al_2_O_3_ crucibles under a dry nitrogen atmosphere, tethered with a Thermo Scientific Nicolet iS10 FT-IR analyzer in the range of 4000–400 cm^–1^.

#### Differential Scanning Calorimetry (DSC)

Differential scanning calorimetry thermograms were recorded on a TA DSC2500 Discovery Series system at a heating rate of 10 K/min in open Al-pans under a dry nitrogen atmosphere. Typical sample weights were 5–10 mg.

#### Dynamical Vapor Sorption (DVS)

Dynamical vapor sorption data were recorded on a DVS intrinsic system from surface measurement systems (SMS) under a N_2_ atmosphere. Water sorption/desorption isotherms were recorded from 0% to 90% RH followed by an additional run from 90% to 0% RH in equal steps of 10% RH.

#### Polarized Light Microscopy (PLM)

Photographs of each crystalline sample were recorded on a Zeiss AXIO Imager.A2m equipped with polarized light filters and an Axiocam 705 color system.

#### Nuclear Magnetic Resonance (NMR)

^1^H NMR spectra were recorded in a suitable deuterated solvent using a Bruker Avance IIIHD NMR spectrometer with a working base frequency of 400 MHz, equipped with a helium cryogenically cooled ^1^H/^13^C /^15^N TCI probehead. The temperature limits of the probehead are from −40 °C up to 150 °C. The ^1^H NMR experiments are recorded at a proton resonance frequency of 400.13 MHz, and 100.61 MHz was used for the ^13^C frequency. The temperature is regulated using a Bruker Smart Variable temperature unit and calibrated using NMR-thermometer sample calibration. The assignment of the proton and carbon signals is performed using ^1^H and ^13^C NMR experiments, such as 1D ^1^H, 1D ^13^C, and 2D experiments: ^1^H–^1^H COSY, ^1^H–^1^H ROESY, ^1^H–^13^C HSQC, and ^1^H–^13^C HMBC. The chemical shifts (δ_H_ and δ_C_) are reported in parts per million referenced to the 0.01% of nondeuterated signal of the deuterated solvent in the proton-based experiments and to the signal of the fully deuterated solvent signal in the carbon dimensions.

## Experimental Methods

For the polymorphic and salt and cocrystal screening of the two compounds, at the outset approximate solvent solubility (ASS) test was performed for the identification of solvents, antisolvents, and nonsolvents. The experimental procedures that were followed in both polymorphic and salt and cocrystal screening were the same. Initially, the samples from the solvent solubility test were thermally cycled (TC) and placed for slow solvent evaporation (SE). Since TC and SE require a longer time frame, this method potentially favors more stable forms. If there were no suitable crystals, then thermal cycling and antisolvent addition (ASA) was performed, where the ASA would result in more metastable forms since it is in shorter time scale. To determine the wider landscape of crystal forms, solvent drop grinding (SDG)/liquid assisted grinding (LAG) and sublimation were incorporated in the experimental procedures to explore the high-energy crystallization methods, which are performed in shorter timelines favoring more metastable forms. In contrast, vapor diffusion (VD) and encapsulated nanodroplet crystallization (ENaCt) are long-term crystallization methods that favor more stable forms (Figure 1). The extensive polymorphic screen that was currently described would provide the polymorphic landscape of the compounds, and if this was unsuccessful, the efforts were redirected to a salt and cocrystal screening. The main goal of this comprehensive crystallization screening for both compounds was to obtain at least one crystalline form; when this was achieved, the efforts would be focused to the next compound.

**Figure 1:**
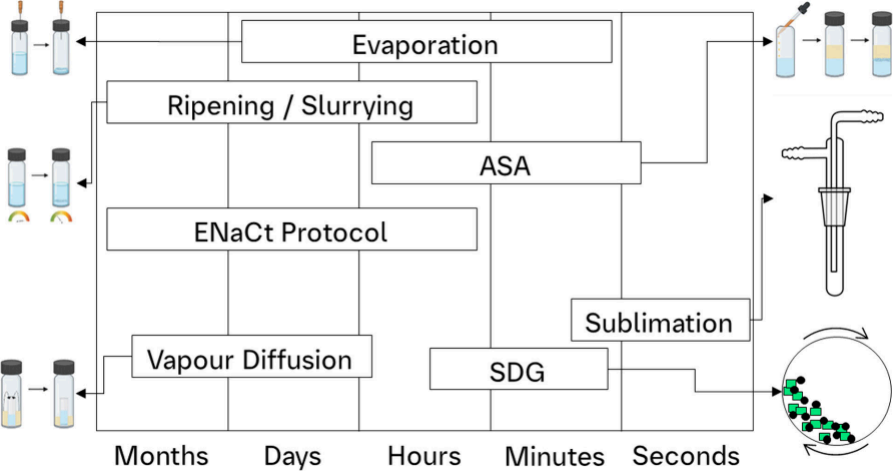
Crystallization experiments demonstrating the time scales that were employed to favor stable polymorphic forms at longer timeframes or metastable polymorphs at shorter timeframes.

### General Characterization

Postexperiment, the samples were analyzed using p-XRD to assess crystallinity. Only samples exhibiting crystalline material were subjected to further characterization, such as microscopy, TGA, DSC, and DVS.

### Approximate Solvent Solubility for Both Compounds

The approximate solubility of both compounds was determined in various organic solvents and solvent systems with a range of properties, including protic, aprotic, polar, and nonpolar solvents. Solubility tests were conducted under stirring at 40 °C, with concentrations ranging from 400 to 5 mg/mL. Solvents in which the solubility was below 5 mg/mL were classified as antisolvents.

### Polymorph Screening

#### Thermal Cycling with Slurrying/Ripening and Slow Solvent Evaporation for Both Compounds

Thermal cycling experiments were performed using the solutions prepared during the approximate solvent solubility screening in closed vials. The thermal cycling process involved holding samples at room temperature for 2 h, ramping up to 40 °C at a rate of 0.1 °C/min, holding at 40 °C for 2 h, and cooling back to room temperature at the same rate. This cycle was repeated continuously for 72 h to allow for gradual nucleation and crystal growth. Following thermal cycling, vials were pierced with a needle and left in a fume hood to facilitate complete solvent evaporation over an additional 7–14 days.

#### Seeding for BI201335

To obtain higher-quality crystals, seeding experiments were conducted according to the approximate solvent solubility, using isopropyl acetate (iPAc), acting as an antisolvent, and ethanol as the solvent system. A 15% w/w solution of BI201335 in iPAc was heated to 70 °C, and ethanol was added to achieve a 92:8 v/v iPAc/EtOH ratio. The solution was seeded with the crystals obtained from thermal cycling and slow evaporation at 70 °C, cooled to 65 °C, and held for 1 h to establish a seed bed.

The seeded slurry was then cooled at a controlled rate of 5 °C/h until it reached 25 °C, after which it was stirred at ambient temperature for at least 4 h. The crystalline ethanol solvate was isolated by vacuum filtration, and the filter cake was washed with ethanol. The resulting solvated crystals of BI201335 were dried under vacuum at 80 °C with a nitrogen purge for approximately 8 h. This procedure successfully produced higher-quality crystalline material compared to the initial thermal cycling experiments, confirming the utility of seeding for improving the crystal quality.

#### Thermal Cycling with Slurrying/Ripening and Anti-Solvent Addition for ACBI1

Thermal cycling was conducted on ACBI1 using 0.2 mL of each of the 25 solvents identified during the solubility screening. Samples that formed slurries underwent thermal cycling for 72 h, since this approach often yielded more stable polymorphic forms. Conversely, solvents that bore clear solutions were brought to 40 °C, and antisolvent was added dropwise until turbidity was observed. These suspensions were then subjected to an additional 72 h of thermal cycling, which often yielded more metastable polymorphic forms. Three outcomes were observed: slurries formed with solvent, slurries formed with both solvent and antisolvent, and clear solutions that remained post-thermal cycling, which were slowly evaporated.

For the first two cases, centrifugation was performed to isolate the solids, which were subsequently analyzed via p-XRD. The supernatant liquids were retained and allowed to evaporate slowly for further analysis. For the third case, where clear solutions persisted post-thermal cycling, slow solvent evaporation was initiated to attempt crystallization.

#### Solvent Drop Grinding or Liquid Assisted Grinding for ACBI1

For solvent-drop grinding, a high-throughput mechanochemical method, multiple samples of approximately 20 mg each were prepared using 40 solvents with varying properties: protic, aprotic, polar, and nonpolar. Each sample was “wetted” with 20 μL of the respective solvent, a minimal amount sufficient to coat the compound without dissolving it. Three stainless steel ball bearings were added to each vial, which was then sealed. The samples were subjected to vigorous milling at 4500 rpm at ambient conditions in an appropriate milling apparatus for 1 h. The mechanochemical energy imparted by the grinding process can disrupt the molecular arrangements, facilitating the nucleation of potentially metastable crystalline forms. Postgrinding, the samples were analyzed via p-XRD to determine if crystallinity had been achieved.

#### Sublimation for ACBI1

Sublimation was conducted at reduced pressure. First, the thermal properties of the compound were studied by using differential scanning calorimetry (DSC) to determine the melting point. Based on DSC thermograms, the compound’s melting point was determined to be approximately 170 °C. The sample was then placed in a test tube fitted with a sublimation apparatus and subjected to high vacuum conditions (∼10^–6^ mbar). Under reduced pressure, the melting point decreases, enabling sublimation to occur at lower temperatures. The system was gradually heated in 10 °C increments while ensuring even warming of the sample. Near the melting point, additional energy was applied to drive the transition from solid to gas, allowing the compound to deposit as a solid on the coldfinger of the apparatus. This technique relies on rapid energy transfer to promote metastable crystallization, although in harsh conditions for organic compounds.

#### Encapsulated Nanodroplet Crystallization (ENaCt) Protocol for ACBI1

The ENaCt protocol was employed to explore the potential for stable polymorphic forms of ACBI1. The experimental setup utilized 96-well plates, where nanoliter droplets of active pharmaceutical ingredient (API) solutions were deposited into oil droplets. While the oil served as a diffusion barrier, the solvent slowly diffused into the oil over time, allowing the concentration of the solution to increase gradually over the course of 60 days. Once the critical supersaturation point was reached, crystallization occurred.

This slow diffusion process allows for the exploration of a wide array of variables, including the concentration of the solution, the type of solvent, the type of oil, and the ratio of oil to solvent with the API. Eight suitable solvents were selected based on the solubility screen, and five different concentrations were prepared for each. Six oil systems were used, with two API/oil ratios incorporated into the design and duplicated. The initial samples were manually prepared, but the subsequent experimental process was fully automated preparation with the SPT Labtech Mosquito system, and daily imaging with the Formulatrix Rock Imager 1000 ensured precise monitoring of crystallization progress using polarized and nonpolarized microscopy for image capture. Successful experiments were repeated in 6- and 12-fold replicates at two different API/oil ratios.

#### Salt and Cocrystal Screening of ACBI1

To initiate the salt and cocrystal screening of ACBI1, the p*K*_a_ values of the molecule were identified computationally (piperazine p*K*_a_ = 7) with MoKa software, indicating potential compatibility with strong acids, with a difference of 2 orders of magnitude. Different acids were selected for the initial experiments. These acids were chosen for their diversity in molecular weight and physicochemical properties, ensuring a broad screening range. Stock solutions of the acids were prepared at a concentration of 0.1 M, and a slight excess of acid (1.05 equiv) was added to ensure the reaction proceeded efficiently.

Multiple solvent systems, selected based on the earlier approximate solubility screening, were used in the experiments. These solvents represented a range of polarities and protic/aprotic properties to capture the effects of different environments on salt formation. The experimental procedure involved adding the acid stock solution to the vials and allowing the solvent to evaporate. Simultaneously, stock solutions of the API were prepared at a concentration of 100 mg/mL in the solvent systems, and 200 μL of these solutions was added to the respective vials, resulting to 20 mg per vial. A magnetic stir bar was included in each vial to ensure thorough mixing during the subsequent thermal cycling process, which followed the established thermal cycling protocol.

Initial observations were recorded, and after thermal cycling the samples that produced solids or slurries were centrifuged, and the resulting solids were analyzed using p-XRD. For samples that remained as clear solutions post-thermal cycling, antisolvent addition experiments were initiated. Antisolvents identified during the earlier polymorphic screening were used, and the clear solutions were maintained at 40 °C during the dropwise addition. When turbidity was observed, the antisolvent addition was stopped, and the samples underwent another 72 h thermal cycling protocol. After this secondary thermal cycling, any solids or slurries formed were analyzed via p-XRD, while clear solutions were left to evaporate slowly.

#### Primary Salt Screen of ACBI1

The primary salt screening began with six strong acids chosen for their diversity in molecular weight, p*K*_a_ values (−6 to 2), and physicochemical properties, including hydrochloric acid, sulfuric acid, and naphthalenedisulfonic acid. Stock solutions of the acids (0.1 M) were combined with the API (1:1.05 molar ratio) using four solvent systems, namely, 2-propanol (IPA), acetone, ethyl acetate (EtOAc), and tetrahydrofuran (THF), selected based on solubility and polarity. Thermal cycling was performed, and following thermal cycling, the samples that formed solids or slurries were centrifuged and analyzed via p-XRD.

Clear solutions from thermal cycling were subjected to antisolvent addition (*tert*-butyl methyl ether, ^t^BME), followed by another 72 h of thermal cycling. Resulting solids or slurries were analyzed by p-XRD, while clear solutions were left to evaporate slowly. However, all samples analyzed via p-XRD showed amorphous material, highlighting the challenges of salt formation for ACBI1 under these conditions.

#### Extended Salt and Cocrystal Screening of ACBI1

Encouraged by the partial success of the naphthalenedisulfonic acid/ethyl acetate combination, the study progressed to an extended salt and cocrystal screening. This phase utilized 24 acids, including naphthalenedisulfonic acid, ethanedisulfonic acid, cyclamic acid, and oxalic acid, chosen based on their p*K*_a_ compatibility with the API. For the cocrystal screening, weaker acids (coformers) were selected to enhance potential favorable interactions with the API.

The experimental setup followed the established primary salt screening procedure, utilizing the same four solvent systems (IPA, acetone, EtOAc, and THF). Thermal cycling, isolation of solids, antisolvent addition, and secondary thermal cycling were conducted.

#### Solvent Drop Grinding/Liquid Assisted Griding Salt Formation for ACBI1

Solvent drop grinding experiments were performed on the successful salt or cocrystals to explore the polymorphic form landscape and identify new forms or to obtain higher crystallinity on the existing form. As described, since this is a high-energy mechanochemistry experiment, it favors more finding metastable forms. Promising salts, including napadisylate, edisylate, and cyclamate, were subjected to solvent-drop grinding in different solvent systems to improve crystallinity and explore potential new polymorphic forms.

#### Vapor Diffusion Salt Formation for ACBI1

The principle of vapor diffusion involves using a suitable solvent to dissolve the API and the acid, creating a saturated solution. This solution was transferred into small vials, each capped with a pierced lid to allow vapor exchange. These vials were then placed inside larger vials containing a more volatile antisolvent. The antisolvent vapors gradually diffused into the solution-containing vials, creating a concentration gradient. As the antisolvent mixed with the solvent, the system reached supersaturation, triggering spontaneous crystallization.

To target more stable crystalline forms, vapor diffusion experiments were conducted. Two high-boiling solvents, DMSO and NMP, were used to dissolve the API and acids (cyclamic and naphthalenedisulfonic acid). Six antisolvents (methanol, diethyl ether, acetone, ethyl acetate, acetonitrile, and THF) were selected based on miscibility with the primary solvents and their higher vapor pressure compared to the solvents in the inner vial. Two API-to-acid ratios (1:1 and 1:2) were tested.

### Computational Torsion Profile Prediction

#### Fast Torsion Profile Screening with MM-ML

The torsion screening approach was performed on the MM-ML level using our tool, which can be retrieved from GitHub. First, all rotatable bonds of interest were automatically detected in the molecule of interest. Next the molecule was automatically fragmented around each bond into smaller fragments using the openff-fragmenter based on the algorithm by Stern et al.^76^ Using the RDKit ETKDG-Conformation Generator,^77^ the fragments were placed into a 3D setting. Afterward the initial torsion profile coordinates were generated using RDKit.^78^ The target torsion of a fragment was rotated in 10° steps to retrieve an initial torsion profile, yielding 36 dihedral angle positions. After each step, a fast MMFF94 ^79^ optimization was performed with position restraints and a force constant k of 10^5^ kJ/mol applied to the target bond. Next the initial profile was optimized in the MM-ML approach using the OpenForceField-MACE^61^ model together with openMM-ML (github.com/openmm/openmm-mL). During the optimization of each torsion angle coordinate set, position restraints with a k of 10^6^ kJ/*mol* were applied. The relative energies were generated by subtracting from all potential energies the minimal potential energy, yielding the torsion profile of a bond.

#### Accurate Torsion Profile Calculation with QM

After the screening approach, a set of QM calculations on the DFT level using the wB97XD functional^80^ and cc-pVDZ basis set in Gaussian^81–83^ was used together with larger fragments of the molecule only on the torsions of interest. The described workflow was implemented in TorsionProfiler an Open-Source package, which can be retrieved from GitHub at https://github.com/Boehringer-Ingelheim/TorsionProfiler.

## Results

The crystallization experiments followed a systematic approach (Figure 2), starting with an evaluation of the molecular structure to identify features that could influence the crystallization behavior. Functional groups, rotatable bonds, and overall molecular flexibility were assessed, as highly flexible molecules are prone to oiling out during crystallization attempts. This initial analysis informed the subsequent experimental design, particularly for polymorphic form screening.

**Figure 2:**
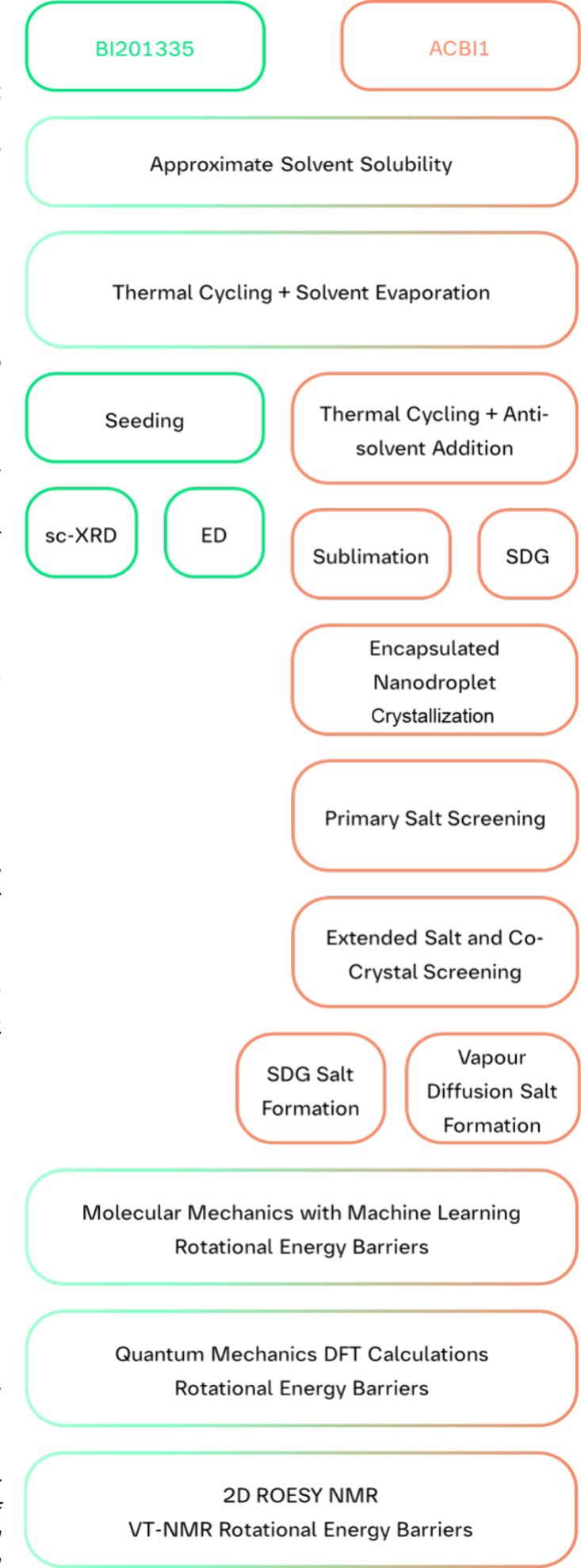
Process diagram (top to bottom) of the investigation for BI201335 (green) and ACBI1 (orange). Initially, assessment of solubility in the respective solvents and TC with SE was performed with both compounds. The subsequent analytical and experimental methods were adapted according to the outcomes of the initial results. Rotational barrier differences were identified by *in silico* and analytical methods.

### Approximate Solvent Solubility for Both Compounds

The approximate solvent solubility tests for both compounds provided essential insights into their solvent compatibility and formed the foundation for subsequent crystallization experiments.

ACBI1 was tested in 25 solvents with varying properties. ACBI1 was soluble in the concentration range between 50 and 400 mg/mL in most organic solvents, with lower solubility (<5 mg/mL) observed in a few, which were classified as potential antisolvents. The compound was confirmed to be hydrophobic (Table S1).

Similarly, BI201335 exhibited good solubility in the concentration range of 75–400 mg/mL in 10 solvents, and solvents with lower solubility of the compound were categorized as antisolvents (Table S53). Both compounds demonstrated similar solubility profiles, guiding the next steps in the crystallization process, which included thermal cycling with slurrying/ripening and slow solvent evaporation.

### Polymorph Screening

#### Thermal Cycling with Slurrying/Ripening and Slow Solvent Evaporation for Both Compounds

For both molecules, thermal cycling and solvent evaporation experiments were conducted by using the identified solvents. In all conditions that were used with ACBI1 samples, amorphous material was yielded, which was confirmed by powder X-ray diffraction (p-XRD) (Table S2).

The thermal cycling experiments for BI201335 were followed by slow evaporation, producing amorphous material in most cases (Table S54). Notably, the ethanol sample yielded form I crystalline material, although the crystals were too small for structural elucidation and reserved for subsequent seeding experiments, which resulted in suitable crystals for structural elucidation with sc-XRD and ED.

#### sc-XRD and ED for BI201335

BI201335 crystals obtained from the seeding experiments were analyzed using single crystal X-ray diffraction (sc-XRD) with in-house capabilities. This analysis provided a low-resolution structure of the ethanol solvate (Figure 3 left). To achieve better structural elucidation, electron diffraction (ED) was performed, yielding significantly improved resolution for the solvent-free crystal structure (Figure 3 right). The most notable feature of both structures is the double hydrogen bond between the carboxylic acid and the amide-substituted thiazole, which gives the structure more rigidity and thus greatly facilitates crystallization. An overlay of both structures shows a similar geometry (Figure 4). The part of substituted aromatics is almost identical (bottom in Figure 4), but closer to the ethanol position, the conformation deviates significantly more as the solvent-free structure has freedom to adjust to the free space (top in Figure 4). However, it is also clear that the molecule does not fully rearrange to fill the space and therefore the structure features voids upon solvent removal and potentially is the reason that DVS isotherm plot indicated slight hygroscopicity (Figure S8).

**Figure 3:**
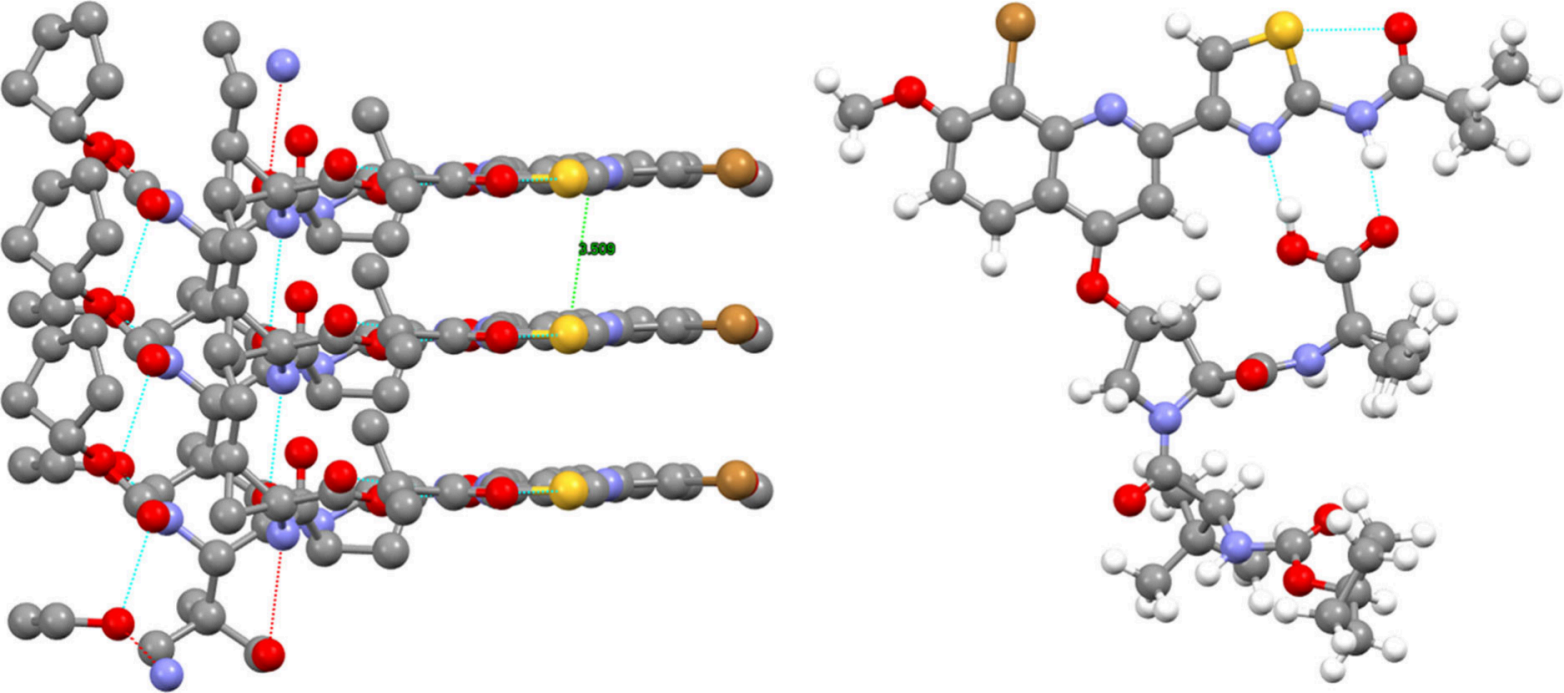
Molecular structure of BI201335 obtained from ED (right) and packing view of the crystal structure of BI201335 from in-house sc-XRD (left) with intramolecular hydrogen bonds highlighted.

**Figure 4:**
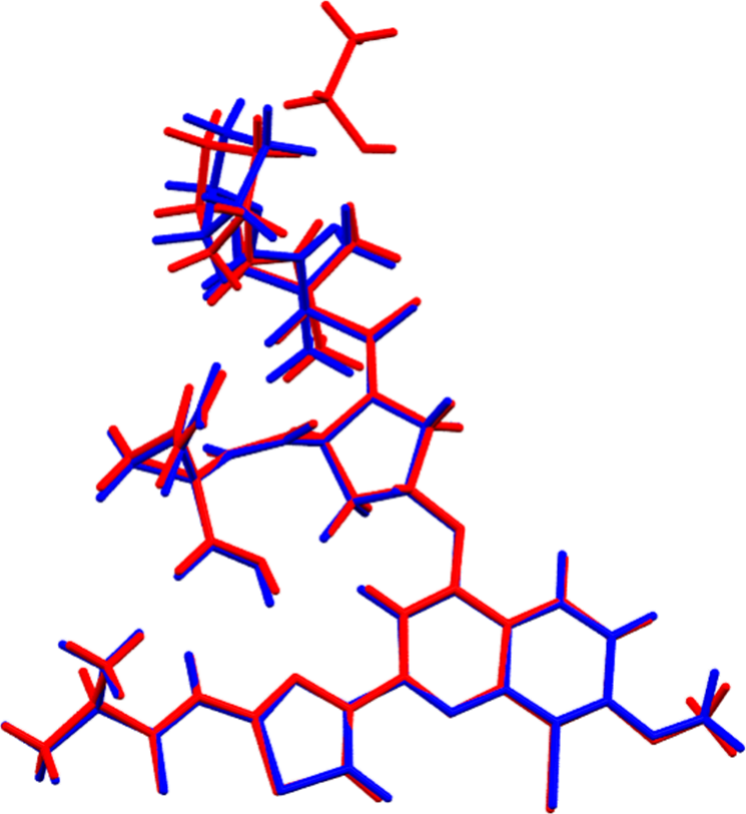
Overlay of the molecular structures of the ethanol solvate (from sc-XRD, red) and solvent-free form (from ED, blue) of BI201335.

This workflow—spanning approximate solubility testing, slow solvent evaporation, and thermal cycling—lays the groundwork for a more detailed exploration of the polymorphic landscape.

#### Advanced Polymorphic Screening for ACBI1

To address the challenges encountered in the initial screening, advanced methods such as thermal cycling with slurrying/ripening, antisolvent addition, solvent-drop grinding, and sublimation were employed:

- **Thermal Cycling with Slurrying/Ripening and Anti-Solvent Addition**: Solvents that formed slurries during thermal cycling generally led to more stable polymorphic forms, while antisolvent addition often resulted in metastable forms. Despite these systematic efforts, all samples analyzed via p-XRD remained amorphous (Table S3).
- **Solvent-Drop Grinding**: This high-energy mechanochemical method offered a rapid exploration of metastable forms. However, all tested samples resulted in amorphous material with no detectable crystalline polymorphic forms (Table S4).
- **Sublimation**: Sublimation under high vacuum was tested to force crystallization. The compound underwent a color change and transitioned to a gum-like material, but no crystalline material was observed, indicating a potential thermal chemical degradation.

When high-energy methods like solvent-drop grinding and sublimation failed to yield metastable polymorphic forms for the compound, the next step focused on exploring the potential for more stable polymorphic forms using the Encapsulated Nanodroplet Crystallization (ENaCt) protocol.^11^ This method, designed for high-throughput crystallization, is particularly suited for identifying stable polymorphic forms due to its extended crystallization timeframes.

#### Encapsulated Nanodroplet Crystallization (ENaCt) Protocol for ACBI1

Over 60 days, the concentration gradually increased, leading to spontaneous crystallization. Despite several successful experiments yielding small and larger single crystals (Figure 5), sc-XRD initially produced strong diffraction patterns, but the crystals either dissolved or degraded during measurement, precluding structural elucidation. Attempts to analyze small crystals by sending them to the ESRF Swiss Synchrotron resulted in poor-quality data sets, making it impossible to determine the crystal structure. In total, approximately 1600 experiments were conducted, with some producing poor-quality crystals that stopped diffracting during sc-XRD (Tables S5 – S17).

**Figure 5:**
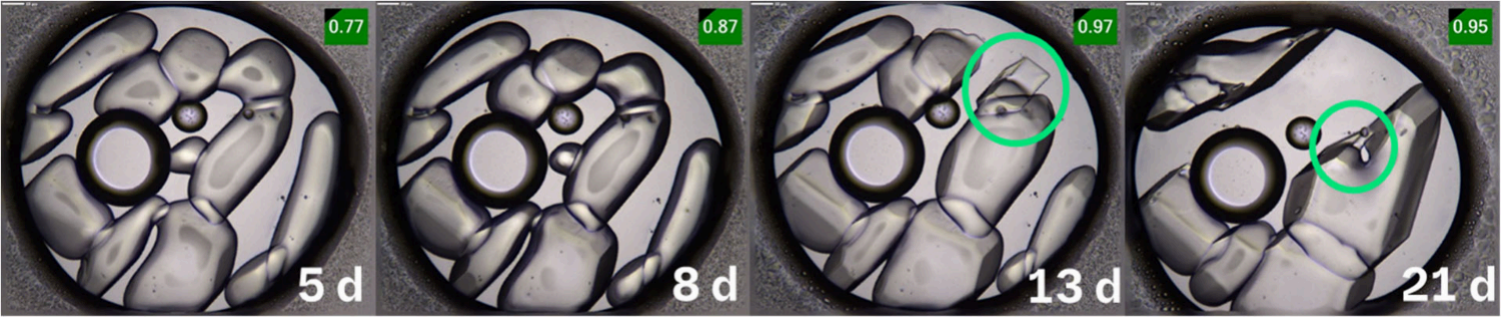
Encapsulated nanodroplet crystallization ACBI1 in DMSO 2 mg/mL and Fomblin YR images of 5, 8, 13, and 21 days. On the outer part of the image, the oil can be observed; inside, there is the solvent and an air pocket toward the left side; and in the end, the different shapes that can be observed are the compound that is aggregating and then forming crystals as seen at the 13th day, as indicated by the green circles.

#### Salt and Cocrystal Screening of ACBI1

As the polymorphic screen did not afford a suitable crystallization procedure for ACBI1, the experimental efforts were redirected toward salt and cocrystal screenings. The salt and cocrystal screening of ACBI1 aimed to improve the likelihood of forming stable crystalline salts or cocrystals, complementing the polymorphic screening efforts. Primary and extended salt and cocrystal screenings were performed, where acids were selected based on their compatibility with the basic p*K*_a_ of the API. The chosen acids offered a range of molecular weights and p*K*_a_ values to ensure diversity in the experimental conditions. For the cocrystal screening, weaker acids (coformers) were chosen to complement the API’s basic p*K*_a_ and enhance the potential for favorable interactions, such as π–π stacking and H-bonding as well as van der Waals and molecular complementarity (acting as a template). These steps were followed by solvent-drop grinding and vapor diffusion experiments to explore the crystallinity and polymorphic diversity of the salts and cocrystals.

#### Primary Salt Screen of ACBI1

Salt formation can significantly alter the physical properties of a compound, such as solubility, stability, and processability, making it an essential next step in the exploration of solid-state forms. Most samples exhibited amorphous characteristics, except for the combination of naphthalenedisulfonic acid with ethyl acetate, which produced a low-crystallinity diffractogram, suggesting the formation of the salt napadisylate (Tables S18–S23).

#### Extended Salt and Cocrystal Screening of ACBI1

Advancing the primary salt screen, an extended salt and cocrystal screening was performed. This phase aimed to explore a broader range of acids and coformers to improve the likelihood of forming stable crystalline salts or cocrystals with the API. The experimental procedure of the extended salt and cocrystal screening followed the established methodology from the primary salt screening (Tables S24–S47).

Low-to-medium crystallinity diffractograms were obtained for salts such as napadisylate, edisylate, and cyclamate, but most experiments resulted in amorphous or gum-like solids unsuitable for further analysis. Electron diffraction confirmed crystallinity in selected samples, allowing the determination of unit cells, although full structural elucidation was not possible.

#### Solvent Drop Grinding/Liquid Assisted Griding Salt Formation for ACBI1

Following the salt and cocrystal screening, the next step was a high-energy mechanochemical solvent drop grinding screen investigating whether different polymorphic forms of salts or cocrystals could be obtained, particularly those that might exhibit improved crystallinity or represent metastable states. While the results did not yield new polymorphic forms, the solvent-drop grinding improved the crystallinity of certain salts, such as napadisylate. However, some experiments still resulted in amorphous material or did not show notable improvements (Tables S48–S50).

#### Vapor Diffusion Salt Formation for ACBI1

Building on the insights gained from the solvent-drop grinding screening, the final experimental step involved vapor diffusion salt/cocrystal formation. This method’s effectiveness lies in its slow and controlled nature, which allows sufficient time for stable polymorphic forms to nucleate and grow. The timeline for crystallization depends on the rate of vapor diffusion, which is influenced by the properties of both the solvent and the antisolvent. Crystals were observed and collected, but sc-XRD and ED analyses revealed that the resultant materials were solvated forms of the acids rather than distinct salts or cocrystals. Diffractograms showed no differentiation between the already obtained forms and previously identified patterns (Tables S51–S52) as were described in the preceding experiments. Despite this, vapor diffusion experiments provided complementary insights into the thermodynamic stability of the salt and cocrystal forms, offering a broader understanding of the solid-state behavior of the compounds.

#### Crystallization Behavior of ACBI1 and BI201335

The polymorphic screening experiments revealed a stark contrast between the two compounds. BI201335 readily formed crystalline material using traditional methods such as thermal cycling, slurrying, and slow solvent evaporation, culminating in the identification of form I. Subsequent seeding experiments further improved the crystal quality, enabling structural elucidation via single crystal X-ray diffraction (sc-XRD) and electron diffraction (ED). These findings underscore the importance of seeding for enhancing the crystal quality when initial attempts yield suboptimal results.

After exploring a plethora of different experimental approaches to crystallize ACBI1, no crystalline material was obtained, only yielding amorphous material. Thermal cycling, antisolvent addition, solvent-drop grinding, sublimation, and the Encapsulated Nanodroplet Crystallization (ENaCt) protocol were systematically employed to explore the polymorphic landscape of ACBI1. While these approaches provided valuable insights into its solubility and stability, they failed to produce high-quality crystalline material, indicating an intrinsic difficulty in nucleation and crystal growth of this compound. This discrepancy prompted us to investigate the conformational behavior of the two molecules more closely, identifying the peptide bonds, which have been widely known for their partial double bond character. Free rotation around this bond is prevented due to the delocalization of the π-electron from the double-bonded oxygen toward the peptide bond.^84,85^

#### Molecular Mechanics with Machine Learning and DFT Calculations

The crystallization attempts investigated, with different approaches, the crystallization capacity of ACBI1. However, none of the attempts addressing different molecular properties allowed better crystallization conditions. Therefore, in the next step, we extensively screened the molecule flexibility with a MM-ML level method and further investigated the identified molecule parts of interest with a more expensive QM level method.

#### Identification of Potential Torsions of Interest via MM-ML Screening

During the torsion screening step, 20 nonterminal rotational bonds for ACBI1 and 17 for BI201335 were automatically detected, and their energetic rotation barriers were evaluated on the MM-ML level (Figures S1 and S2). All the rotational bonds were calculated in eight parallel jobs on our high-performance computing cluster, taking in total 18 min for ACBI1 and 13 min for BI201335.

In both molecules, three bonds were identified as potential atropisomerism causing bonds (Figure 6: ACBI-4, ACBI-6, ACBI-10, BI201335-2, BI201335-6, and BI201335-8). The rotational bonds were identified due to their energetic barrier heights (>13 kcal/mol) and profile shapes (≥2 clear minima) in Table 4.

**Figure 6:**
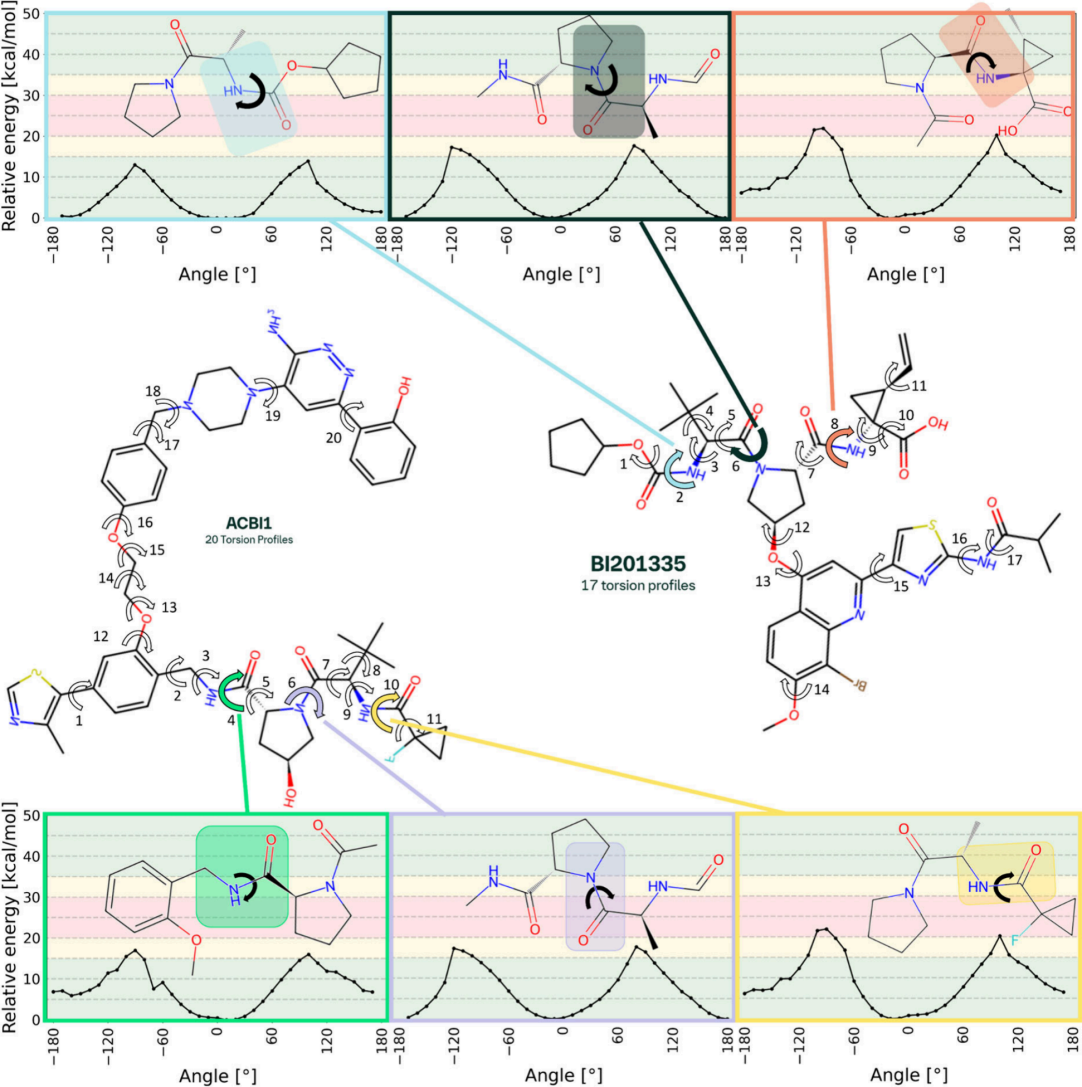
Torsion profile screening of molecule fragments from ACBI1 and BI201335 using machine learning molecular mechanics-based structure optimizations. For each rotatable bond, which is not a terminal bond, a torsion profile was calculated (arrows around bonds, see Figures S1 and S2). For efficiency reasons, each profile presents the relative energy profile of one automatically determined fragment, around the target rotatable bond (see 2D chemical structure in plots). The colored arrows indicate torsions of interest, which show a potential atropoisomeric character. This character is expressed by the curve form and barrier height. Class 1 atropisomerism is 10–20 kcal/mol, Class 2 is 20–30 kcal/mol, and Class 3 is 30 kcal/mol and above.

**Table 4:** Significant Profile Features of Presented Torsion Profiles^a^

		ML-MM with OFF MACE			QM on DFT level with wB97XD		
molecule	torsion profile	ΔΔ*V*_*min*_ [kcal/mol]	max(*V*_max_) [kcal/mol]	min(*V*_max_) [kcal/mol]	ΔΔ*V*_min_ [kcal/mol]	max(*V*_max_) [kcal/mol]	min(*V*_max_) [kcal/mol]
ACBI1	4/a	5.91	16.99	15.98	12.81	19.54	14.25
	6/b	0.54	16.67	15.28	3.68	23.44	22.90
	9/c	6.17	18.26	18.19	4.81	18.19	15.27
BI201335	2/a	0.29	13.89	12.95	0.38	12.8	11.88
	6/b	0.04	17.64	17.27	2.32	17.39	17.82
	8/c	6.97	21.92	20.21	3.20	19.84	17.82

a See Figure 5 and 6 and Figures S1 and S2. The values describe the estimated relative energy difference of the minima (*ΔΔV*_min_), the maximal rotational barrier height (max­(*V*_max_)) and the minimal rotational barrier height (min­(*V*_max_)) of a profile.

Note that the screening step is a fast procedure for identifying potential rotational bonds of interest. However, this step is prone to accuracy loss at the barrier’s heights, which is complicated to learn with machine learning approaches. Another aspect is the process inherent to molecule fragmentation, which increases the computational efficiency. This fragmentation is useful for an initial overview but can also limit the accuracy of the profiles.^76^

As a conclusion, we wanted to further investigate the identified potentially interesting rotational bonds with a more accurate approach to get the most accurate computational insight into the rotational barriers.

#### Validation with Larger Fragments on a QM-DFT Level

Encouraged by these results, we proceeded to perform quantum mechanics DFT-level calculations for a more precise determination of the rotational energy barriers, enabling us to understand the phenomenon at a deeper level (Figure 7). One torsion angle optimization calculation took for the large ACBI1 and BI020335 between 10 and 26 h. However, from these calculations one torsion of ACBI1-b was predicted as a potential atropisomerism causing a rotatable bond with two barriers of the height of 23.0 and 22.9 kcal/mol, whereas all other profiles have barrier heights below <20 kcal/mol and therefore are not predicted as potential Class 2 atropisomeric cases (Table 4). To experimentally verify these computational results, we employed variable temperature (VT) and 2D NMR spectroscopy (ROESY).

**Figure 7:**
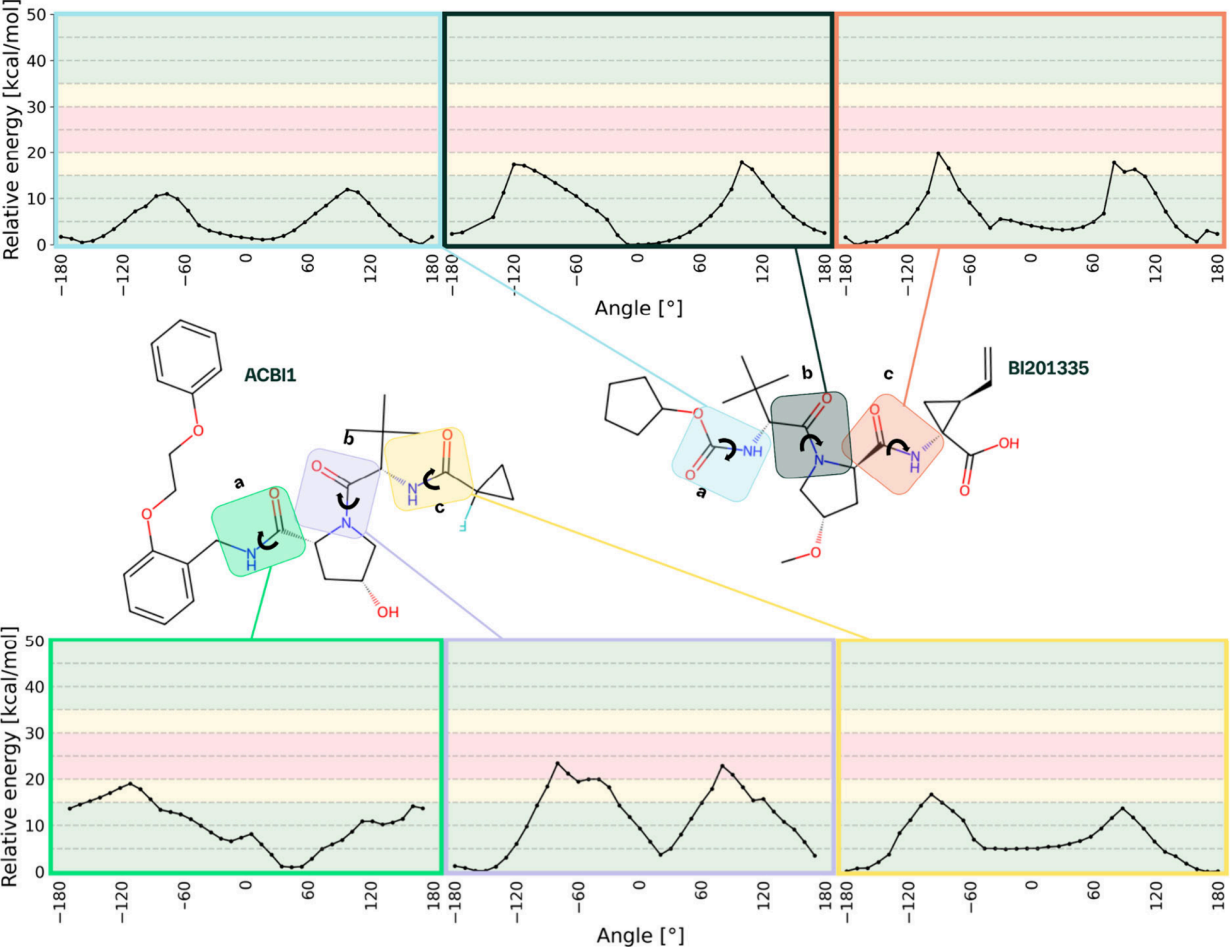
DFT-level torsion profiles of previously identified potential atropisomeric rotational bonds on larger fragments. In this step, a more accurate level of theory DFT with the method wB97XD is applied. For these calculations, larger fragments were used to represent the environment of the rotational bonds better. Only for ACBI1-b, a Class 2 atropisomeric bond was identified, serving as a potential rationale for the observed behavior. Class 1 atropisomerism is 10–20 kcal/mol, Class 2 is 20–30 kcal/mol, and Class 3 is 30 kcal/mol and above.

#### Variable Temperature NMR Rotational Barrier Calculation

The evaluation of the energy barrier for the single bond rotation by experiment serves as key information for the crystallization properties of the bRo5-like molecules. Single one-dimensional proton measurements at various temperatures in 10 K steps allow the assessment of the coalescence temperature and the calculation of the rotational barrier energy, as described below. Both model compounds, ACBI1 and BI201335, show a clearly observable doubled signal set for some of the signals in the NMR spectra at 303 K. This could be attributed to presence of rotamers and/or diastereoisomers, as shown in Figure 8 and Figure 9 for ACBI1 and Figure 10 and Figure 11 for BI201335. The discernment of whether the multiple of signal sets is observed due to a slow convergence between two or more forms or the presence of diastereoisomers is most easily accessed by performing a two-dimensional proton ROESY experiment. Performing the ROESY experiment for ACBI1 at 303 K (spectrum excerpt shown in Supporting Information, Figure S4) showed no clear evidence for the presence of rotameric species. This observation was crosschecked by repeating the ROESY experiment at 373 K as shown in Figure 9. At this temperature, the exchange between the proton signals could be clearly observed by the presence of cross peaks with the same phase as the diagonal peaks. The Nuclear Overhauser Enhancement (NOE) proximity cross peaks for small molecules have an antidiagonal phase. This serves as proof for the presence of rotamers with rotational barrier that could not be overcome at 303 K and therefore no exchange cross peaks are measured. As in the case of ACBI1 at 303 K, no exchange peaks could be observed and only the increased temperature allows the observation of exchange signals, revealing the existence of rotamers; the rotameric barrier is not reachable at 303 K. In addition, this means that at room temperature on the NMR time scale the rotameric pairs behave as separate stable-enough species, and they do not have enough energy to interconvert the barrier to a measurable extent. In contrast to the ACBI1, in the ROESY spectrum at 303 K for BI201335 the exchange cross peaks are measurable. In addition, a third signal set with broad resonances is observable for the BI201335 as demonstrated in the ROESY excerpt spectrum in Figure 11. This suggests a lower rotational energy barrier for BI201335.

**Figure 8:**
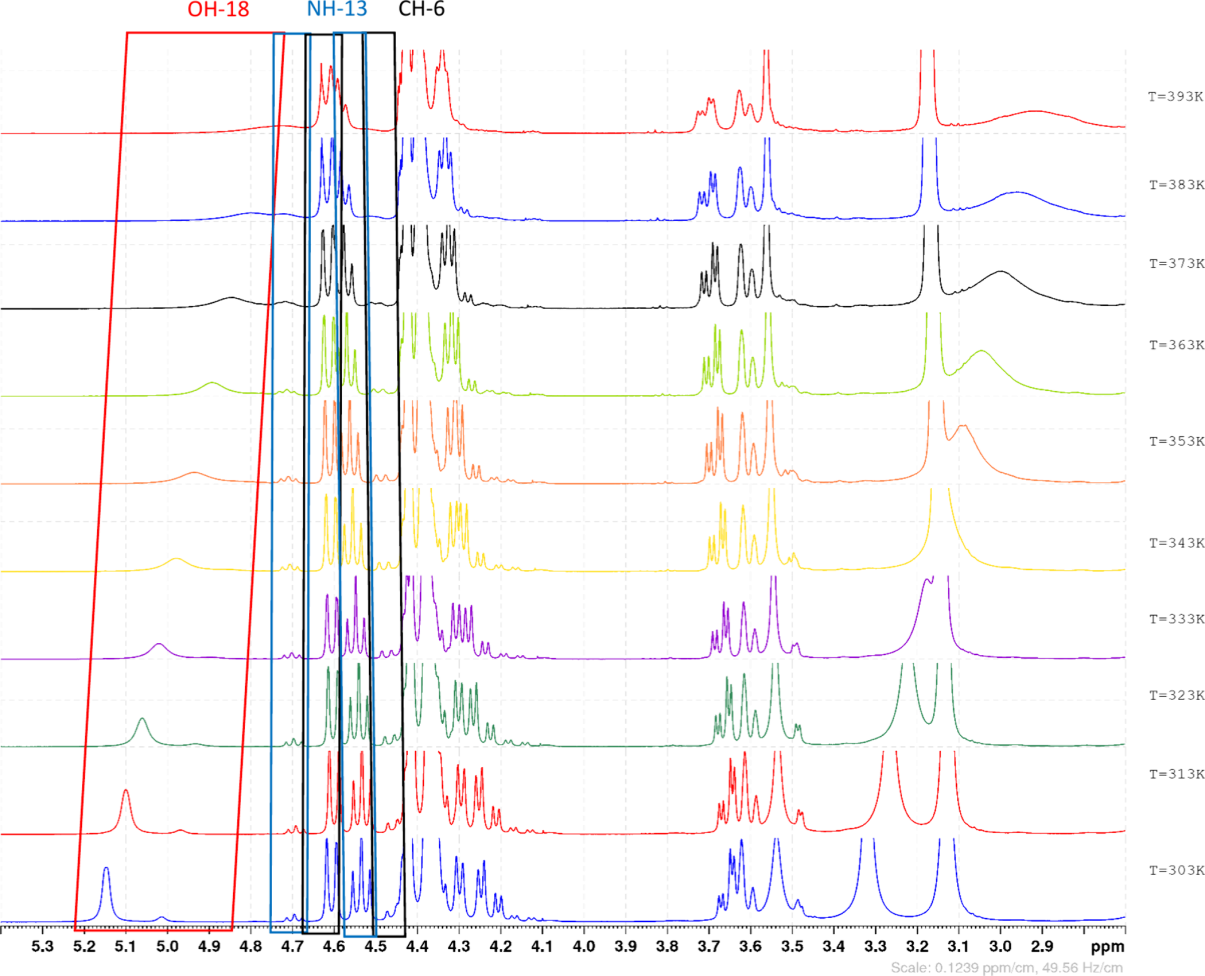
One-dimensional proton NMR experiments of ACBI1 performed by varying the measurement temperature in 10° steps, starting from 303 K (bottom spectrum) and finishing at 393 K (top spectrum). The double sets of signals at 4.53 and 4.69 ppm and 4.46 and 4.60 ppm vanish toward the baseline at 393 K as their coalescence temperature gets closer. Note that signal assignment is available in Table S56 in the Supporting Information.

**Figure 9:**
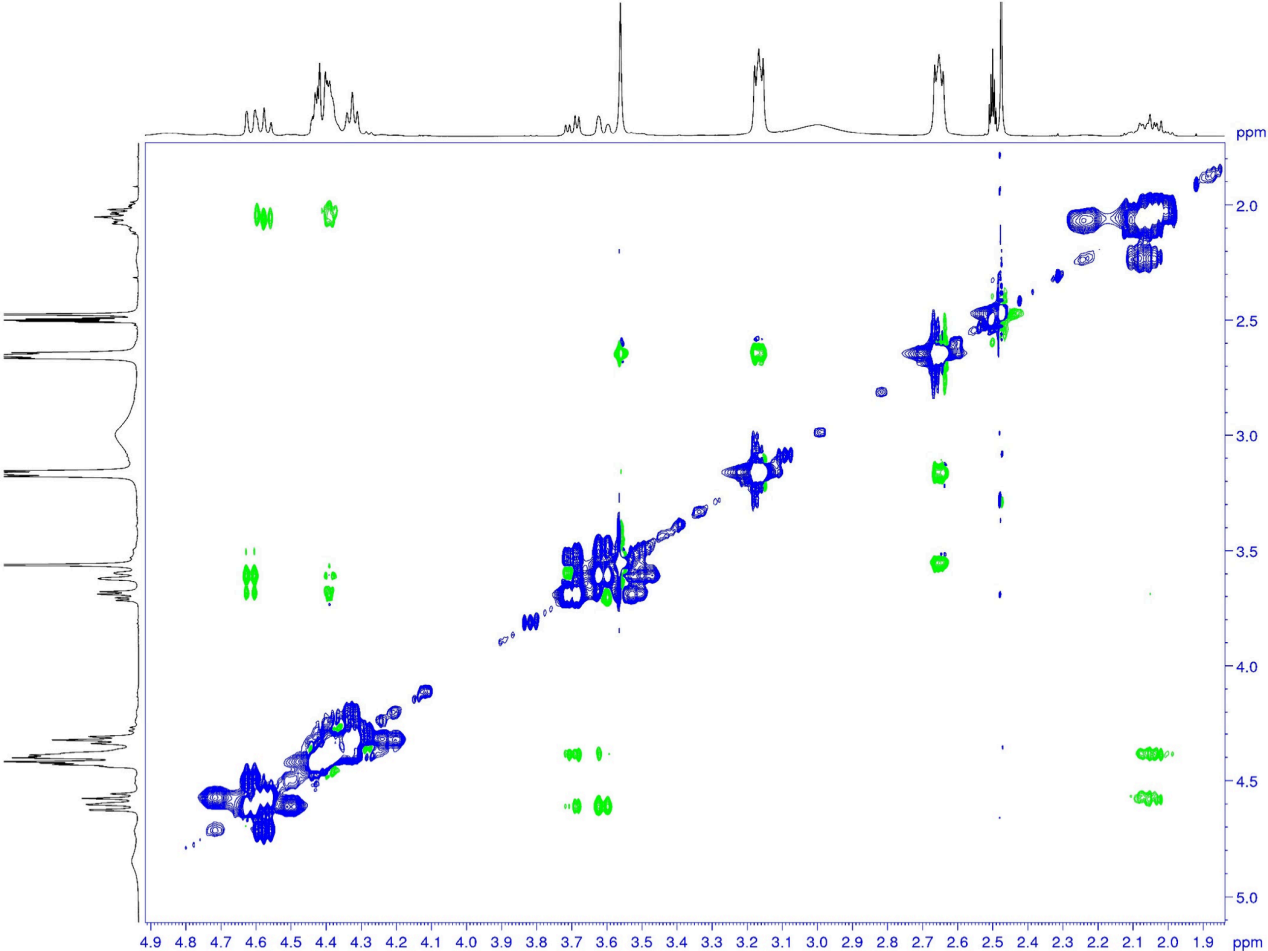
Two-dimensional proton ROESY NMR experiments of ACBI1 performed at 373 K, showing the exchange taking place between the double set of signals at 2.07 and 2.24 ppm as well as in the range between 3.45 and 3.74 ppm and between 4.18 and 4.76 ppm. The exchange cross peaks are colored in blue as the diagonal signals, while the NOE proximities over space have an antiphase with respect to the diagonal and are colored in green. Due to the complexity of the molecule, a third set of signals in the ROESY spectrum cannot be excluded. Note that signal assignment is available in Table S56 in the Supporting Information.

**Figure 10:**
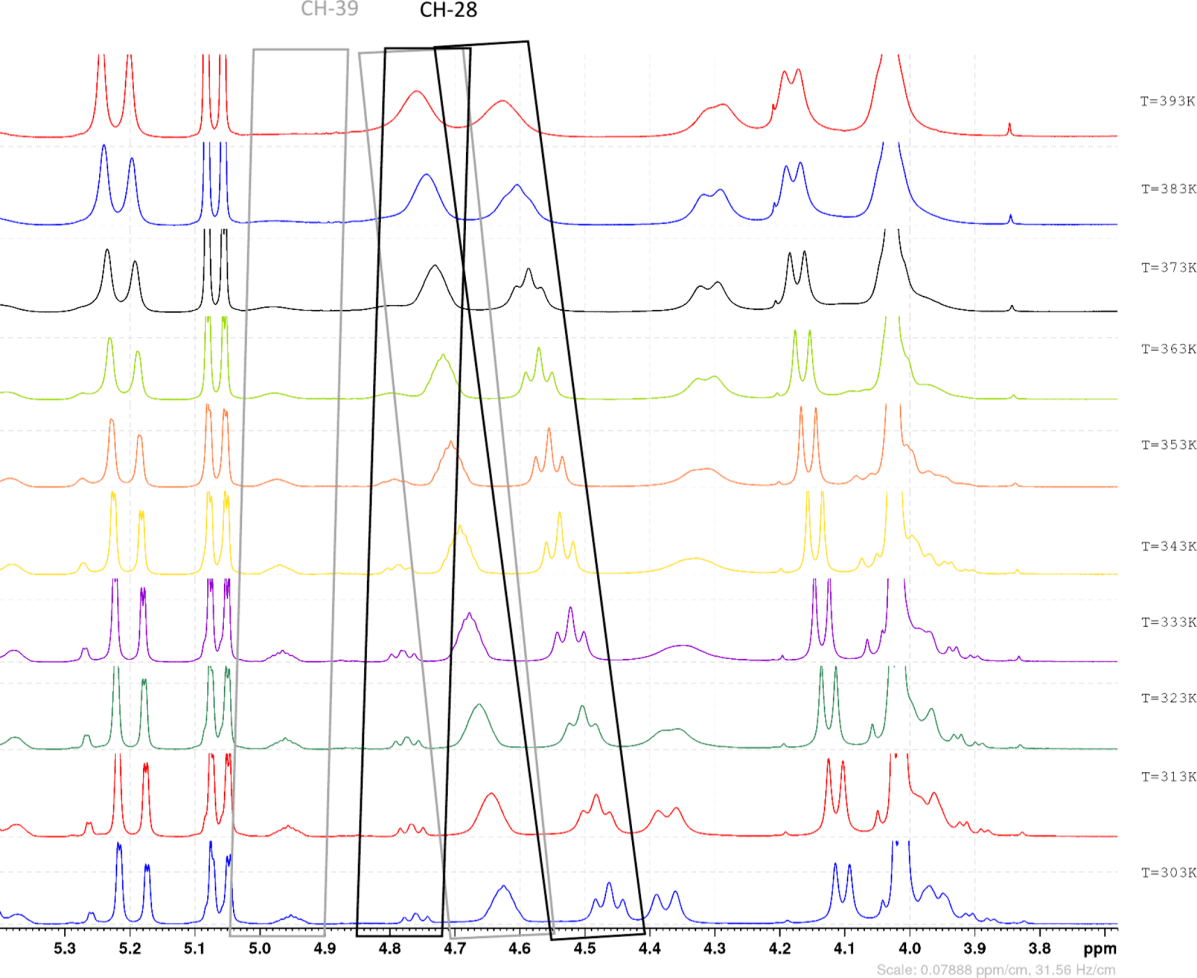
One-dimensional proton NMR experiments of BI201335 performed varying the measurement temperature in 10° steps, starting from 303 K (bottom spectrum) and finishing at 393 K (top spectrum). The double sets of signals at 4.62 and 4.95 ppm and 4.46 and 4.76 ppm vanish within the baseline at 393 K as their coalescence temperature is within reach. Note that signal assignment is available in Table S55 in the Supporting Information.

**Figure 11:**
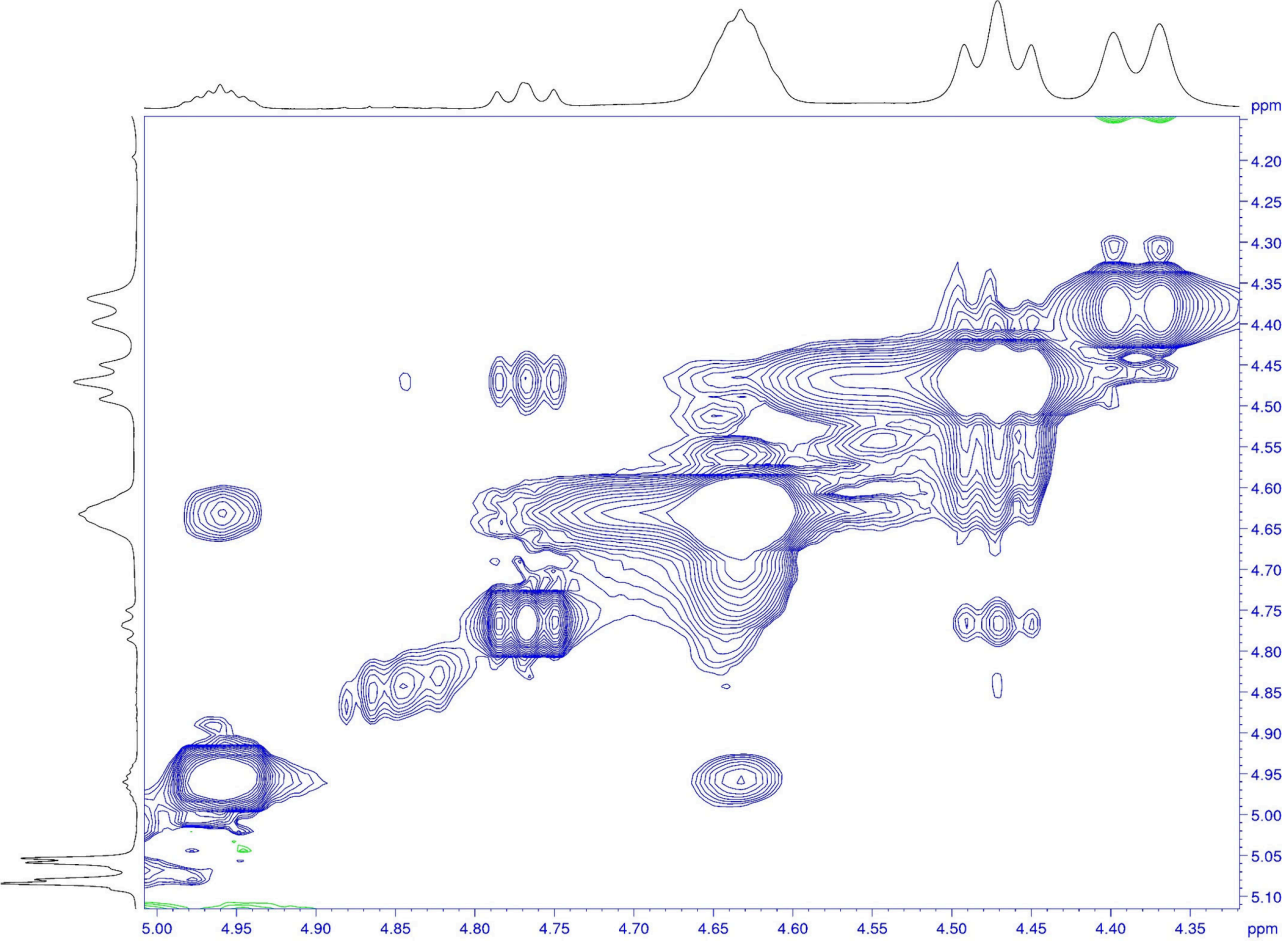
Two-dimensional proton ROESY NMR experiments of BI201335 performed at 303 K, showing the exchange taking place between the double set of signals at 4.62 and 4.95 ppm as well as at 4.46 and 4.76 ppm A third set of broad signals is identified with the help of the ROESY spectrum at the left shoulder of the more favorable rotamer signal set. The exchange cross peaks due to rotameric rotation are in-phase with the diagonal and are colored in blue. Note that signal assignment is available in Table S55 in the Supporting Information.

As indicated above, the two-dimensional proton ROESY experiments provide the proof that the multiple signal sets originate from a rotameric exchange. Thus, the next step is to calculate the rotational energy barrier for the single bonds, as described in the introduction, using the Eyring equation.

The variable temperature NMR experiments were performed in the temperature range from 303 to 393 K with a 10 K step in between the experiments. In the case of BI201335 there are two rotameric signal pairs for which an attempt to calculate the rotational barrier could be performed. The signal pair with chemical shifts of 4.46 and 4.76 ppm at 303 K shows a coalescence temperature of 393 K. This leads to a calculated rotational barrier of 18.9 kcal/mol using the modified Eyring equation. The second pair with chemical shifts of 4.62 and 4.95 ppm does not fully converge in one signal at 393 K; thus, its coalescence temperature was not reached. An estimation of the potential rotational barrier nevertheless allows an approximation within a range of energy barriers to be calculated: if the coalescence temperature was 393 K, the rotational barrier for this signal pair would be 18.8 kcal/mol. Since the convergence of the signals is not observed at 393 K, an estimation, based on the measured differences for the Larmor frequency between the signals over the experimental temperature range, suggests a value for the coalescence temperature around 403 K. In such a case the rotational energy barrier would be 19.3 kcal/mol. As noted above, the ROESY experiment for the ACBI1 showed no exchange signals at near room temperature (303 K) and a clear exchange could be measured merely at 373 K. The variable temperature experiments confirmed this experimental observation, as at 393 K the coalescence temperature could not be reached. The existing equipment does not allow safe measurements at higher temperatures; therefore, to provide tendencies for the size of the rotational barrier, the calculation was performed for a few temperature points. At the last measured point of 393 K (clearly below the experimental coalescence temperature), the rotational barrier would be 19.4 kcal/mol. At 423 K the calculated energy barrier would be 20.9 kcal/mol, and at 453 K it would be 22.4 kcal/mol. Based on the lack of exchange signals in the ROESY spectra at lower temperatures and the shape of the signals at 393 K, a coalescence temperature around 450 K seems most plausible. Thus, the rotational barrier for ACBI1 is estimated as 22.4 kcal/mol, with an error range of about 1–1.5 kcal/mol.

## Discussion

### *In Silico* Calculations of Atropisomerism and Its Impact on Crystallization

Using machine learning combined with molecular mechanics followed by *ab initio* density functional theory (DFT) calculations, we determined the rotational energy barriers for ACBI1 and BI201335. The contrasting behaviors of ACBI1 and BI201335 can be attributed to their atropisomeric classifications. ACBI1, classified as a Class 2 atropisomer, exhibited a rotational energy barrier of 23.4 kcal/mol, leading to slow interconversion between conformers. This conformational rigidity likely contributed to its amorphous nature during crystallization, as multiple competing conformers hindered stable nucleation. Conversely, BI201335, a Class 1 atropisomer with a lower rotational energy barrier of 19.8 kcal/mol, displayed rapid conformational interconversion, facilitating predictable crystallization pathways and the formation of stable polymorphs.

### NMR Studies of Atropisomerism and Its Impact on Crystallization

With the help of one-dimensional proton and two-dimensional NMR experiments, performed at various temperatures, we assessed the difference in the crystallization behavior in solution. The experimental conditions allowed the calculation of the rotation barrier for the BI201335. The calculation based on the coalescence temperature evaluation led to a value of 18.9 kcal/mol and lies in good agreement with the value calculated using DFT. This compound, BI201335, has a lower rotational energy barrier, which could, to some extent, be overcome at 303 K, showing the presence of a set of different rotamers as observed in the ROESY experiment. In contrast to BI201335, the experimental behavior of ACBI1 in the NMR experiments corresponded to much higher rotational barriers. This statement was supported by the lack of measurable exchange in the ROESY spectrum between the various rotamers at 303 K and the fact that the coalescence temperature could not be reached experimentally. The proof of the observation of rotamers and not a set of stereoisomers could be given in the ROESY experiment at 373 K, where the exchange peaks became visible. Thus, the NMR experiments provided clear evidence about the distinction of the rotational energy barrier size for BI201335 (below 20 kcal/mol) and ACBI1 (above 20 kcal/mol). This experimental observation supported the differences in the crystallization behavior of the two molecules.

### *In Silico* Calculations and Verdict of the NMR Study

The agreement of both methods supports our hypothesis of the conformational driven crystallization behaviors. The energy barrier difference between the two compounds has a profound impact on the conformational stability, interconversion rates, and ultimately the crystallization behavior of atropisomeric compounds, illustrating the sensitivity of these systems to even minor variations in energy barriers.

### Broader Implications

The findings emphasize the interplay between atropisomerism, molecular flexibility, and crystallization behavior. The energy difference between Class 1 and Class 2 atropisomers can potentially have effects on molecular conformational dynamics and solid-state properties. In this context, BI201335 was found to be a Class 1 atropisomer, and ACBI1 was a Class 2 atropisomer due to the energy barrier differences of approximately 3 kcal/mol between the rotational barriers of both molecules.

## Conclusions

Crystallization experiments, supported by various analytical techniques, allowed us to explore polymorphic forms and identify potentially suitable crystallization methods for challenging bRo5 compounds. The experiments emphasized the difficulties in crystallizing compounds that exhibit Class 2 atropisomerism and violate Lipinski’s Rule of 5 with peptide bonds.

Molecular mechanics, combined with machine learning and quantum mechanics DFT-level calculations, provided a quick and efficient way to identify Class 2 atropisomerism by predicting the stability and interconversion rates of atropisomers. This facilitated the early stage screening of compounds likely to be successful in later stages of drug development.

NMR spectroscopy, particularly VT-NMR and 2D ROESY NMR spectra, experimentally confirmed the rotational barrier energies identified by computational methods. These techniques validated the conformational states and hindered rotations of the peptide bonds, ensuring the reliable detection of Class 2 atropisomerism.

Our findings underscore the need to consider Class 2 atropisomerism in drug development, especially for bRo5 compounds, as well as the intermolecular interactions as were observed in BI201335, which give the structure more rigidity and enables crystallization. Early recognition and management of these challenges can lead to more efficient development pipelines. By adopting this multidimensional strategy, researchers can better navigate the complexities of modern drug discovery, optimizing the synthesis and crystallization of complex molecules for therapeutic use.

## Supplementary Material





## Notes

Experimental procedures for ACBI1 and BI201335, as described previously; computationally calculated rotational profiles for both molecules; assignment with 1D and 2D NMR of the under-investigation compounds; and analytical results, such as p-XRD, TGA, DSC, and DVS (PDF)
Low-resolution sc-XRD data for ethanol-solvated BI201335 (CIF)
