# Supplementary material for: Beyond Barriers, Big Crystallization Hurdles: Atropisomerism in Beyond Rule of Five Compounds Explored by Computational and NMR Studies
Source: Mol Pharm. 2025 Apr 27;22(6):3268–85. doi: 10.1021/acs.molpharmaceut.5c00204 (PMC12135943; doi:10.1021/acs.molpharmaceut.5c00204)
Supplement: Supplementary file 1 [file mp5c00204_si_001.pdf]

Beyond Barriers, Big Crystallisation Hurdles: Atropisomerism in bRo5 Compounds Explored by Computational and NMR Studies – Supplementary Information

Nikolaos Angelos Stamos,[1*] Benjamin Ries,[2] Regina Schneider,[3] Pavleta Tzvetkova,[3] Florian Montel,[4] Christian Jandl,[5] Ulrike Werthmann[1]

[1] CMC, DDS, Discovery Research, Boehringer Ingelheim Pharma GmbH & co. KG, 88400 Biberach an der Riss
[2] Computational Chemistry, Medicinal Chemistry, Discovery Research, Boehringer Ingelheim Pharma GmbH & co. KG, 88400 Biberach an der Riss
[3] Analytical Development, Development, Boehringer Ingelheim Pharma GmbH & co. KG, 88400 Biberach an der Riss
[4] Open Innovation, Medicinal Chemistry, Discovery Research, Boehringer Ingelheim Pharma GmbH & co. KG, 88400 Biberach an der Riss
[5] ELDICO Scientific AG, 4123 Allschwil, Switzerland

KEYWORDS: bRo5 compounds; Atropisomerism; Crystallisation; Rotational Barriers; in-silico calculations; NMR studies.

Experimental procedures ACBI1:

Approximate solvent solubility (ASS):

Approximately 20.00 mg were weighed in HPLC vials, and a magnetic stirrer bar was inserted. Water bath temperature was set at 40 °C, and the initial 25 µL of the appropriate solvent was added into the vial. Stirring was initiated and the slurries were brought up to the desired set temperature. Observations were recorded and the next addition was performed according to the Table S1. The samples were stirred at 40 °C overnight, cooled down to ambient conditions and observations were recorded after 24 h.

Table S1. Approximate Solvent Solubility (ASS) for ACBI1, with “x” indicating that a slurry was observed, while “o” indicated a clear solution (CS). Observations were recorded in two different timepoints.

ACBI1 - ASS -	Solvent	Mass (mg)	Volume of Solvent added (µL)										Initial Observations	Observations 24 h ambient
			50	16.7	13.3	20	33.3	66.7	66.7	133.3	400	1200		
1	1,4-Dioxane	20.32	x	x	o								Yellow CS	Yellow CS
2	1-Butanol	19.71	x	x	x	o							Yellow CS	Yellow CS
3	1-Propanol	20.18	x	x	o								Yellow CS	Yellow CS
4	2-Methyl THF	19.62	x	x	o								Yellow CS	Yellow CS
5	2-Propanol	19.72	x	x	x	o							Yellow CS	Solid + Yellow CS
6	48 % Methanol : 52 % Water (% v/v) (calculated a_w 0.8)	19.98	x	x	x	x	x	x	x	x	x	x	Yellow Gel + CS	Yellow Gel + CS
7	93 % Ethanol : 7 % Water (% v/v) (calculated a_w 0.5)	20.17	x	x	o								Yellow CS	Yellow CS
8	98.5 % Ethanol : 1.5 % Water (% v/v) (calculated a_w 0.2)	20.42	x	o									Yellow CS	Yellow CS
9	Acetone	19.6	o										Yellow CS	Yellow CS
10	Acetonitrile	19.55	x	x	x	x	x	x	x	x	x	x	Yellow Solid + CS	Yellow Solid + CS
11	Anisole	20.25	x	x	x	x	x	x	x	o			Yellow CS	Gum + Yellow CS
12	Dimethylsulfoxide	19.9	o										Yellow CS	Yellow CS
13	Ethanol	20.24	x	o									Yellow CS	Yellow CS
14	Ethyl Acetate	20.05	x	x	o								Yellow CS	Yellow CS
15	Heptane	20.44	x	x	x	x	x	x	x	x	x	x	Yellow Turbid Solution	Pale Yellow Slurry
16	Methanol	19.85	x	o									Yellow CS	Yellow CS
17	Methylethyl Ketone	19.67	x	o									Yellow CS	Yellow CS
18	Methylisobutyl Ketone	20.18	x	x	x	x	x	x	x	x	x	x	Yellow Solid + CS	Yellow CS
19	Cyclohexane	19.82	x	x	x	x	x	x	x	x	x	x	Yellow Turbid Solution	Pale Yellow Slurry
20	N,N'-Dimethylformamide	19.58	x	o									Yellow CS	Yellow CS
21	N-Methylpyrrolidone	20.33	x	x	x	x	o						Yellow CS	Yellow CS
22	*tert*-Butylmethyl Ether	20.13	x	x	x	x	x	x	x	x	x	x	Yellow Slurry	Yellow Slurry
23	Tetrahydrofuran	19.78	x	x	x	o							Yellow CS	Yellow CS
24	Toluene	20.43	x	x	x	x	x	x	x	x	x	x	Yellow Turbid Solution	Yellow Solid + CS
25	Water	19.46	x	x	x	x	x	x	x	x	x	x	Yellow Slurry Hydrophobic	Yellow Slurry Hydrophobic
Concentration (mg / mL)			400	300	250	200	150	100	75	50	25	10		

Slow solvent evaporation (SE):

Post approximate solvent solubility studies, the HPLC cap was pierced once, and the samples were stored into a cupboard for slow evaporation. After 14 days the samples afforded solids, which were analysed by p-XRD, while clear solutions were transferred to a vacuum oven and were slowly evaporated over the course of 24-h with step-wise pressure reduction (Table S2).

Table S2. Thermal Cycling (TC) and Slow Solvent Evaporation (SE) for ACBI1. Observations were recorded in different timepoints and p-XRD analysis was performed after the completion of the experiments.

ACBI1 - TC - SE -	Solvent	Observations	Observations 7 days ambient	Observations 14 days ambient	Observations 40 °C vac 24 h	p-XRD	
						Wet	Dry
1	1,4-Dioxane	Yellow CS	Beige Gum / Glass	Beige Gum / Solid	Beige Gummy Solid	Amorphous	Amorphous
2	1-Butanol	Yellow CS	Beige Gum / Glass	Beige Gum / Solid	Beige Gummy Solid	Amorphous	Amorphous
3	1-Propanol	Yellow CS	Beige Gum / Glass	Beige Gum / Solid	Beige Gummy Solid	Amorphous	Amorphous
4	2-Methyl THF	Yellow CS	Beige Gum / Glass	Beige Gum / Solid	Beige Gummy Solid	Amorphous	Amorphous
5	2-Propanol	Solid + Yellow CS	Yellow Gum / Glass	Beige Gum / Solid	Beige Gummy Solid	Amorphous	Amorphous
6	48 % Methanol : 52 % Water (% v/v) (calculated a_w 0.8)	Yellow Gel + CS	CS + Yellow Solid	CS + Yellow Solid	Beige Solid	Amorphous	Amorphous
7	93 % Ethanol : 7 % Water (% v/v) (calculated a_w 0.5)	Yellow CS	Beige Gum / Glass + Off White Solid	Beige Solid	Beige Solid	Amorphous	Amorphous
8	98.5 % Ethanol : 1.5 % Water (% v/v) (calculated a_w 0.2)	Yellow CS	Beige Gum / Glass + Off White Solid	Beige Solid	Beige Solid	Amorphous	Amorphous
9	Acetone	Yellow CS	Beige Gum / Glass	Beige Solid	Beige Solid	Amorphous	Amorphous
10	Acetonitrile	Yellow Solid + CS	Light Beige Solid	Beige Solid	Beige Solid	Amorphous	Amorphous
11	Anisole	Gum + Yellow CS	Yellow CS + Beige Gum	Yellow CS + Beige Gum	Beige Glass	Amorphous	Amorphous
12	Dimethylsulfoxide	Yellow CS	Beige Gum / Glass	Beige Gum / Glass	Beige Glass	Amorphous	Amorphous
13	Ethanol	Yellow CS	Beige Gum / Glass + Off White Solid	Beige Solid	Beige Solid	Amorphous	Amorphous
14	Ethyl Acetate	Yellow CS	Beige Gum / Glass + Off White Solid	Beige Solid	Beige Solid	Amorphous	Amorphous
15	Heptane	Pale Yellow Slurry	Off White Solid	Off White Solid	Off White Solid	Amorphous	Amorphous
16	Methanol	Yellow CS	Beige Solid	Beige Solid	Beige Solid	Amorphous	Amorphous
17	Methylethyl Ketone	Yellow CS	Beige Gum / Glass	Beige Gum / Glass	Beige Gummy Solid	Amorphous	Amorphous
18	Methylisobutyl Ketone	Yellow CS	Yellow CS + Beige Solid	Beige Solid	Beige Glass	Amorphous	Amorphous
19	Cyclohexane	Pale Yellow Slurry	Off White Solid	Off White Solid	Off White Solid	Amorphous	Amorphous
20	N,N'-Dimethylformamide	Yellow CS	Beige Gum / Glass	Beige Gum / Glass	Beige Gummy Solid	Amorphous	Amorphous
21	N-Methylpyrrolidone	Yellow CS	Beige CS	Beige CS	Brown Glass	Amorphous	Amorphous
22	*tert*-Butylmethyl Ether	Yellow Slurry	Off White Solid	Off White Solid	Off White Solid	Amorphous	Amorphous
23	Tetrahydrofuran	Yellow CS	Beige Gum / Glass	Beige Gum / Glass	Beige Gummy Solid	Amorphous	Amorphous
24	Toluene	Yellow Solid + CS	Yellow Solid	Yellow Solid	Yellow Solid	Amorphous	Amorphous
25	Water	Yellow Slurry Hydrophobic	CS + Off White Solid	Off White Solid	Off White Solid	Amorphous	Amorphous

Thermal cycling (TC) and Anti-Solvent Addition (ASA):

To the existing dry samples, 200 µL of the appropriate solvent were added at 40 °C under stirring. Observations were recorded (Table S3) and the following program was initiated and repeated under stirring and kept for 72 h:
2 h hold at 40 °C
Ramp down to RT with rate of 0.1 °C / min
2 h hold at RT
Ramp up to 40 °C with rate of 0.1 °C / min
Repeat
Post the 72-h thermal cycling, observations were recorded and slow anti-solvent addition, acetonitrile (MeCN), at 40 °C was performed to the clear solutions (CS) up to the point that a cloudy solution was observed. All samples were again placed for thermal cycling for 72 h. After the 72-h thermal cycling observation were taken (Table S3), and all samples were filter centrifuged and the solids were isolated. The clear solution was stored for slow evaporation, while the solid material was measured by p-XRD.

Table S3. Thermal Cycling (TC) and Anti-Solvent Addition (ASA) for ACBI1. Observations were recorded in different timepoints and p-XRD analysis was performed after the completion of the experiments.

ACBI1 - TC - ASA -	Solvent	Mass (mg)	Initial Observations	Post 72 h TC Observations	Anti-solvent	Volume of Anti-solvent added (mL)	Post-ASA TC Observations	p-XRD	
								Wet	Dry
1	1,4-Dioxane	20.32	Yellow CS	Yellow CS	MeCN	0.2	Beige Gum / Solid	Amorphous	Amorphous
2	1-Butanol	19.71	Yellow CS	Yellow CS	MeCN	0.25	Beige Gum / Solid	Amorphous	Amorphous
3	1-Propanol	20.18	Yellow CS	Yellow CS	MeCN	0.2	Beige Gum / Solid	Amorphous	Amorphous
4	2-Methyl THF	19.62	Yellow CS	Yellow CS	MeCN	0.25	Beige Gum / Solid	Amorphous	Amorphous
5	2-Propanol	19.72	Solid + Yellow CS	Solid + Yellow CS	N/A	N/A	Beige Gum / Solid	Amorphous	Amorphous
6	48 % Methanol : 52 % Water (% v/v) (calculated a_w 0.8)	19.98	Yellow Gel + CS	Yellow Gel + CS	N/A	N/A	Beige Solid	Amorphous	Amorphous
7	93 % Ethanol : 7 % Water (% v/v) (calculated a_w 0.5)	20.17	Yellow CS	Yellow CS	MeCN	0.2	Beige Solid	Amorphous	Amorphous
8	98.5 % Ethanol : 1.5 % Water (% v/v) (calculated a_w 0.2)	20.42	Yellow CS	Yellow CS	MeCN	0.15	Beige Solid	Amorphous	Amorphous
9	Acetone	19.6	Yellow CS	Yellow CS	MeCN	0.3	Beige Solid	Amorphous	Amorphous
10	Acetonitrile	19.55	Yellow Solid + CS	Yellow Solid + CS	N/A	N/A	Beige Solid	Amorphous	Amorphous
11	Anisole	20.25	Gum + Yellow CS	Gum + Yellow CS	MeCN	0.35	Beige Glass	Amorphous	Amorphous
12	Dimethylsulfoxide	19.9	Yellow CS	Yellow CS	MeCN	0.75	Yellow CS	Amorphous	Amorphous
13	Ethanol	20.24	Yellow CS	Yellow CS	MeCN	0.25	Beige Solid	Amorphous	Amorphous
14	Ethyl Acetate	20.05	Yellow CS	Yellow CS	MeCN	0.2	Beige Solid	Amorphous	Amorphous
15	Heptane	20.44	Pale Yellow Slurry	Pale Yellow Slurry	N/A	N/A	Off White Solid	Amorphous	Amorphous
16	Methanol	19.85	Yellow CS	Yellow CS	MeCN	0.25	Beige Solid	Amorphous	Amorphous
17	Methylethyl Ketone	19.67	Yellow CS	Yellow CS	MeCN	0.25	Beige Glass	Amorphous	Amorphous
18	Methylisobutyl Ketone	20.18	Yellow CS	Pale Yellow Slurry	N/A	N/A	Beige Glass	Amorphous	Amorphous
19	Cyclohexane	19.82	Pale Yellow Slurry	Pale Yellow Slurry	N/A	N/A	Off White Solid	Amorphous	Amorphous
20	N,N'-Dimethylformamide	19.58	Yellow CS	Yellow CS	MeCN	0.75	Yellow CS	Amorphous	Amorphous
21	N-Methylpyrrolidone	20.33	Yellow CS	Yellow CS	MeCN	0.75	Yellow CS	Amorphous	Amorphous
22	*tert*-Butylmethyl Ether	20.13	Yellow Slurry	Yellow Slurry	N/A	N/A	Off White Solid	Amorphous	Amorphous
23	Tetrahydrofuran	19.78	Yellow CS	Yellow CS	MeCN	0.3	Beige Glass	Amorphous	Amorphous
24	Toluene	20.43	Yellow Solid + CS	Yellow Solid + CS	N/A	N/A	Yellow Solid	Amorphous	Amorphous
25	Water	19.46	Yellow Slurry Hydrophobic	Yellow Slurry Hydrophobic	N/A	N/A	Off White Solid	Amorphous	Amorphous

Solvent Drop Grinding (SDG) or Liquid Assisted Grinding (LAG):

Approximately 20.00 mg were weighed in the suitable SDG plastic tubes, and three stainless steel metallic beads were inserted. Minimal amount of the appropriate solvent, 0.015 mL, was added and the tube was closed with the appropriate cap. Observations were recorded (Table S4) and the following program was initiated and repeated twice:
RPM: 4000
Intervals: 80 s milling, 10 s pause
Repetition: 10 times
Time: 15 min
Total time: 30 min
Post SDG, observations were recorded, and the material was transferred to the appropriate sample holders for p-XRD analysis.

Table S4. Solvent Drop Grinding (SDG) or Liquid Assisted Grinding (LAG) for ACBI1. Observations were recorded in different timepoints and p-XRD analysis was performed after the completion of the experiments.

ACBI1 - SDG -	Solvent	Mass (mg)	Volume of Solvent added (mL)	Observations	Observations 30 min SDG	p-XRD
1	1,1-Dimethoxymethane	19.81	0.015	Wet Yellow Solid	Yellow Glass	Amorphous
2	1,2-Dimethoxyethane	19.98	0.015	Wet Yellow Solid	Yellow Gum	Amorphous
3	1,4-Dioxane	19.65	0.015	Wet Yellow Solid	Yellow Gum	N/A
4	1-Butanol	19.83	0.015	Wet Yellow Solid	Yellow Gum	N/A
5	1-Propanol	19.98	0.015	Wet Yellow Solid	Yellow Gum	N/A
6	2-Methyl THF	19.95	0.015	Wet Yellow Solid	Yellow Gum	N/A
7	2-Propanol	19.69	0.015	Wet Yellow Solid	Yellow Gum	N/A
8	48 % Methanol : 52 % Water (% v/v) (calculated a_w 0.8)	19.89	0.015	Wet Yellow Solid	Yellow Gum	N/A
9	93 % Ethanol : 7 % Water (% v/v) (calculated a_w 0.5)	19.73	0.015	Wet Yellow Solid	Yellow Gum	N/A
10	98.5 % Ethanol : 1.5 % Water (% v/v) (calculated a_w 0.2)	19.86	0.015	Wet Yellow Solid	Yellow Gum	N/A
11	Acetone	19.99	0.015	Wet Yellow Solid	Yellow Gum	N/A
12	Acetonitrile	19.67	0.015	Wet Yellow Solid	Yellow Gum	N/A
13	Anisole	20.23	0.015	Wet Yellow Solid	Yellow Gum	N/A
14	Chloroform	19.77	0.015	Wet Yellow Solid	Yellow Solid	Amorphous
15	Cyclohexane	19.82	0.015	Wet Yellow Solid	White Solid	Amorphous
16	Dichloromethane	19.78	0.015	Wet Yellow Solid	Yellow Gum	N/A
17	Diethyl ether	19.77	0.015	Wet Yellow Solid	White Solid	Amorphous
18	Di*iso*propyl ether	19.77	0.015	Wet Yellow Solid	White Solid	Amorphous
19	Dimethylsulfoxide	20.05	0.015	Wet Yellow Solid	Orange Gum	N/A
20	Ethanol	19.65	0.015	Wet Yellow Solid	Yellow Gum	N/A
21	Ethyl acetate	20.14	0.015	Wet Yellow Solid	Yellow Gum	N/A
22	Heptane	19.75	0.015	Wet Yellow Solid	White Solid	Amorphous
23	Isobutyl acetate	19.99	0.015	Wet Yellow Solid	Yellow Solid	Amorphous
24	Isopropyl acetate	20.03	0.015	Wet Yellow Solid	Yellow Solid	Amorphous
25	Methanol	19.91	0.015	Wet Yellow Solid	Yellow Gum	N/A
26	Methyl Ethyl Ketone	19.81	0.015	Wet Yellow Solid	Yellow Gum	N/A
27	Methylisobutyl Ketone	19.76	0.015	Wet Yellow Solid	Yellow Gum	N/A
28	N,N'-Dimethylacetamide	19.69	0.015	Wet Yellow Solid	Yellow Gum	N/A
29	N,N'-Dimethylformamide	19.73	0.015	Wet Yellow Solid	Yellow Gum	N/A
30	n-Butyl acetate	19.74	0.015	Wet Yellow Solid	Yellow Gum	N/A
31	Nitrobenzene	20.13	0.015	Wet Yellow Solid	Yellow Gum	N/A
32	Nitromethane	20.27	0.015	Wet Yellow Solid	Yellow Gum	N/A
33	N-Methylpyrrolidone	19.69	0.015	Wet Yellow Solid	Yellow Gum	N/A
34	p-Xylene	19.66	0.015	Wet Yellow Solid	White Solid	Amorphous
35	*tert*-Butanol	19.98	0.015	Wet Yellow Solid	Yellow Solid	Amorphous
36	*tert*-Butylmethyl ether	20.23	0.015	Wet Yellow Solid	White Solid	Amorphous
37	Tetrahydrofuran	19.80	0.015	Wet Yellow Solid	Yellowish Solid	Amorphous
38	Toluene	19.71	0.015	Wet Yellow Solid	White Solid	Amorphous
39	Trifluoroethanol	19.68	0.015	Wet Yellow Solid	Yellow Gum	N/A
40	Water	19.85	0.015	Wet Yellow Solid	White Solid	Amorphous

Sublimation experiments

Approximately 20 mg of ACBI1 was placed in a tube and the appropriate cold finger apparatus was inserted. High vacuum was applied and water regulation at the cold finger. The sample was then gradually gently heated with 50 °C steps, and the step was reduced to 10 °C at 150 °C, due to melting point. At 170 °C, the sample started melting and becoming gum-like material, and with further heating it started changing colour, resulting to decomposition.

Encapsulated Nanodroplet Crystallisation (ENaCt Protocol)

Experimental procedure was followed accordingly.[1,2] Solvent systems, oil systems, API concentrations, and API : Oil ratio were the variables that were changed and adapted on the compound. Images were collected systematically from the Rock Imager 1000 regulated at room temperature with the program Rock Maker, both from Formulatrix. Post 60-day solvent diffusion in the oil, some micro-crystalline and small single crystals were observed, and single crystal crystallography was performed, which indicated diffraction peaks, with no structural elucidation due to the reducing intensity of the peaks over time.
The promising results were gathered, the experiments were repeated to obtain bigger single crystals suitable for single crystal crystallography, which was not achieved.

Table S5. Encapsulated Nanodroplet Crystallisation legend on results of the experiment. Ranging from red to green, indicating the acquisition and the suitability of the crystals obtained.

	Sample remains in solution
	Phase separation from solution and no solid
	Amorphous or micro-crystalline solids
	Small single crystals
	Large single crystals

Table S6. Encapsulated Nanodroplet Crystallisation for ACBI1. Concentration of the prepared solutions was 50 mg / ml, with a 3 : 1 ratio of Oil : Solution.

ACBI1 ENaCt		Oil 750 nL											
		FC40		PDMSO		Fomblin YR		Mineral		Paraffin		Fomblin Lac	
Solvents + API 250 nL 50 mg / mL	1,4-Dioxane												
	1-Butanol												
	1-Propanol												
	Acetone												
	Dimethylsulfoxide												
	Methanol												
	Methylethyl Ketone												
	N,N'-Dimethylformamide												

Table S7. Encapsulated Nanodroplet Crystallisation for ACBI1. Concentration of the prepared solutions was 25 mg / ml, with a 3 : 1 ratio of Oil : Solution.

ACBI1 ENaCt		Oil 750 nL											
		FC40		PDMSO		Fomblin YR		Mineral		Paraffin		Fomblin Lac	
Solvents + API 250 nL 25 mg / mL	1,4-Dioxane												
	1-Butanol												
	1-Propanol												
	Acetone												
	Dimethylsulfoxide												
	Methanol												
	Methylethyl Ketone												
	N,N'-Dimethylformamide												

Table S8. Encapsulated Nanodroplet Crystallisation for ACBI1. Concentration of the prepared solutions was 10 mg / ml, with a 3 : 1 ratio of Oil : Solution.

ACBI1 ENaCt		Oil 750 nL											
		FC40		PDMSO		Fomblin YR		Mineral		Paraffin		Fomblin Lac	
Solvents + API 250 nL 10 mg / mL	1,4-Dioxane												
	1-Butanol												
	1-Propanol												
	Acetone												
	Dimethylsulfoxide												
	Methanol												
	Methylethyl Ketone												
	N,N'-Dimethylformamide												

Table S9. Encapsulated Nanodroplet Crystallisation for ACBI1. Concentration of the prepared solutions was 5 mg / ml, with a 3 : 1 ratio of Oil : Solution.

ACBI1 ENaCt		Oil 750 nL											
		FC40		PDMSO		Fomblin YR		Mineral		Paraffin		Fomblin Lac	
Solvents + API 250 nL 5 mg / mL	1,4-Dioxane												
	1-Butanol												
	1-Propanol												
	Acetone												
	Dimethylsulfoxide												
	Methanol												
	Methylethyl Ketone												
	N,N'-Dimethylformamide												

Table S10. Encapsulated Nanodroplet Crystallisation for ACBI1. Concentration of the prepared solutions was 2 mg / ml, with a 3 : 1 ratio of Oil : Solution.

ACBI1 ENaCt		Oil 750 nL											
		FC40		PDMSO		Fomblin YR		Mineral		Paraffin		Fomblin Lac	
Solvents + API 250 nL 2 mg / mL	1,4-Dioxane												
	1-Butanol												
	1-Propanol												
	Acetone												
	Dimethylsulfoxide												
	Methanol												
	Methylethyl Ketone												
	N,N'-Dimethylformamide												

Table S11. Encapsulated Nanodroplet Crystallisation for ACBI1. Concentration of the prepared solutions was 50 mg / ml, with a 2 : 1 ratio of Oil : Solution.

ACBI1 ENaCt		Oil 500 nL											
		FC40		PDMSO		Fomblin YR		Mineral		Paraffin		Fomblin Lac	
Solvents + API 250 nL 50 mg / mL	1,4-Dioxane												
	1-Butanol												
	1-Propanol												
	Acetone												
	Dimethylsulfoxide												
	Methanol												
	Methylethyl Ketone												
	N,N'-Dimethylformamide												

Table S12. Encapsulated Nanodroplet Crystallisation for ACBI1. Concentration of the prepared solutions was 25 mg / ml, with a 2 : 1 ratio of Oil : Solution.

ACBI1 ENaCt		Oil 500 nL											
		FC40		PDMSO		Fomblin YR		Mineral		Paraffin		Fomblin Lac	
Solvents + API 250 nL 25 mg / mL	1,4-Dioxane												
	1-Butanol												
	1-Propanol												
	Acetone												
	Dimethylsulfoxide												
	Methanol												
	Methylethyl Ketone												
	N,N'-Dimethylformamide												

Table S13. Encapsulated Nanodroplet Crystallisation for ACBI1. Concentration of the prepared solutions was 10 mg / ml, with a 2 : 1 ratio of Oil : Solution.

ACBI1 ENaCt		Oil 500 nL											
		FC40		PDMSO		Fomblin YR		Mineral		Paraffin		Fomblin Lac	
Solvents + API 250 nL 10 mg / mL	1,4-Dioxane												
	1-Butanol												
	1-Propanol												
	Acetone												
	Dimethylsulfoxide												
	Methanol												
	Methylethyl Ketone												
	N,N'-Dimethylformamide												

Table S14. Encapsulated Nanodroplet Crystallisation for ACBI1. Concentration of the prepared solutions was 5 mg / ml, with a 2 : 1 ratio of Oil : Solution.

ACBI1 ENaCt		Oil 500 nL											
		FC40		PDMSO		Fomblin YR		Mineral		Paraffin		Fomblin Lac	
Solvents + API 250 nL 5 mg / mL	1,4-Dioxane												
	1-Butanol												
	1-Propanol												
	Acetone												
	Dimethylsulfoxide												
	Methanol												
	Methylethyl Ketone												
	N,N'-Dimethylformamide												

Table S15. Encapsulated Nanodroplet Crystallisation for ACBI1. Concentration of the prepared solutions was 2 mg / ml, with a 2 : 1 ratio of Oil : Solution.

ACBI1 ENaCt		Oil 500 nL											
		FC40		PDMSO		Fomblin YR		Mineral		Paraffin		Fomblin Lac	
Solvents + API 250 nL 2 mg / mL	1,4-Dioxane												
	1-Butanol												
	1-Propanol												
	Acetone												
	Dimethylsulfoxide												
	Methanol												
	Methylethyl Ketone												
	N,N'-Dimethylformamide												

Table S16. Encapsulated Nanodroplet Crystallisation for ACBI1. The partially successful experiments, light green and green, were replicated in 6- or 12- fold manner with a 3 : 1 ratio of Oil : Solution.

ACBI1 ENaCt	Oil 750 nL					
250 nL 50 mg / mL	Fomblin YR	Fomblin YR	Fomblin YR	Fomblin YR	Fomblin YR	Fomblin YR
Dimethylsulfoxide						
	Mineral	Mineral	Mineral	Mineral	Mineral	Mineral
250 nL 50 mg / mL	Fomblin YR	Fomblin YR	Fomblin YR	Fomblin YR	Fomblin YR	Fomblin YR
1-Butanol						
250 nL 50 mg / mL	PDMSO	PDMSO	PDMSO	PDMSO	PDMSO	PDMSO
1-Propanol						
250 nL 50 mg / mL	Mineral	Mineral	Mineral	Mineral	Mineral	Mineral
Methylethylketone						
	Fomblin Lac	Fomblin Lac	Fomblin Lac	Fomblin Lac	Fomblin Lac	Fomblin Lac
250 nL 10 mg / mL	Fomblin YR	Fomblin YR	Fomblin YR	Fomblin YR	Fomblin YR	Fomblin YR
1-Butanol						
250 nL 10 mg / mL	PDMSO	PDMSO	PDMSO	PDMSO	PDMSO	PDMSO
1-Propanol						
250 nL 10 mg / mL	Fomblin YR	Fomblin YR	Fomblin YR	Fomblin YR	Fomblin YR	Fomblin YR
Dimethylsulfoxide						
	Paraffin	Paraffin	Paraffin	Paraffin	Paraffin	Paraffin
250 nL 10 mg / mL	Mineral	Mineral	Mineral	Fomblin Lac	Fomblin Lac	Fomblin Lac
Methylethyl Ketone						
	Fomblin Lac	Fomblin Lac	Fomblin Lac	Fomblin Lac	Fomblin Lac	Fomblin Lac
250 nL 5 mg / mL	Fomblin YR	Fomblin YR	Fomblin YR	Paraffin	Paraffin	Paraffin
Dimethylsulfoxide						
250 nL 2 mg / mL	PDMSO	PDMSO	PDMSO	PDMSO	PDMSO	PDMSO
1-Propanol						
	Fomblin YR	Fomblin YR	Fomblin YR	Fomblin YR	Fomblin YR	Fomblin YR
250 nL 2 mg / mL	Fomblin YR	Fomblin YR	Fomblin YR	Fomblin YR	Fomblin YR	Fomblin YR
Dimethylsulfoxide						

Table S17. Encapsulated Nanodroplet Crystallisation for ACBI1. The partially successful experiments, light green and green, were replicated in 6- or 12-fold manner with a 4 : 1 ratio of Oil : Solution.

ACBI1 ENaCt	Oil 200 nL					
50 nL 50 mg / mL	Fomblin YR	Fomblin YR	Fomblin YR	Fomblin YR	Fomblin YR	Fomblin YR
Dimethylsulfoxide						
	Mineral	Mineral	Mineral	Mineral	Mineral	Mineral
50 nL 50 mg / mL	Fomblin YR	Fomblin YR	Fomblin YR	Fomblin YR	Fomblin YR	Fomblin YR
1-Butanol						
50 nL 50 mg / mL	PDMSO	PDMSO	PDMSO	PDMSO	PDMSO	PDMSO
1-Propanol						
50 nL 50 mg / mL	Mineral	Mineral	Mineral	Mineral	Mineral	Mineral
Methylethylketone						
	Fomblin Lac	Fomblin Lac	Fomblin Lac	Fomblin Lac	Fomblin Lac	Fomblin Lac
50 nL 10 mg / mL	Fomblin YR	Fomblin YR	Fomblin YR	Fomblin YR	Fomblin YR	Fomblin YR
1-Butanol						
50 nL 10 mg / mL	PDMSO	PDMSO	PDMSO	PDMSO	PDMSO	PDMSO
1-Propanol						
50 nL 10 mg / mL	Fomblin YR	Fomblin YR	Fomblin YR	Fomblin YR	Fomblin YR	Fomblin YR
Dimethylsulfoxide						
	Paraffin	Paraffin	Paraffin	Paraffin	Paraffin	Paraffin
50 nL 10 mg / mL	Mineral	Mineral	Mineral	Mineral	Mineral	Mineral
Methylethyl Ketone						
	Fomblin Lac	Fomblin Lac	Fomblin Lac	Fomblin Lac	Fomblin Lac	Fomblin Lac
50 nL 5 mg / mL	Fomblin YR	Fomblin YR	Fomblin YR	Paraffin	Paraffin	Paraffin
Dimethylsulfoxide						
50 nL 2 mg / mL	PDMSO	PDMSO	PDMSO	PDMSO	PDMSO	PDMSO
1-Propanol						
	Fomblin YR	Fomblin YR	Fomblin YR	Fomblin YR	Fomblin YR	Fomblin YR
50 nL 2 mg / mL	Fomblin YR	Fomblin YR	Fomblin YR	Fomblin YR	Fomblin YR	Fomblin YR
Dimethylsulfoxide						

Salt and co-crystal screening

General procedure for salt and co-crystal screening

Stock solution of ACBI1 was prepared, with 100 mg / mL concentration, in the appropriate solvent systems, and was distributed to 24 HPLC vials. Stock solutions of the appropriate acids for the salt formations were prepared, which are targeting the basic pK_a centre of the molecule. The equivalents of acid to API were 1.05 : 1.00 from the 0.1 M acid stock solutions. The experimental procedure followed for the primary and extended salt screenings performed on the compound was:

- 200 µL of stock solution was transferred to the appropriate labelled vial.
- A stirrer bar was added, and the solution was stirred (250 rpm) at 25 °C.
- 224.3 µL/mg of acid 0.1M was added. Observation was recorded in appropriate table.
- Thermally cycled; heat to 40 °C at 0.1 °C/min, 2 h at 40 °C, cool to 25 °C at 0.1 °C/min, 2 h at 25 °C, repeat.
- After 72 h of thermal cycling, the samples were cooled at ambient conditions for 2 h.
- Slurries or Solid samples were centrifuged and analysed by p-XRD.
- CS samples were retained, and ASA was performed with the appropriate Anti-Solvent as indicated in the appropriate table and were thermally cycled as per above.
- Slurries or Solid samples post-ASA were analysed by p-XRD and CS samples were slowly evaporated to afford material suitable for p-XRD analysis.

Primary salt screening

Six acids in four different solvents were selected according to the pKa of the molecule, which are presented in Tables S18 – S23.

Table S18. Primary Salt Screening of ACBI1 with Hydrochloric Acid 0.1 M in four different solvent systems and two Anti-Solvents for the Anti-Solvent Addition (ASA) experiments, and Slow Evaporation (SE) experiments for the Clear Solutions (CS).

Counterion	Mass (mg)	Solvent	Observation Initial	Observation 3 Day	Anti-Solvent	Volume (mL)	Observation	Observation 3 Day	SE Observation 3 day	p-XRD Wet
HCl 0.1 M H_2O	19.48	MeOH : H_2O 80 / 20 % v/v	Yellow CS	Yellow CS	MeCN	1.5	Yellow CS	Yellow CS	Beige Solid	Amorphous
	20.29	Acetone	Yellow CS	Yellow CS	tBME	0.4	CS + Beige Gum	CS + Beige Gum	Beige Solid	Amorphous
	19.97	Ethyl Acetate	CS + Yellow Solid	CS + Beige Gum	tBME	0.5	CS + Beige Gum	CS + Beige Gum	Beige Solid	Amorphous
	20.18	THF	Yellow CS	Yellow CS	tBME	0.4	CS + Beige Gum	CS + Beige Gum	Beige Solid	Amorphous

Table S19. Primary Salt Screening of ACBI1 with 1,5-Naphthalenedisulfonic Acid 0.1 M in four different solvent systems and two Anti-Solvents for the Anti-Solvent Addition (ASA) experiments, and Slow Evaporation (SE) experiments for the Clear Solutions (CS).

Counterion	Mass (mg)	Solvent	Observation Initial	Observation 3 Day	Anti-Solvent	Volume (mL)	Observation	Observation 3 Day	SE Observation 3 day	p-XRD Wet
1,5-Naphthalenedisulfonic acid 0.1 M in MeOH	19.66	MeOH : H_2O 80 / 20 % v/v	Yellow CS	CS + Beige Gum	MeCN	N/A	N/A	N/A	Off White Solid	Amorphous
	19.73	Acetone	CS + Beige Gum	CS + Beige Gum	tBME	N/A	N/A	N/A	Off White Solid	Amorphous
	19.85	Ethyl Acetate	Yellow CS + White Solid	White Slurry + White Solid	tBME	N/A	N/A	N/A	N/A	Napadisylate Salt
	19.48	THF	Yellow CS + White Solid	CS + Beige Gum	tBME	N/A	N/A	N/A	Off White Solid	Amorphous

Table S20. Primary Salt Screening of ACBI1 with p-Toluenesulfonic Acid 0.1 M in four different solvent systems and two Anti-Solvents for the Anti-Solvent Addition (ASA) experiments, and Slow Evaporation (SE) experiments for the Clear Solutions (CS).

Counterion	Mass (mg)	Solvent	Observation Initial	Observation 3 Day	Anti-Solvent	Volume (mL)	Observation	Observation 3 Day	SE Observation 3 day	p-XRD Wet
p-Toluenesulfonic acid 0.1 M in THF	19.6	MeOH : H_2O 80 / 20 % v/v	Yellow CS	Yellow CS	MeCN	1.5	Yellow CS	Yellow CS	Beige Gum	N/A
	19.73	Acetone	Yellow CS	Yellow CS	tBME	0.4	CS + Beige Gum	CS + Beige Glass	N/A	Amorphous
	20.07	Ethyl Acetate	Yellow CS + White Solid	CS + Beige Gum	tBME	0.3	CS + Beige Gum + White Solid	CS + Beige Glass	N/A	Amorphous
	19.65	THF	Yellow CS	Yellow CS	tBME	0.2	CS + Beige Gum	CS + Beige Gum	Beige Gum	N/A

Table S21. Primary Salt Screening of ACBI1 with 2-Naphthalenesulfonic Acid 0.1 M in four different solvent systems and two Anti-Solvents for the Anti-Solvent Addition (ASA) experiments, and Slow Evaporation (SE) experiments for the Clear Solutions (CS).

Counterion	Mass (mg)	Solvent	Observation Initial	Observation 3 Day	Anti-Solvent	Volume (mL)	Observation	Observation 3 Day	SE Observation 3 day	p-XRD Wet
2-Naphthalenesulfonic acid 0.1 M in THF	19.72	MeOH : H_2O 80 / 20 % v/v	Yellow CS	Yellow CS	MeCN	0.8	White Slurry	CS + Beige Gum	Beige Gum	N/A
	19.6	Acetone	Yellow CS + Beige Solid	Pale Yellow CS + Beige Gum	tBME	0.2	CS + Beige Solid	CS + Beige Glass	N/A	Amorphous
	19.48	Ethyl Acetate	CS + Beige Solid	CS + Brown Solid	tBME	N/A	N/A	N/A	N/A	Amorphous
	19.53	THF	Yellow CS	Yellow CS	tBME	0.2	White Slurry + Beige Solid	CS + Beige Glass	N/A	Amorphous

Table S22. Primary Salt Screening of ACBI1 with Oxalic Acid 0.1 M in four different solvent systems and two Anti-Solvents for the Anti-Solvent Addition (ASA) experiments, and Slow Evaporation (SE) experiments for the Clear Solutions (CS).

Counterion	Mass (mg)	Solvent	Observation Initial	Observation 3 Day	Anti-Solvent	Volume (mL)	Observation	Observation 3 Day	SE Observation 3 day	p-XRD Wet
Oxalic acid 0.1 M in THF	19.59	MeOH : H_2O 80 / 20 % v/v	Yellow CS	Yellow CS	MeCN	1.5	Yellow CS	Yellow CS	Yellow Solid	Amorphous
	19.47	Acetone	Yellow CS + Yellow Solid	Off White Slurry	tBME	N/A	N/A	N/A	N/A	Low Crystallinity
	20.02	Ethyl Acetate	White Beige Slurry	Off White Slurry + Yellow Solid	tBME	N/A	N/A	N/A	N/A	Amorphous
	19.42	THF	Yellow CS	Off White Slurry	tBME	N/A	N/A	N/A	N/A	Low Crystallinity

Table S23. Primary Salt Screening of ACBI1 with Phosphoric Acid 0.1 M in four different solvent systems and two Anti-Solvents for the Anti-Solvent Addition (ASA) experiments, and Slow Evaporation (SE) experiments for the Clear Solutions (CS).

Counterion	Mass (mg)	Solvent	Observation Initial	Observation 3 Day	Anti-Solvent	Volume (mL)	Observation	Observation 3 Day	SE Observation 3 day	p-XRD Wet
Phosphoric acid 0.1 M in THF	19.55	MeOH : H_2O 80 / 20 % v/v	Yellow CS	Yellow CS	MeCN	0.9	White Slurry	CS + Yellow Gum	Yellow Solid	Amorphous
	20.08	Acetone	White Beige Slurry	Off White Slurry + Yellow Solid	tBME	N/A	N/A	N/A	N/A	Amorphous
	20.55	Ethyl Acetate	White Beige Slurry	Off White Slurry	tBME	N/A	N/A	N/A	N/A	Amorphous
	19.64	THF	White Beige Slurry	CS + Yellow Solid	tBME	N/A	N/A	N/A	N/A	Amorphous

Extended salt screening

24 acids in four different solvents were selected according to the pKa of the molecule, which are presented in Tables S24 – S47.

Table S24. Extended Salt Screening of ACBI1 with Hydrochloric Acid 0.1 M in four different solvent systems and one Anti-Solvent for the Anti-Solvent Addition (ASA) experiments, and Slow Evaporation (SE) experiments for the Clear Solutions (CS).

Counterion	Solvent	Observation Initial	Observation 3 Day	Anti-Solvent	Volume (mL)	Observation	Observation 3 Day	p-XRD Wet
HCl 0.1 M H_2O	2-Propanol	Yellow CS	Yellow CS	tBME	0.2	Yellow CS + Yellow Solid	CS + Beige Solid	Gum
	Acetone	Yellow CS	Yellow CS	tBME	0.1	White Slurry	CS + Beige Solid	Gum
	Ethyl Acetate	Yellow CS + White Solid	Yellow CS + White Solid	tBME	0.2	White Slurry	CS + Yellow Solid	Amorphous
	THF	Yellow CS	Orange CS	tBME	0.1	CS + Beige Solid	CS + Brown Solid	Gum

Table S25. Extended Salt Screening of ACBI1 with Hydrobromic Acid 0.1 M in four different solvent systems and one Anti-Solvent for the Anti-Solvent Addition (ASA) experiments, and Slow Evaporation (SE) experiments for the Clear Solutions (CS).

Counterion	Solvent	Observation Initial	Observation 3 Day	Anti-Solvent	Volume / mL	Observation	Observation 3 Day	p-XRD Wet
HBr 0.1 M H_2O	2-Propanol	Yellow CS	Yellow CS	tBME	0.3	Off White Slurry	CS + Beige Solid	Gum
	Acetone	Yellow CS	Yellow CS	tBME	0.2	Off White Slurry	CS + Beige Solid	Gum
	Ethyl Acetate	Yellow CS	Yellow CS	tBME	0.1	Off White Slurry	CS + Yellow Solid	Amorphous
	THF	Yellow CS	Yellow CS	tBME	0.2	Off White Slurry	CS + Beige Solid	Gum

Table S26. Extended Salt Screening of ACBI1 with 1,5-Naphthalenedisulfonic Acid 0.1 M in four different solvent systems and one Anti-Solvent for the Anti-Solvent Addition (ASA) experiments, and Slow Evaporation (SE) experiments for the Clear Solutions (CS).

Counterion	Solvent	Observation Initial	Observation 3 Day	Anti-Solvent	Volume / mL	Observation	Observation 3 Day	p-XRD Wet
1,5-Naphthalenedisulfonic acid 0.1 M in MeOH	2-Propanol	White Slurry + Yellow Solid	White Slurry + Yellow Solid	tBME	N/A	N/A	White Slurry + Beige Solid	Amorphous
	Acetone	CS + Yellow Solid	CS + Yellow Solid	tBME	N/A	N/A	CS + Beige Solid	Amorphous
	Ethyl Acetate	Yellow Hazy Solution	Yellow Hazy Solution	tBME	0.2	White Slurry	White Slurry + Beige Solid	Napadisylate Pattern 1
	THF	CS + Yellow Solid	CS + Yellow Solid	tBME	N/A	N/A	CS + Beige Solid	Amorphous

Table S27. Extended Salt Screening of ACBI1 with Sulfuric Acid 0.1 M in four different solvent systems and one Anti-Solvent for the Anti-Solvent Addition (ASA) experiments, and Slow Evaporation (SE) experiments for the Clear Solutions (CS).

Counterion	Solvent	Observation Initial	Observation 3 Day	Anti-Solvent	Volume / mL	Observation	Observation 3 Day	p-XRD Wet
Sulfuric acid 0.1 M in THF	2-Propanol	White Slurry + Yellow Solid	CS + Yellow Solid	tBME	N/A	N/A	CS + Beige Solid	Amorphous
	Acetone	Yellow CS + Yellow Solid	CS + Yellow Solid	tBME	N/A	N/A	CS + Beige Solid	Amorphous
	Ethyl Acetate	Yellow CS + Yellow Solid	CS + Yellow Solid	tBME	N/A	N/A	CS + Beige Solid	Amorphous
	THF	Yellow CS + Yellow Solid	CS + Yellow Solid	tBME	N/A	N/A	CS + Beige Solid	Amorphous

Table S28. Extended Salt Screening of ACBI1 with 1,2-Ethanedisulfonic Acid 0.1 M in four different solvent systems and one Anti-Solvent for the Anti-Solvent Addition (ASA) experiments, and Slow Evaporation (SE) experiments for the Clear Solutions (CS).

Counterion	Solvent	Observation Initial	Observation 3 Day	Anti-Solvent	Volume / mL	Observation	Observation 3 Day	p-XRD Wet
1,2-Ethanedisulfonic Acid 0.1 M in THF	2-Propanol	White Slurry	White Slurry	tBME	N/A	N/A	White Slurry	Edisylate Pattern 1?
	Acetone	White Slurry	White Slurry	tBME	N/A	N/A	White Slurry	Edisylate Pattern 1
	Ethyl Acetate	White Slurry	White Slurry	tBME	N/A	N/A	White Slurry	Edisylate Pattern 1
	THF	White Slurry	White Slurry	tBME	N/A	N/A	White Slurry	Edisylate Pattern 1?

Table S29. Extended Salt Screening of ACBI1 with Cyclamic Acid 0.1 M in four different solvent systems and one Anti-Solvent for the Anti-Solvent Addition (ASA) experiments, and Slow Evaporation (SE) experiments for the Clear Solutions (CS).

Counterion	Solvent	Observation Initial	Observation 3 Day	Anti-Solvent	Volume / mL	Observation	Observation 3 Day	p-XRD Wet
Cyclamic Acid 0.1 M in THF	2-Propanol	Off White Slurry	Yellow Slurry	tBME	N/A	N/A	Off White Slurry	Predominantly Amorphous
	Acetone	CS + Off White Solid	Yellow Solid	tBME	N/A	N/A	CS + Beige Solid	Cyclamate Pattern 1
	Ethyl Acetate	Off White Slurry + Off White Solid	Off White Slurry + Yellow Solid	tBME	N/A	N/A	White Slurry + Beige Solid	Cyclamate Pattern 1 + 2
	THF	Yellow CS	Yellow Thin Slurry	tBME	0.1	White Slurry	CS + Beige Solid	Cyclamate Pattern 2

Table S30. Extended Salt Screening of ACBI1 with Ethanesulfonic Acid 0.1 M in four different solvent systems and one Anti-Solvent for the Anti-Solvent Addition (ASA) experiments, and Slow Evaporation (SE) experiments for the Clear Solutions (CS).

Counterion	Solvent	Observation Initial	Observation 3 Day	Anti-Solvent	Volume / mL	Observation	Observation 3 Day	p-XRD Wet
Ethanesulfonic Acid 0.1 M in THF	2-Propanol	Yellow CS	Yellow CS	tBME	0.2	White Slurry	CS + Beige Solid	Gum
	Acetone	Yellow CS + Yellow Solid	CS + Orange Solid	tBME	N/A	N/A	CS + Beige Solid	Gum
	Ethyl Acetate	Yellow CS + Off White Solid	CS + Yellow Solid	tBME	N/A	N/A	CS + Beige Solid	Gum
	THF	Yellow CS	Yellow CS	tBME	0.1	White Slurry	CS + Beige Solid	Gum

Table S31. Extended Salt Screening of ACBI1 with p-Toluenesulfonic Acid 0.1 M in four different solvent systems and one Anti-Solvent for the Anti-Solvent Addition (ASA) experiments, and Slow Evaporation (SE) experiments for the Clear Solutions (CS).

Counterion	Solvent	Observation Initial	Observation 3 Day	Anti-Solvent	Volume / mL	Observation	Observation 3 Day	p-XRD Wet
p-Toluenesulfonic acid 0.1 M in THF	2-Propanol	Yellow CS	Yellow CS	tBME	0.2	White Slurry	CS + Beige Solid	Gum
	Acetone	Yellow CS + Yellow Solid	CS + Beige Solid	tBME	N/A	N/A	CS + Beige Solid	Gum
	Ethyl Acetate	Yellow CS + Yellow Solid	CS + Yellow Solid	tBME	N/A	N/A	CS + Beige Solid	Gum
	THF	Yellow CS	Yellow CS	tBME	0.1	White Slurry	CS + Beige Solid	Gum

Table S32. Extended Salt Screening of ACBI1 with Nitric Acid 0.1 M in four different solvent systems and one Anti-Solvent for the Anti-Solvent Addition (ASA) experiments, and Slow Evaporation (SE) experiments for the Clear Solutions (CS).

Counterion	Solvent	Observation Initial	Observation 3 Day	Anti-Solvent	Volume / mL	Observation	Observation 3 Day	p-XRD Wet
Nitric acid 0.1 M in THF	2-Propanol	Yellow CS	CS + Yellow Solid	tBME	N/A	N/A	CS + Beige Solid	Gum
	Acetone	Yellow CS + Yellow Solid	Orange CS	tBME	0.2 Acetone	CS + Beige Solid	CS + Beige Solid	Gum
	Ethyl Acetate	CS + Off White Solid	CS + Yellow Solid	tBME	N/A	N/A	CS + Yellow Solid	Amorphous
	THF	Yellow CS	Yellow CS	tBME	0.1	White Slurry + Beige Solid	CS + Beige Solid	Gum

Table S33. Extended Salt Screening of ACBI1 with Methanesulfonic Acid 0.1 M in four different solvent systems and one Anti-Solvent for the Anti-Solvent Addition (ASA) experiments, and Slow Evaporation (SE) experiments for the Clear Solutions (CS).

Counterion	Solvent	Observation Initial	Observation 3 Day	Anti-Solvent	Volume / mL	Observation	Observation 3 Day	p-XRD Wet
Methanesulfonic acid 0.1 M in THF	2-Propanol	Yellow CS	Yellow CS	tBME	N/A	N/A	Beige Oil	Gum
	Acetone	Yellow CS + Yellow Solid	Orange CS	tBME	0.2 Acetone	CS + Beige Solid	CS + Beige Solid	Gum
	Ethyl Acetate	Yellow CS + Yellow Solid	CS + Yellow Glass	tBME	N/A	N/A	CS + Yellow Solid	Amorphous
	THF	Yellow CS	Yellow CS	tBME	0.1	White Slurry + Beige Solid	CS + Beige Solid	Gum

Table S34. Extended Salt Screening of ACBI1 with 2-Naphthalenesulfonic Acid 0.1 M in four different solvent systems and one Anti-Solvent for the Anti-Solvent Addition (ASA) experiments, and Slow Evaporation (SE) experiments for the Clear Solutions (CS).

Counterion	Solvent	Observation Initial	Observation 3 Day	Anti-Solvent	Volume / mL	Observation	Observation 3 Day	p-XRD Wet
2-Naphthalenesulfonic acid 0.1 M in THF	2-Propanol	Yellow CS + Yellow Solid	CS + Beige Solid	tBME	N/A	N/A	CS + Beige Solid	Gum
	Acetone	Yellow CS + Yellow Solid	CS + Beige Solid	tBME	N/A	N/A	CS + Beige Solid	Gum
	Ethyl Acetate	Yellow CS + White Solid	CS + Beige Solid	tBME	N/A	N/A	CS + Beige Solid	Amorphous
	THF	Yellow CS	Orange CS	tBME	0.1	White Slurry + Beige Solid	CS + Beige Solid	Gum

Table S35. Extended Salt Screening of ACBI1 with Benzenesulfonic Acid 0.1 M in four different solvent systems and one Anti-Solvent for the Anti-Solvent Addition (ASA) experiments, and Slow Evaporation (SE) experiments for the Clear Solutions (CS).

Counterion	Solvent	Observation Initial	Observation 3 Day	Anti-Solvent	Volume / mL	Observation	Observation 3 Day	p-XRD Wet
Benzenesulfonic acid 0.1 M in THF	2-Propanol	Yellow CS	Beige Thin Slurry + Beige Solid	tBME	0.2	White Slurry + Beige Solid	CS + Beige Solid	Gum
	Acetone	Yellow CS + Yellow Solid	CS + Beige Solid	tBME	N/A	N/A	CS + Beige Solid	Gum
	Ethyl Acetate	Yellow CS + White Solid	CS + Yellow Solid	tBME	N/A	N/A	CS + Yellow Solid	Gum
	THF	Yellow CS	Yellow CS	tBME	0.2	White Slurry + Yellow Solid	CS + Beige Solid	Amorphous

Table S36. Extended Salt Screening of ACBI1 with Oxalic Acid 0.1 M in four different solvent systems and one Anti-Solvent for the Anti-Solvent Addition (ASA) experiments, and Slow Evaporation (SE) experiments for the Clear Solutions (CS).

Counterion	Solvent	Observation Initial	Observation 3 Day	Anti-Solvent	Volume / mL	Observation	Observation 3 Day	p-XRD Wet
Oxalic acid 0.1 M in THF	2-Propanol	Yellow Gel	Yellowish Slurry	tBME	N/A	N/A	White Slurry	Amorphous
	Acetone	Yellow CS + Yellow Solid	CS + Yellow Solid	tBME	N/A	N/A	CS + Beige Solid	Oxalate Pattern 1?
	Ethyl Acetate	Yellow CS + Yellow Solid	White Slurry + White Solid	tBME	N/A	N/A	CS + Beige Solid	Amorphous
	THF	Yellow CS	White Slurry + White Solid	tBME	N/A	N/A	Beige Slurry	Oxalate Pattern 1?

Table S37. Extended Salt Screening of ACBI1 with 2,2-Dichloroacetic Acid 0.1 M in four different solvent systems and one Anti-Solvent for the Anti-Solvent Addition (ASA) experiments, and Slow Evaporation (SE) experiments for the Clear Solutions (CS).

Counterion	Solvent	Observation Initial	Observation 3 Day	Anti-Solvent	Volume / mL	Observation	Observation 3 Day	p-XRD Wet
2,2-Dichloroacetic 0.1 M in THF	2-Propanol	Yellow CS	White Slurry + Yellow Solid	tBME	N/A	N/A	CS + Yellow Gum	Gum
	Acetone	Yellow CS	Yellow CS	tBME	0.2	White Slurry + Beige Solid	CS + Brown Gum	Gum
	Ethyl Acetate	Yellow CS + Beige Solid	White Slurry	tBME	N/A	N/A	White Slurry + Beige Solid	Dichloroacetate Pattern 1?
	THF	Yellow CS	Yellow CS	tBME	0.2	White Slurry + Beige Solid	CS + Brown Gum	Gum

Table S38. Extended Salt Screening of ACBI1 with Maleic Acid 0.1 M in four different solvent systems and one Anti-Solvent for the Anti-Solvent Addition (ASA) experiments, and Slow Evaporation (SE) experiments for the Clear Solutions (CS).

Counterion	Solvent	Observation Initial	Observation 3 Day	Anti-Solvent	Volume / mL	Observation	Observation 3 Day	p-XRD Wet
Maleic acid 0.1 M in THF	2-Propanol	Yellow CS + Yellow Solid	Yellow Slurry + Yellow Solid	tBME	N/A	N/A	CS + Beige Gum	Gum
	Acetone	Yellow CS	Brown Gum	tBME	0.1	White Sluryy + Beige Solid	CS + Beige Gum	Gum
	Ethyl Acetate	Yellow CS + Yellow Solid	CS + Yellow Solid	tBME	N/A	N/A	CS + Beige Glass	Amorphous
	THF	Yellow CS	Yellow CS	tBME	0.2	White Sluryy + Beige Solid	CS + Beige Glass	Amorphous

Table S39. Extended Salt Screening of ACBI1 with L-Aspartic Acid 0.1 M in four different solvent systems and one Anti-Solvent for the Anti-Solvent Addition (ASA) experiments, and Slow Evaporation (SE) experiments for the Clear Solutions (CS).

Counterion	Solvent	Observation Initial	Observation 3 Day	Anti-Solvent	Volume / mL	Observation	Observation 3 Day	p-XRD Wet
L-Aspartic acid 0.1 M in Acetone	2-Propanol	Yellow Thin Slurry	White Slurry + Yellow Solid	tBME	N/A	N/A	CS + Beige Gum	Gum
	Acetone	Yellow Thin Slurry	Yellow Gum	tBME	0.3 Acetone	White Slurry	CS + Beige Glass	L-Aspartic Acid
	Ethyl Acetate	Yellow Thin Slurry	Yellow CS + Beige Solid	tBME	N/A	N/A	CS + Beige Gum	Gum
	THF	Yellow Thin Slurry	Yellow Slurry	tBME	N/A	N/A	Beige Slurry	L-Aspartic Acid

Table S40. Extended Salt Screening of ACBI1 with Phosphoric Acid 0.1 M in four different solvent systems and one Anti-Solvent for the Anti-Solvent Addition (ASA) experiments, and Slow Evaporation (SE) experiments for the Clear Solutions (CS).

Counterion	Solvent	Observation Initial	Observation 3 Day	Anti-Solvent	Volume / mL	Observation	Observation 3 Day	p-XRD Wet
Phosphoric acid 0.1 M in THF	2-Propanol	Yellow CS + Yellow Solid	White Slurry + Yellow Solid	tBME	N/A	N/A	CS + Yellow Solid	Amorphous
	Acetone	Yellow CS + Yellow Solid	White Slurry + Yellow Solid	tBME	N/A	N/A	CS + Yellow Solid	Amorphous
	Ethyl Acetate	Yellow CS + Yellow Solid	Yellow Solid	tBME	0.2 EtOAc	Yellow Slurry	CS + Yellow Solid	Amorphous
	THF	Yellow CS + Yellow Solid	Yellow CS	tBME	0.1	White Slurry + Yellow Solid	Off-White Slurry	Amorphous

Table S41. Extended Salt Screening of ACBI1 with (+)-Camphor-10-sulfonic Acid 0.1 M in four different solvent systems and one Anti-Solvent for the Anti-Solvent Addition (ASA) experiments, and Slow Evaporation (SE) experiments for the Clear Solutions (CS).

Counterion	Solvent	Observation Initial	Observation 3 Day	Anti-Solvent	Volume / mL	Observation	Observation 3 Day	p-XRD Wet
(+)-Camphor-10-sulfonic acid 0.1 M in MeOH	2-Propanol	Yellow CS	White Slurry + Yellow Solid	tBME	N/A	N/A	CS + Beige Gum	Gum
	Acetone	Yellow CS	CS + Brown Solid	tBME	N/A	N/A	CS + Beige Gum	Gum
	Ethyl Acetate	Yellow CS + Beige Solid	CS + Brown Solid	tBME	N/A	N/A	CS + Beige Gum	Gum
	THF	Yellow CS	Beige CS	tBME	0.1	White Slurry + Yellow Solid	CS + Beige Gum	Gum

Table S42. Extended Salt Screening of ACBI1 with Glutamic Acid 0.1 M in four different solvent systems and one Anti-Solvent for the Anti-Solvent Addition (ASA) experiments, and Slow Evaporation (SE) experiments for the Clear Solutions (CS).

Counterion	Solvent	Observation Initial	Observation 3 Day	Anti-Solvent	Volume / mL	Observation	Observation 3 Day	p-XRD Wet
Glutamic acid 0.1 M in Acetone	2-Propanol	Yellow Thin Slurry	White Slurry + Yellow Solid	tBME	N/A	N/A	CS + Yellow Gum	Gum
	Acetone	Yellow Thin Slurry	Yellow CS + White Solid	tBME	N/A	N/A	CS + Yellow Solid	Glutamic Acid
	Ethyl Acetate	Yellow Thin Slurry	Yellow CS + Beige Solid	tBME	N/A	N/A	CS + Beige Gum	Gum
	THF	Yellow Thin Slurry	Yellow Slurry	tBME	N/A	N/A	Off-White Slurry	Glutamic Acid

Table S43. Extended Salt Screening of ACBI1 with Saccharin Acid 0.1 M in four different solvent systems and one Anti-Solvent for the Anti-Solvent Addition (ASA) experiments, and Slow Evaporation (SE) experiments for the Clear Solutions (CS).

Counterion	Solvent	Observation Initial	Observation 3 Day	Anti-Solvent	Volume / mL	Observation	Observation 3 Day	p-XRD Wet
Saccharin 0.1 M	2-Propanol	Yellow CS	White Solid	tBME	0.3 IPA	CS	CS + White Solid	Amorphous
	Acetone	Yellow CS	Brown Solid	tBME	0.3 Acetone	Yellow CS	Yellow CS	Gum
	Ethyl Acetate	Yellow CS + Beige Solid	Beige Solid	tBME	0.3 EtOAc	CS	CS + Yellow Solid	Amorphous
	THF	Yellow CS	Yellow CS	tBME	0.1	CS + Beige Solid	CS + Beige Gum	Gum

Table S44. Extended Salt Screening of ACBI1 with Malonic Acid 0.1 M in four different solvent systems and one Anti-Solvent for the Anti-Solvent Addition (ASA) experiments, and Slow Evaporation (SE) experiments for the Clear Solutions (CS).

Counterion	Solvent	Observation Initial	Observation 3 Day	Anti-Solvent	Volume / mL	Observation	Observation 3 Day	p-XRD Wet
Malonic acid 0.1 M in EtOH	2-Propanol	Yellow Thin Slurry	Yellow Solid	tBME	N/A	N/A	CS + Yellow Gum	Gum
	Acetone	Yellow CS	Yellow CS	tBME	0.1	White Slurry + Yellow Solid	CS + Beige Gum	Gum
	Ethyl Acetate	Yellow CS + Yellow Solid	CS+ Yellow Solid	tBME	N/A	N/A	CS + Yellow Glass	Amorphous
	THF	Yellow CS	Yellow CS	tBME	0.2	White Slurry + Beige Solid	CS + Yellow Glass	Amorphous

Table S45. Extended Salt Screening of ACBI1 with Gentisic Acid 0.1 M in four different solvent systems and one Anti-Solvent for the Anti-Solvent Addition (ASA) experiments, and Slow Evaporation (SE) experiments for the Clear Solutions (CS).

Counterion	Solvent	Observation Initial	Observation 3 Day	Anti-Solvent	Volume / mL	Observation	Observation 3 Day	p-XRD Wet
Gentisic acid 0.1 M	2-Propanol	Yellow Thin Slurry	Yellow Solid	tBME	N/A	N/A	CS + Yellow Gum	Gum
	Acetone	Yellow CS	Yellow CS	tBME	0.1	Yellow Slurry	Yellow CS + Brown Gum	Gum
	Ethyl Acetate	Yellow CS + Yellow Solid	CS + Yellow Solid	tBME	N/A	N/A	CS + Yellow Glass	Amorphous?
	THF	Yellow CS	Yellow CS	tBME	0.2	Yellow Slurry	Yellow CS + Brown Gum	Gum

Table S46. Extended Salt Screening of ACBI1 with (+)-L-Tartaric Acid 0.1 M in four different solvent systems and one Anti-Solvent for the Anti-Solvent Addition (ASA) experiments, and Slow Evaporation (SE) experiments for the Clear Solutions (CS).

Counterion	Solvent	Observation Initial	Observation 3 Day	Anti-Solvent	Volume / mL	Observation	Observation 3 Day	p-XRD Wet
(+)-L-Tartaric acid 0.1 M in MeOH	2-Propanol	White Slurry + Yellow Solid	Yellow Solid	tBME	N/A	N/A	CS + Yellow Solid	Amorphous
	Acetone	Yellow CS + Yellow Solid	CS + Yellow Solid	tBME	N/A	N/A	CS + Yellow Solid	Amorphous
	Ethyl Acetate	Yellow CS + Yellow Solid	CS + Yellow Solid	tBME	N/A	N/A	CS + Yellow Solid	(+)-L-Tartaric acid
	THF	Yellow CS	White Slurry + Yellow Solid	tBME	N/A	N/A	Yellow CS	Gum

Table S47. Extended Salt Screening of ACBI1 with Fumaric Acid 0.1 M in four different solvent systems and one Anti-Solvent for the Anti-Solvent Addition (ASA) experiments, and Slow Evaporation (SE) experiments for the Clear Solutions (CS).

Counterion	Solvent	Observation Initial	Observation 3 Day	Anti-Solvent	Volume / mL	Observation	Observation 3 Day	p-XRD Wet
Fumaric acid 0.1 M in EtOH	2-Propanol	Yellow CS	Beige Solid	tBME	N/A	N/A	CS + Brown Gum	Gum
	Acetone	Yellow CS	CS + Brown Solid	tBME	N/A	N/A	CS + Brown Gum	Gum
	Ethyl Acetate	Yellow CS + Yellow Solid	Yellow CS + Yellow Solid	tBME	N/A	N/A	CS + Yellow Glass	Amorphous
	THF	Yellow CS	Yellow CS	tBME	0.2	White Slurry	CS + Yellow Glass	Gum

Solvent Drop Grinding (SDG):

Approximately 20.00 mg were weighed in the suitable SDG plastic tubes, appropriate amount of the selected three acids, and three stainless steel metallic beads were inserted. Minimal amount of the appropriate solvent, 0.020 mL, was added and the tube was closed with the appropriate cap. Observations were recorded (Table S48 - S50) and the following program was initiated and repeated twice:
RPM: 4000
Intervals: 80 s milling, 10 s pause
Repetition: 10 times
Total time: 30 min
Post SDG, observations were recorded, and the material was transferred to the appropriate sample holders for p-XRD analysis.

Table S48. Solvent Drop Grinding of ACBI1 with 1,5-Naphthalenedisulfonic Acid 0.1 M in six different solvent systems.

**Counterion**	**Mass (mg) / Volume (µL)**	**Mass (mg)**	**Solvent**	**Volume (mL)**	**Stainless Steel Grinding Beads**	**Observation Initial**	**Observation 30 Minutes**	**p-XRD Wet**
1,5-Naphthalenedisulfonic acid 0.1 M in MeOH	7.20	20.09	2-Propanol	0.02	3	Yellow Solid	Yellow Solid	Amorphous
	6.90	19.98	Acetone	0.02	3	Yellow Solid	Yellow Solid	Napadisylate Pattern 2
	6.81	19.8	Ethyl Acetate	0.02	3	White Solid	White Solid	Amorphous
	6.93	19.85	THF	0.02	3	Off-White Solid	Off-White Solid	Amorphous
	7.07	20.02	tBME	0.02	3	Off-White Solid	Off-White Solid	Amorphous
	6.83	20.19	Ethyl Acetate : tBME 1 : 1	0.02	3	White Solid	White Solid	Amorphous

Table S49. Solvent Drop Grinding of ACBI1 with 1,2-Ethanedisulfonic Acid 0.1 M in four different solvent systems.

**Counterion**	**Mass (mg) / Volume (µL)**	**Mass (mg)**	**Solvent**	**Volume (mL)**	**Stainless Steel Grinding Beads**	**Observation Initial**	**Observation 30 Minutes**	**p-XRD Wet**
1,2-Ethanedisulfonic Acid in THF	4.89	19.97	2-Propanol	0.02	3	Yellow Solid	Yellow Solid	Amorphous
	4.62	20	Acetone	0.02	3	White Solid	White Solid	Predominantly Amorphous
	4.59	19.83	Ethyl Acetate	0.02	3	White Solid	White Solid	Amorphous
	4.92	19.78	THF	0.02	3	Yellow Solid	Yellow Solid	Amorphous

Table S50. Solvent Drop Grinding of ACBI1 with Cyclamic Acid 0.1 M in four different solvent systems.

**Counterion**	**Mass (mg) / Volume (µL)**	**Mass (mg)**	**Solvent**	**Volume (mL)**	**Stainless Steel Grinding Beads**	**Observation Initial**	**Observation 30 Minutes**	**p-XRD Wet**
Cyclamic Acid in THF	4.37	20.12	2-Propanol	0.02	3	Yellow Solid	Yellow Solid	Cyclamate Pattern 3
	4.62	20.13	Acetone	0.02	3	Yellow Solid	Yellow Solid	Cyclamate Pattern 3
	4.21	19.93	Ethyl Acetate	0.02	3	Yellow Solid	Yellow Solid	Predominantly Amorphous
	4.49	20.25	THF	0.02	3	White Solid	White Solid	Amorphous

Vapour Diffusion

The investigated compound was dissolved into the appropriate solvents in HPLC vials, and low partial pressure anti-solvents were selected for vapour diffusion experiments. Two acids, in two API : acid ratios, were added into the dissolved API and the vial was inserted into a larger vial that contained the anti-solvent. The larger vial was tightly closed and were placed into a cupboard for a month. Observations were recorded after a month.

Table S51. Vapour Diffusion of ACBI1 with Cyclamic Acid and 1,5-Naphthalenedisulfonic Acid in DMSO with different ratios and solvent systems.

ACBI1	Solvent	Acid	Anti-solvent	Ratio API : Acid	Observations 1 month
1	Dimethylsulfoxide	Cyclamic acid	Methanol	1 : 1	Clear Solution
				1 : 2	Clear Solution
			Diethyl Ether	1 : 1	Brown Gel + Clear Solution
				1 : 2	Brown Gel + Crystals + Clear Solution
			Acetone	1 : 1	Crystals + Pale Yellow Solution
				1 : 2	Crystals + Pale Yellow Solution
			Ethyl Acetate	1 : 1	Brown Gel + Crystals + Clear Solution
				1 : 2	Brown Gel + Crystals + Clear Solution
			Acetonitrile	1 : 1	Brown Swell + Clear Solution
				1 : 2	Brown Swell + Clear Solution
			Tetrahydrofuran	1 : 1	Crystals + Pale Yellow Solution
				1 : 2	Crystals + Pale Yellow Solution
		1,5-Naphthalenedisulfonic acid	Methanol	1 : 1	Yellow Gel + Clear Solution
				1 : 2	Yellow Gel + Clear Solution
			Diethyl Ether	1 : 1	Yellow Gel + Clear Solution
				1 : 2	Yellow Gel + Clear Solution
			Acetone	1 : 1	Yellow Gel + Clear Solution
				1 : 2	Yellow Gel + Clear Solution
			Ethyl Acetate	1 : 1	Yellow Gel + Clear Solution
				1 : 2	Yellow Gel + Clear Solution
			Acetonitrile	1 : 1	Yellow Gel + Clear Solution
				1 : 2	Yellow Gel + Clear Solution
			Tetrahydrofuran	1 : 1	Yellow Gel + Clear Solution
				1 : 2	Yellow Gel + Clear Solution

Table S52. Vapour Diffusion of ACBI1 with Cyclamic Acid and 1,5-Naphthalenedisulfonic Acid in NMP with different ratios and solvent systems.

ACBI1	Solvent	Acid	Anti-solvent	Ratio API : Acid	Observations 1 month
2	N-Methylpyrrolidone	Cyclamic acid	Methanol	1 : 1	Yellow Clear Solution
				1 : 2	Yellow Clear Solution
			Diethyl Ether	1 : 1	Yellow Gel + Crystals + Clear Solution
				1 : 2	Yellow Gel + Crystals + Clear Solution
			Acetone	1 : 1	Yellow Swell + Crystals + Clear Solution
				1 : 2	Crystals + Clear Solution
			Ethyl Acetate	1 : 1	Yellow Gel + Crystals + White Slurry
				1 : 2	Yellow Gel + Crystals + White Slurry
			Acetonitrile	1 : 1	Yellow Swell + Crystals + Clear Solution
				1 : 2	Crystals + Clear Solution
			Tetrahydrofuran	1 : 1	Crystals + Clear Solution
				1 : 2	Crystals + Clear Solution
		1,5-Naphthalenedisulfonic acid	Methanol	1 : 1	Yellow Bubbles + Clear Solution
				1 : 2	Yellow Bubbles + Clear Solution
			Diethyl Ether	1 : 1	Yellow Gel + Crystals + Clear Solution
				1 : 2	Yellow Gel + Crystals + Clear Solution
			Acetone	1 : 1	Yellow Gel + Crystals + Clear Solution
				1 : 2	Yellow Gel + Crystals + Clear Solution
			Ethyl Acetate	1 : 1	Yellow Gel + Crystals + Clear Solution
				1 : 2	Yellow Gel + Crystals + Clear Solution
			Acetonitrile	1 : 1	Yellow Gel + Crystals + Clear Solution
				1 : 2	Yellow Gel + Clear Solution
			Tetrahydrofuran	1 : 1	Yellow Gel + Crystals + Clear Solution
				1 : 2	Yellow Gel + Crystals + Clear Solution

Experimental procedures BI00201335:

Approximate solvent solubility (ASS):

Approximately 20.00 mg were weighed in HPLC vials, and a magnetic stirrer bar was inserted. Water bath temperature was set at 40 °C, and the initial 25 µL of the appropriate solvent was added into the vial. Stirring was initiated and the slurries were brought up to the desired set temperature. Observations were recorded and the next addition was performed according to the Table S53. The samples were stirred at 40 °C overnight and observations were recorded after 24 h.

Table S53. Approximate Solvent Solubility (ASS) for BI201335, with "x" indicating that a slurry was observed, while "o" indicated a clear solution (CS). Observations were recorded in two different timepoints.

**BI201335 - ASS -**	**Solvent**	**Mass (mg)**	**Volume of Solvent added (µL)**										**Initial Observations**	**Observations 24 h ambient**
			**50**	**16.7**	**13.3**	**20**	**33.3**	**66.7**	**66.7**	**133.3**	**400**	**1200**		
1	1-Propanol	19.58	x	x	o								Pale Yellow CS	Pale Yellow CS
2	2-Methyl THF	20.33	x	x	x	o							Pale Yellow CS	Pale Yellow CS
3	2-Propanol	19.85	x	x	o								Pale Yellow CS	Pale Yellow CS
4	Acetone	20.44	x	x	o								Pale Yellow CS	Pale Yellow CS
5	Acetonitrile	19.58	x	x	x	x	x	x	o				Pale Yellow CS	Solid + Pale Yellow CS
6	Dimethylsulfoxide	20.13	x	o									Pale Yellow CS	Pale Yellow CS
7	Ethanol	20.17	x	x	x	o							Pale Yellow CS	Solid + Pale Yellow CS
8	Ethyl Acetate	20.42	x	x	x	x	x	x	x	o			Pale Yellow CS	Pale Yellow CS
9	Heptane	20.43	x	x	x	x	x	x	x	x	x	x	Pale Yellow Slurry	Pale Yellow Slurry
10	Water	19.55	x	x	x	x	x	x	x	x	x	x	Pale Yellow Slurry Hydrophobic	Pale Yellow Slurry Hydrophobic
Concentration (mg / mL)			400	300	250	200	150	100	75	50	25	10		

Thermal Cycling and Slow Solvent Evaporation (SE):

Approximately 20.00 mg were weighed in HPLC vials, and a magnetic stirrer bar was inserted. Water bath temperature was set at 40 °C, and 200 μL of the appropriate solvent was added into the vial. Stirring was initiated and the slurries were brought up to the desired set temperature. Observations were recorded and the following program was initiated and repeated under stirring and kept for 72 h:

- 2 h hold at 40 °C
- Ramp down to RT with rate of 0.1 °C / min
- 2 h hold at RT
- Ramp up to 40 °C with rate of 0.1 °C / min
- Repeat

Post the 72-h thermal cycling, observations were recorded and the HPLC cap was pierced once, and the samples were stored into a cupboard for slow evaporation. After 7 days the samples afforded solids, which were analysed by p-XRD and indicated only amorphous material, except ethanol which resulted in diffraction pattern 1, known as polymorphic Form I.

Table S54. Thermal Cycling (TC) and Slow Solvent Evaporation (SE) for BI201335. Observations were recorded in different timepoints and p-XRD analysis was performed after the completion of the experiments.

BI201335 - TC - SE -	Solvent	Observations	Observations 7 days ambient	p-XRD
				Wet
1	1-Propanol	Pale Yellow CS	Yellowish Solid / Glass	Amorphous
2	2-Methyl THF	Pale Yellow CS	Yellowish Solid / Glass	Amorphous
3	2-Propanol	Pale Yellow CS	Yellowish Solid / Glass	Amorphous
4	Acetone	Pale Yellow CS	Yellowish Solid / Glass	Amorphous
5	Acetonitrile	Solid + Pale Yellow CS	Yellowish Solid / Glass	Amorphous
6	Dimethylsulfoxide	Pale Yellow CS	Pale Yellow CS	Amorphous
7	Ethanol	Solid + Pale Yellow CS	Yellowish Solid	Form I
8	Ethyl Acetate	Pale Yellow CS	Yellowish Solid / Glass	Amorphous
9	Heptane	Pale Yellow Slurry	Yellowish Solid / Glass	Amorphous
10	Water	Pale Yellow Slurry Hydrophobic	Pale Yellow Slurry Hydrophobic	Amorphous

Computational SI

MM-ML exploration Approach:

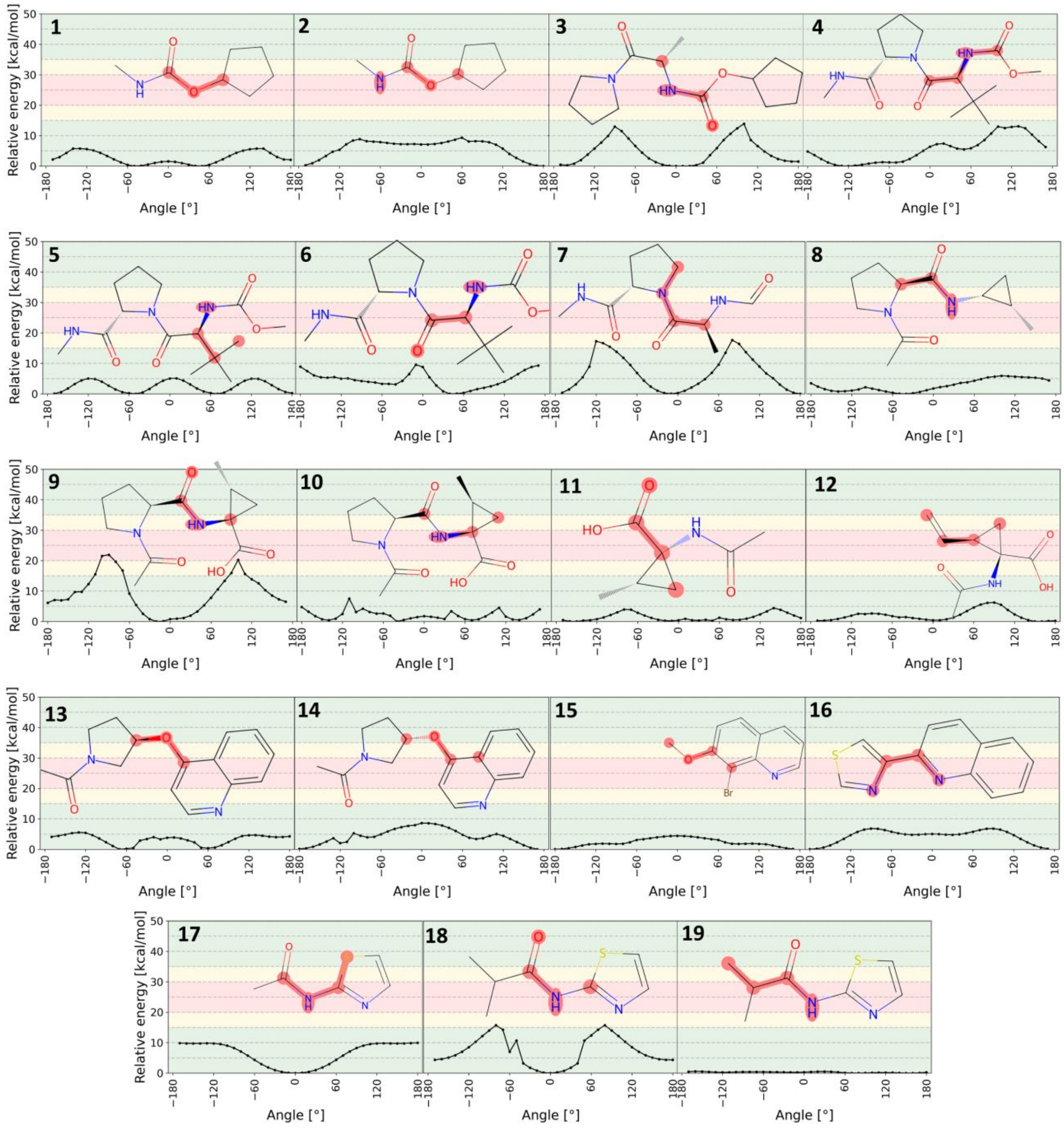

Figure S1. All ML-MM Torsion Profiles on small fragments of BI201335 – the numbers indicate the rotational bond from Figure 5

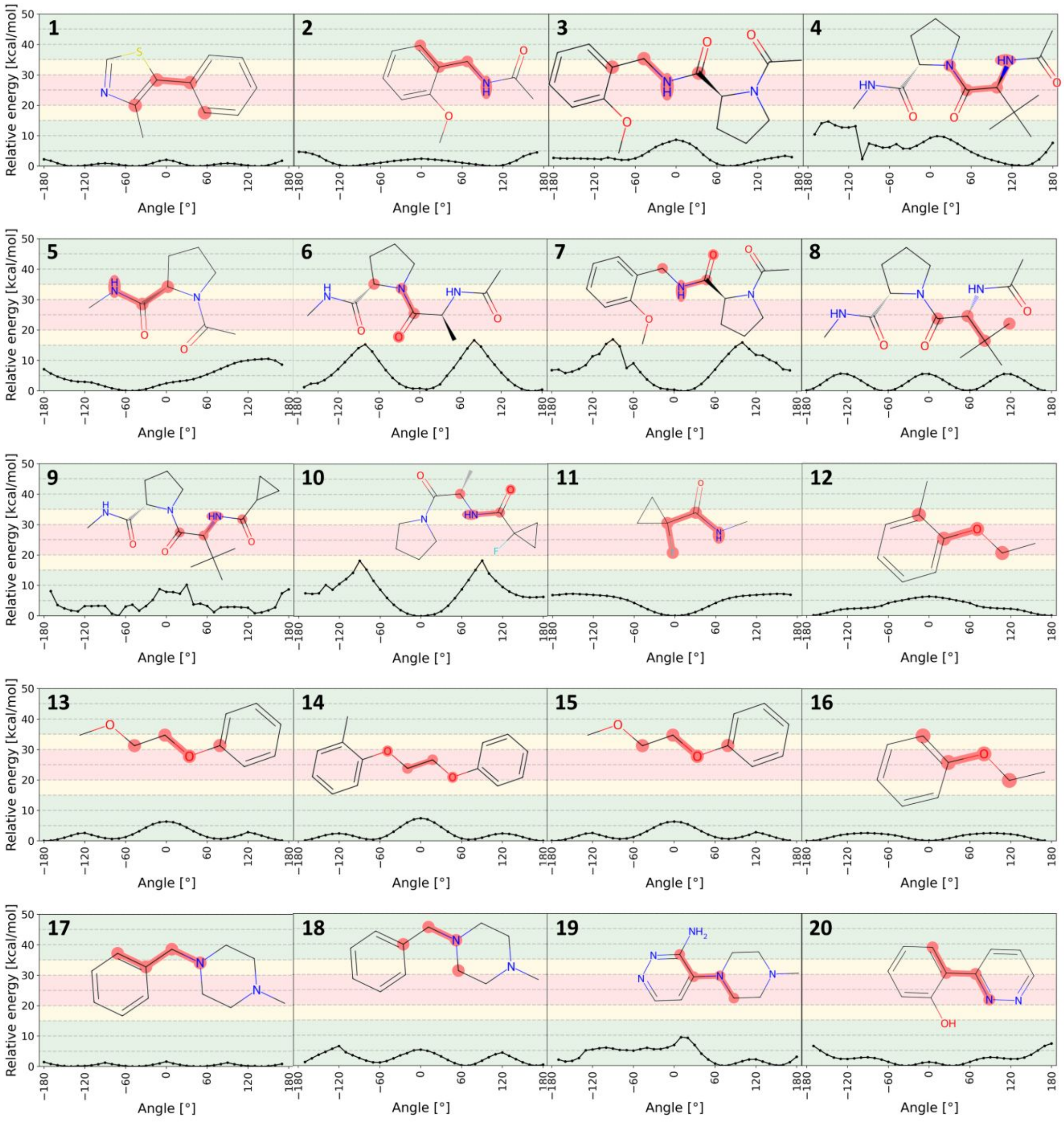

Figure S2. All ML-MM Torsion Profiles on small fragments of ACBI1 – the numbers indicate the rotational bond from Figure 5

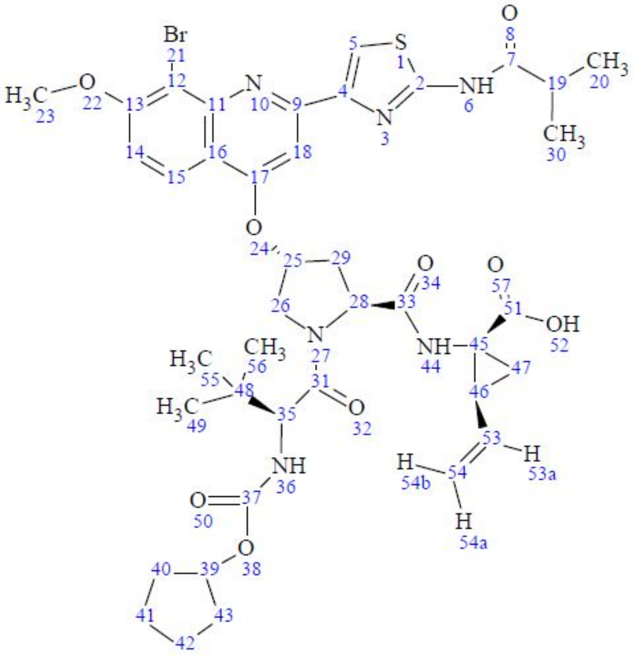

Scheme S1. Assignment scheme numbering for BI201335

Table S55. Atom assignment for BI201335

Atom No.	^{13}C Shift [ppm]	^{1}H Shift [ppm]		Atom No	^{13}C Shift [ppm]	^{1}H Shift [ppm]
2	158.3			31	170.2	
4	154.1			33	171.3	
5	113.8	8.04		35	59.3	4.1
6		12.35		36		6.50; 6.99
7	175.6			37	156.3	
9	149.2			39	76.3	4.62; 4.95
11	146.7			40	32.0	1.26-1.82
12	108.7			41	23.1	1.26-1.82
13	157.3			42	23.1	1.26-1.82
14	112.4	7.35; 7.49		43	32.0	1.26-1.82
15	122.5	8.01; 8.16		44		8.56; 8.75
16	116.3			45	39.0	
17	160.1			46	32.9	2.03
18	97.9	7.47		47	22.1	1.28+1.56
19	33.7	2.83		48	34.0	
20	19.0	1.16		49	26.3	0.89; 0.97
23	56.7	4.01		51	171.6	
25	76.8	5.42		53	134.9	5.73; 5.82
26	53.2	3.96+4.38		54	116.7	5.06+5.20
28	58.2	4.46; 4.76		55	26.3	0.89; 0.97
29	34.1	2.27+2.56		56	26.3	0.89; 0.97
30	19.0	1.16				

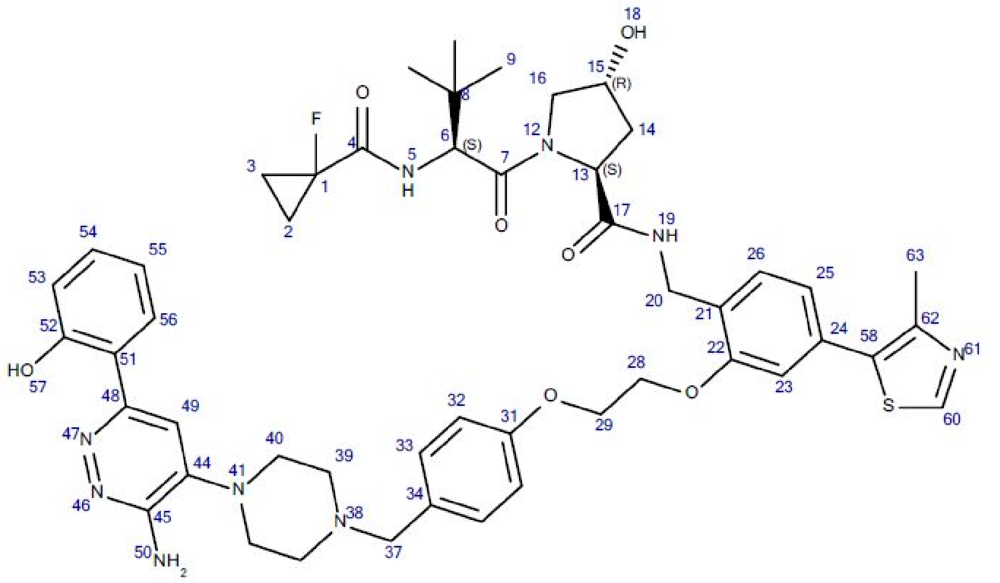

Scheme S2. Assignment scheme numbering for ACBI1

Table S56. Atom assignment for ACBI1

Atom No.	^{13}C Shift [ppm]	^{1}H Shift [ppm]		Atom No.	^{13}C Shift [ppm]	^{1}H Shift [ppm]
1	78.1			31	157.5	
2, 3	12.6, 12.9	1.21+1.35		32	114.3	6.98
4	168.0			33	130.1	7.26
5		7.27		34	130.1	
6	56.5	4.46; 4.60		37	61.3	3.50
7	168.9			39	51.8	2.59
8	36.0			40	48.5	3.11
9	26.1	0.95		44	140.4	
13	58.8	4.53; 4.69		45	154.6	
14	37.8	1.91+2.08		48	153.1	
15	68.8	4.34		49	110.3	7.50
16	56.6	3.59+3.64		50		6.20
17	171.7			51	117.8	
18		5.00; 5.14		52	158.5	
19		8.45; 8.54		53	117.3	6.89
20	37.2	4.21+4.30		54	130.0	7.23
21	127.2			55	118.4	6.88
22	155.6			56	126.0	7.90
23	112.2	7.10		57		14.2
24	130.9			58	131.2	
25	121.2	6.99		60	151.4	8.98
26	127.8	7.42		62	147.9	
28	67.1	4.41		63	15.9	2.47
29	66.4	4.36				

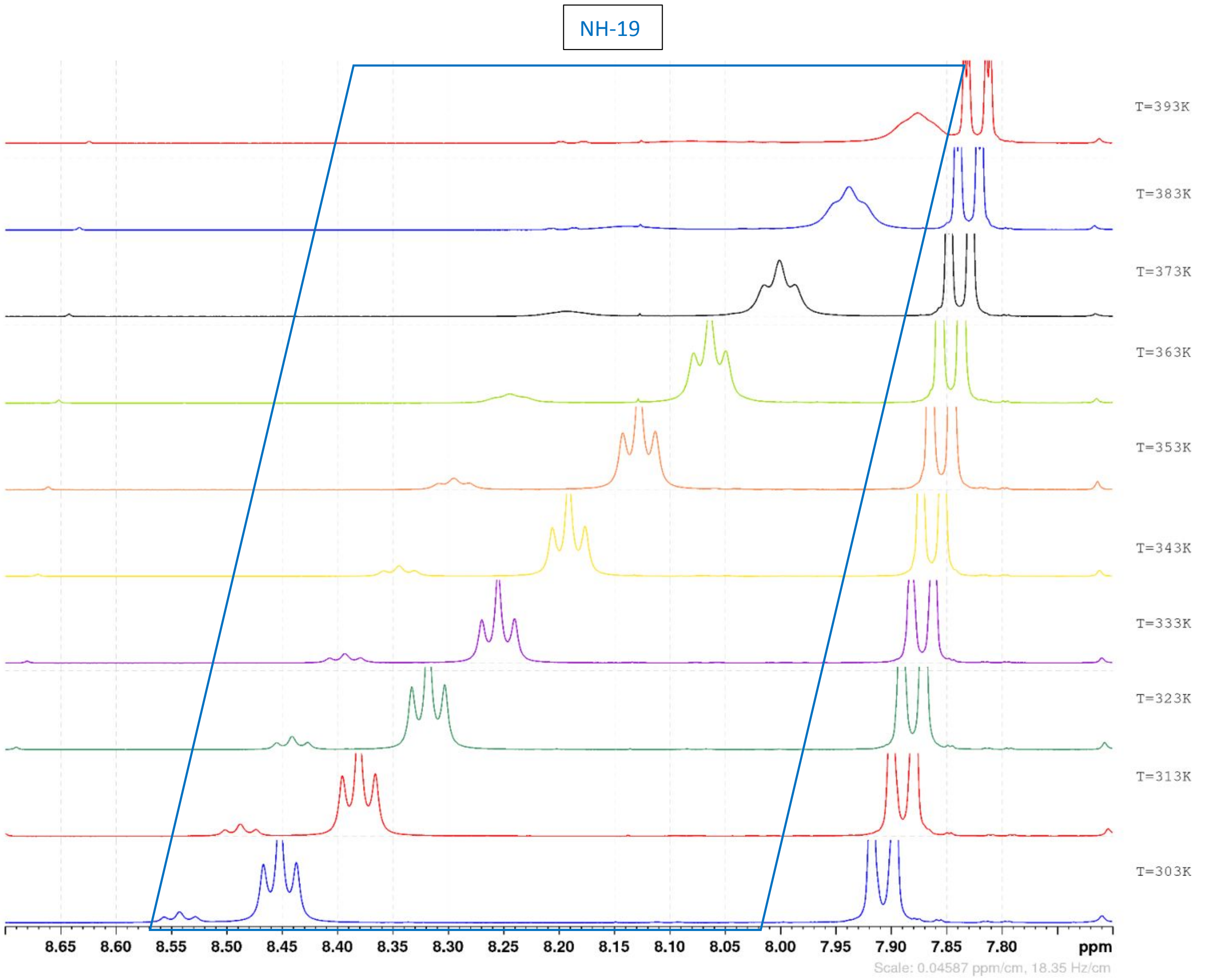

Figure S3. One dimensional proton NMR experiments of the ACBI1 performed varying the measurement temperature in 10 degrees steps, starting from 303K (bottom spectrum) and finishing at 393K (top spectrum). The double set of signals at 8.45 ppm and 8.55 ppm vanish towards the baseline at 393K as their coalescence temperature gets closer. Note that signal assignment is available in Table S56 in the supplementary information

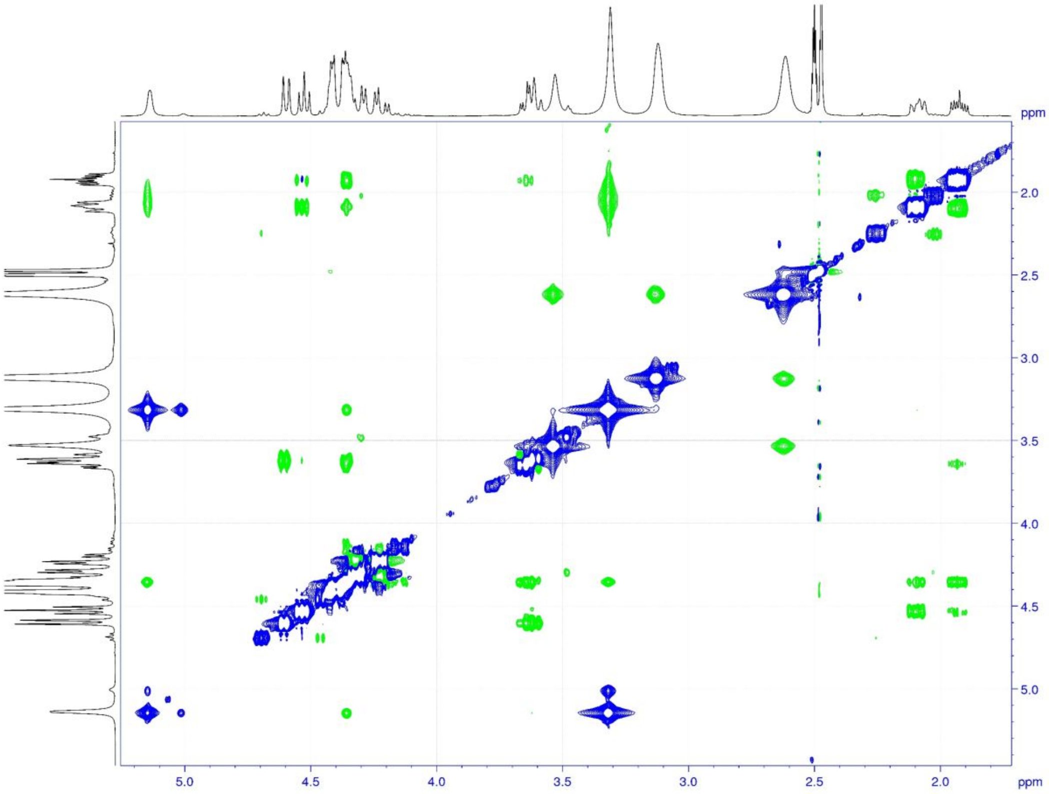

Figure S4. Two-dimensional proton ROESY NMR experiments of the ACBI1 performed at 303K, showing no exchange is taking place between the double set of signals at 2.07 ppm and 2.24 ppm as well as in the range between 3.45 ppm and 3.74 ppm, and between 4.18 ppm and 4.76 ppm at this temperature. The exchange cross peaks are coloured in blue as the diagonal signals, while the NOE proximities over space have an anti-phase with respect to the diagonal and are coloured in green. Exchange cross peaks are observed merely for the hydroxyl group. Due to the complexity of the molecule a third set of signals in the ROESY spectrum cannot be excluded. Note that signal assignment is available in Table S56 in the supplementary information

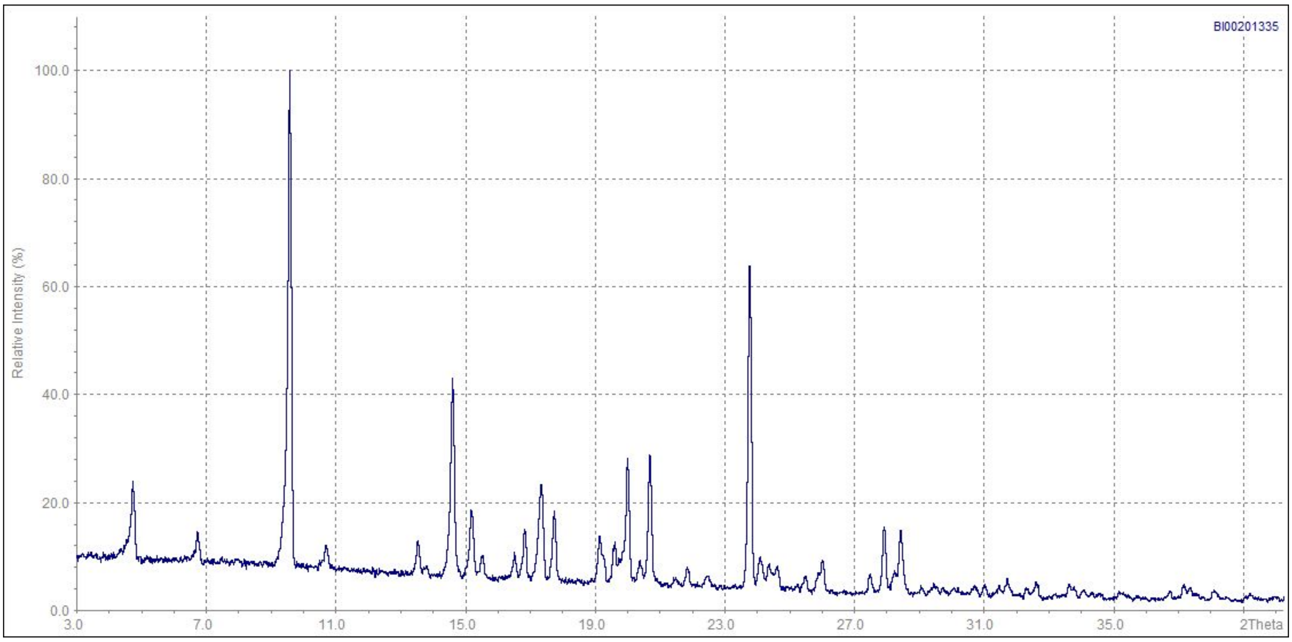

Figure S5. Powder XRD diffractogram of BI201335 Form I.

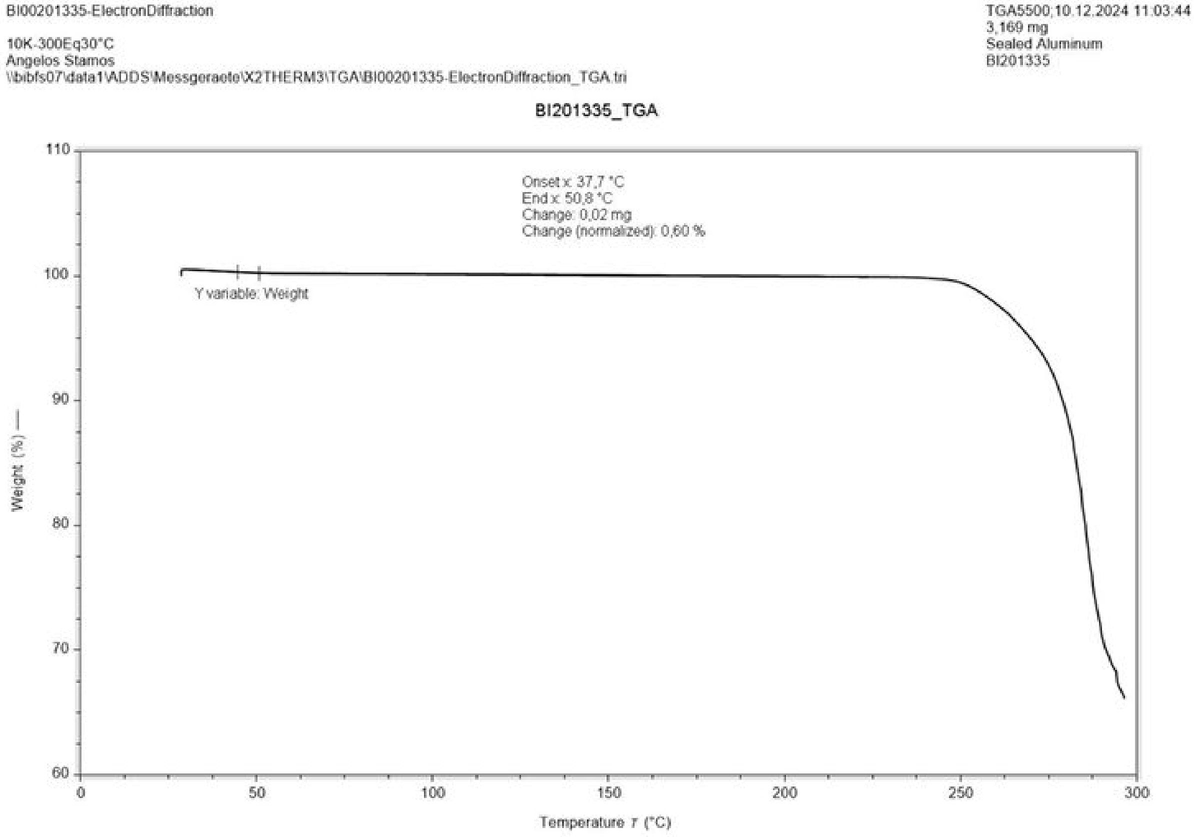

Step transition

Onset x	End x	Change	Change (normalized)	Y variable
37,7 °C	50,8 °C	0,02 mg	0,60 %	Weight

Figure S6. TGA thermogram of BI201335, indicating a minimal amount of weight loss of 0.60% w/w with an onset of 37.7 °C.

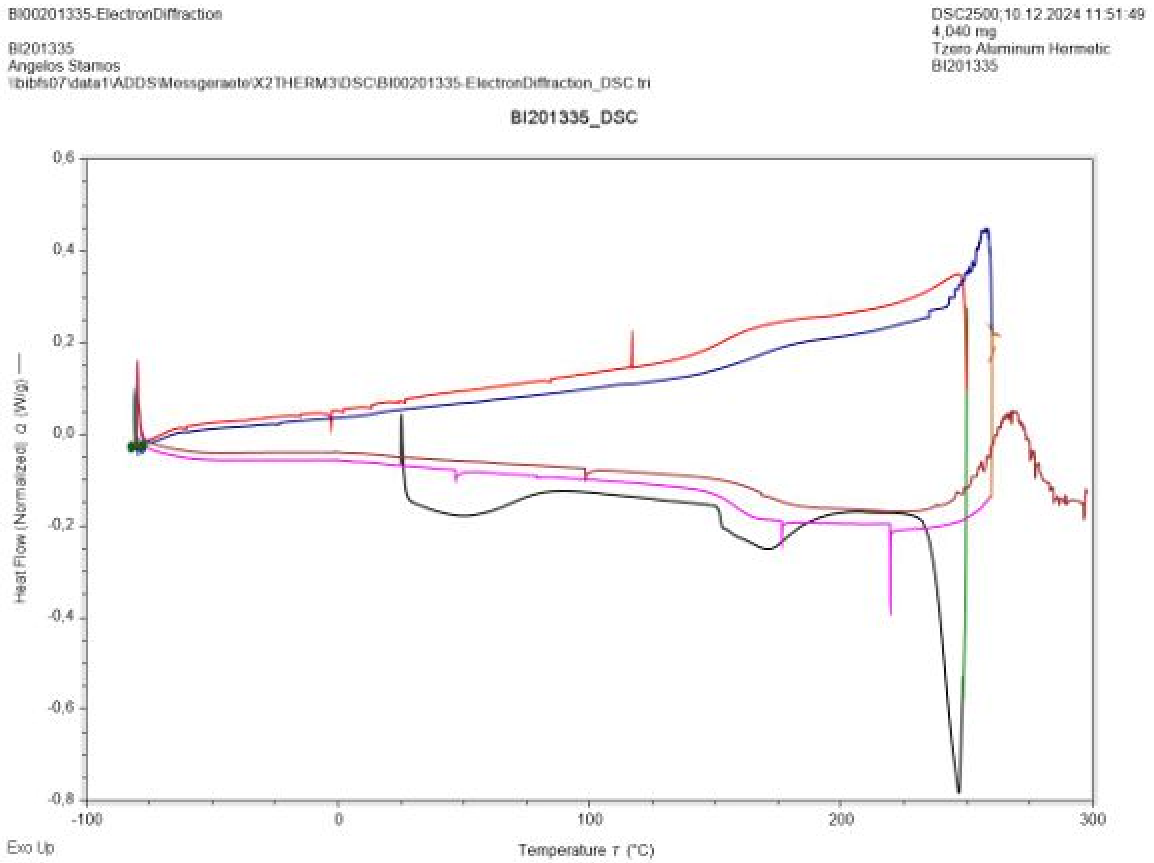

Test	Custom
Test Name	BI201335
Segment 1	Equilibrate 25,0 °C
Segment 2	Ramp 10,00 °C/min to 250,0 °C
Segment 3	Isothermal 5,0 min
Segment 4	Ramp 10,00 °C/min to -80,0 °C
Segment 5	Isothermal 5,0 min
Segment 6	Ramp 10,00 °C/min to 260,0 °C
Segment 7	Isothermal 5,0 min
Segment 8	Ramp 10,00 °C/min to -80,0 °C
Segment 9	Isothermal 5,0 min
Segment 10	Ramp 10,00 °C/min to 300,0 °C
Use Standby Temperature	Yes
Discard pan in waste bin at end of test	No
End of Test Use Standby Temperature	Yes

***Figure S7.** DSC thermogram of BI201335, heating from RT up to 250 °C (black), cooling to -80 °C (red), heating to 260 °C (magenta), cooling to -80 °C (blue) and finally heating to 300 °C (brown), with a 10 °C/min rate. Endothermic events were observed at the initial heating, with an onset of 27.4 °C and 151.1 °C, which possibly indicate solvent evaporation and at 250 °C a melting point was observed. At the subsequent runs, only glass transition points can be observed and no recrystallisation was observed.*

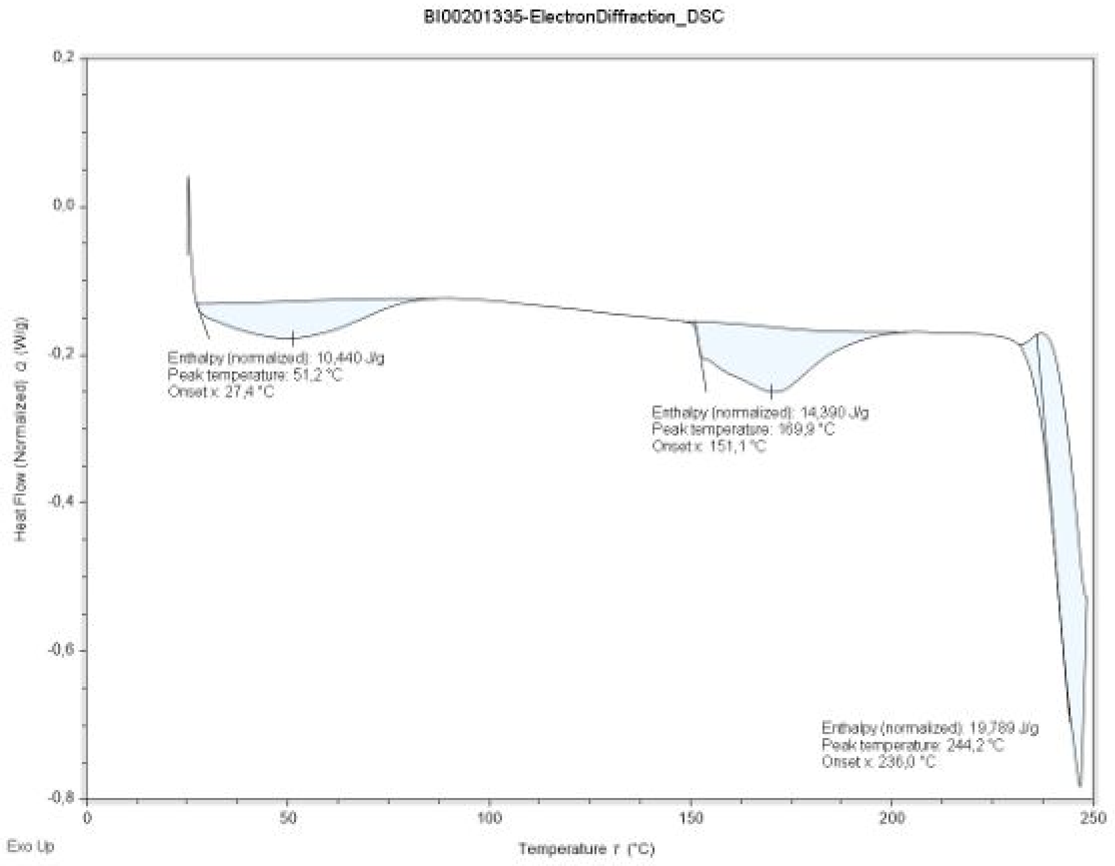

Peak Integration (enthalpy)

Enthalpy (normalized)	Peak temperature	Onset x
10,440 J/g	51,2 °C	27,4 °C
14,390 J/g	169,9 °C	151,1 °C
19,789 J/g	244,2 °C	236,0 °C

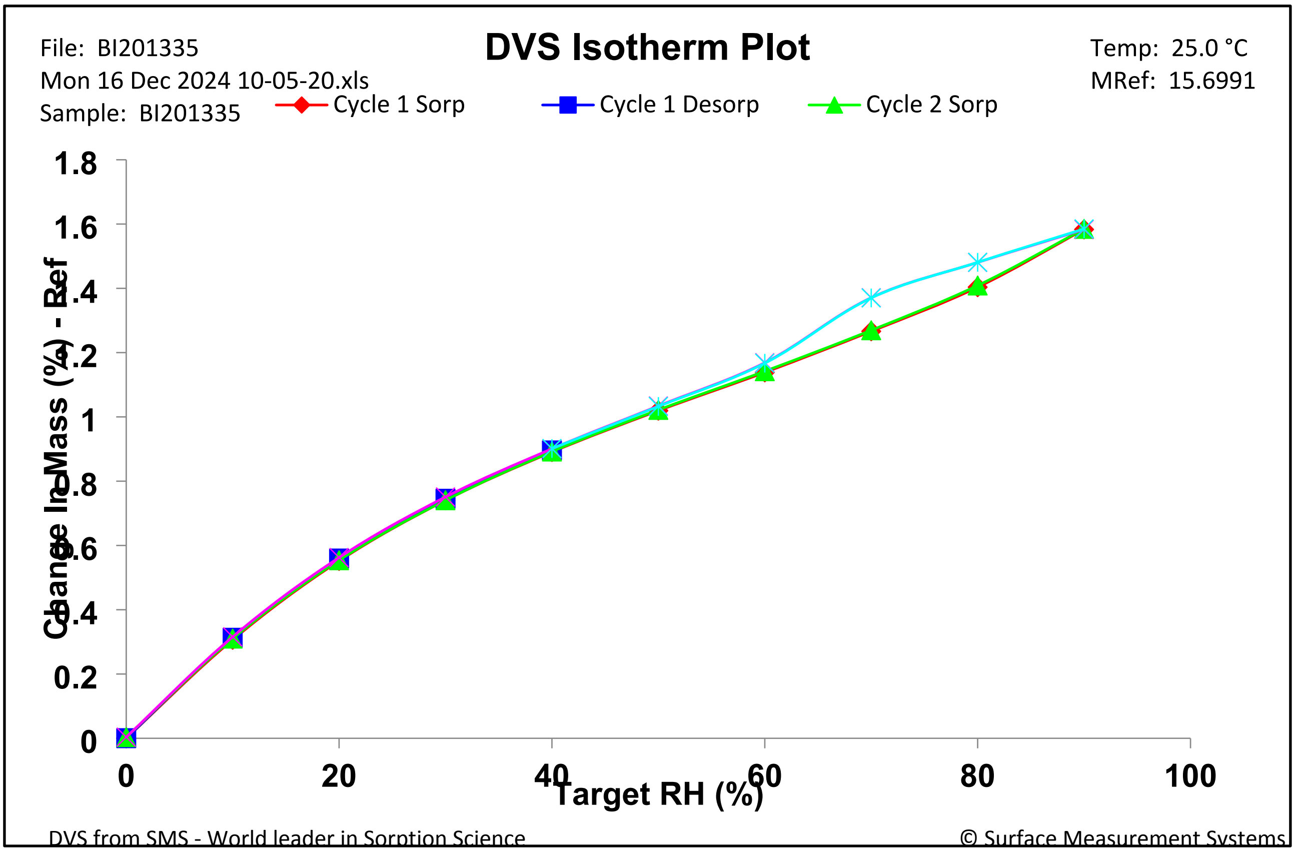

Figure S8. DVS Isotherm plot of BI201335, indicating 1.4 % water adsorption at 80 % RH, which according to the European Pharmacopeia the material is slightly hygroscopic.

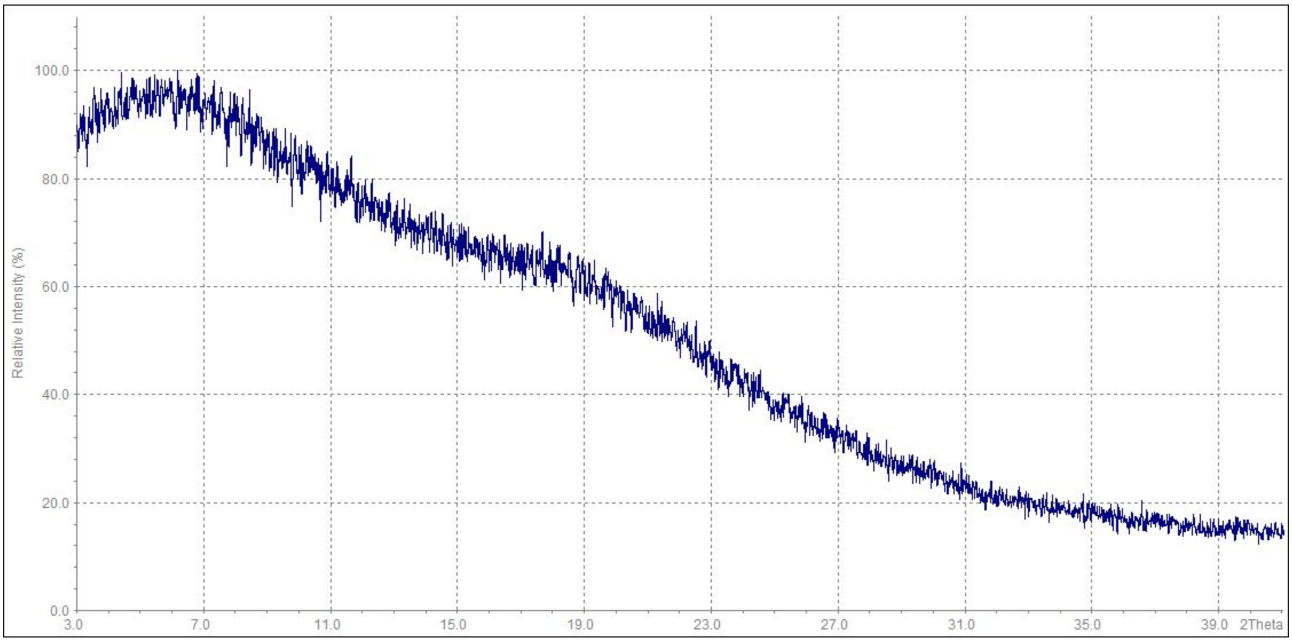

Figure S9. Powder XRD diffractogram of ACBI1 input material.

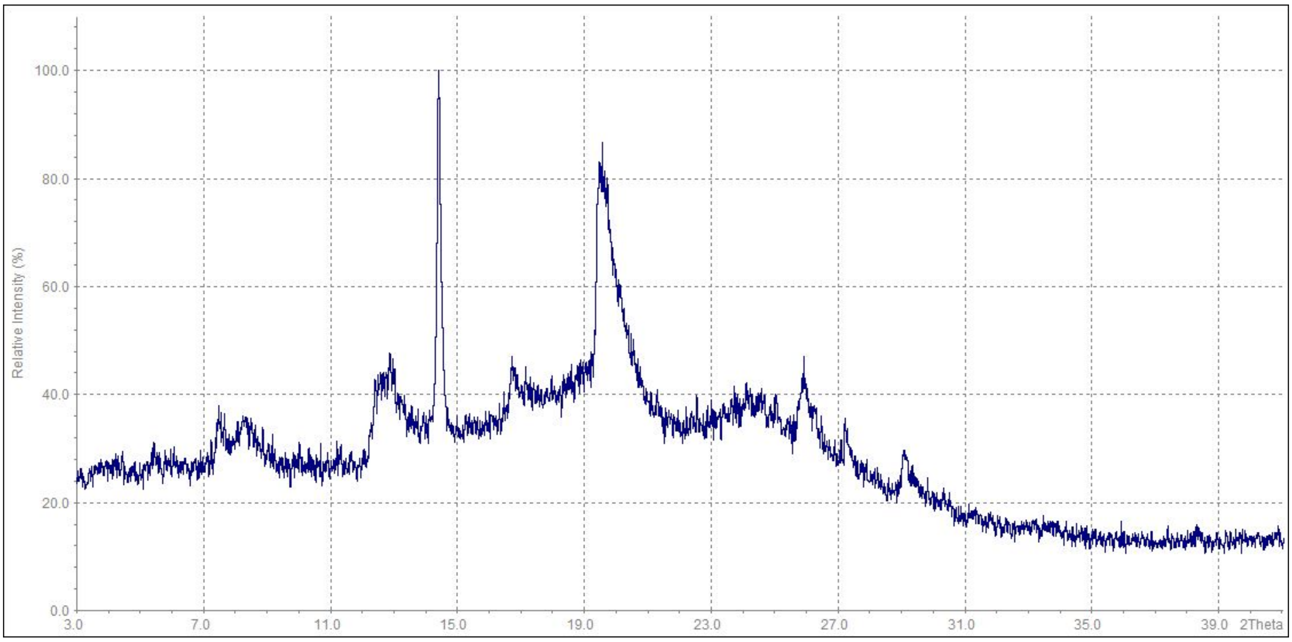

Figure S10. Powder XRD diffractogram of ACBI1 with 1,5-Naphthalenedisulfonic acid in EtOAc at primary salt screening.

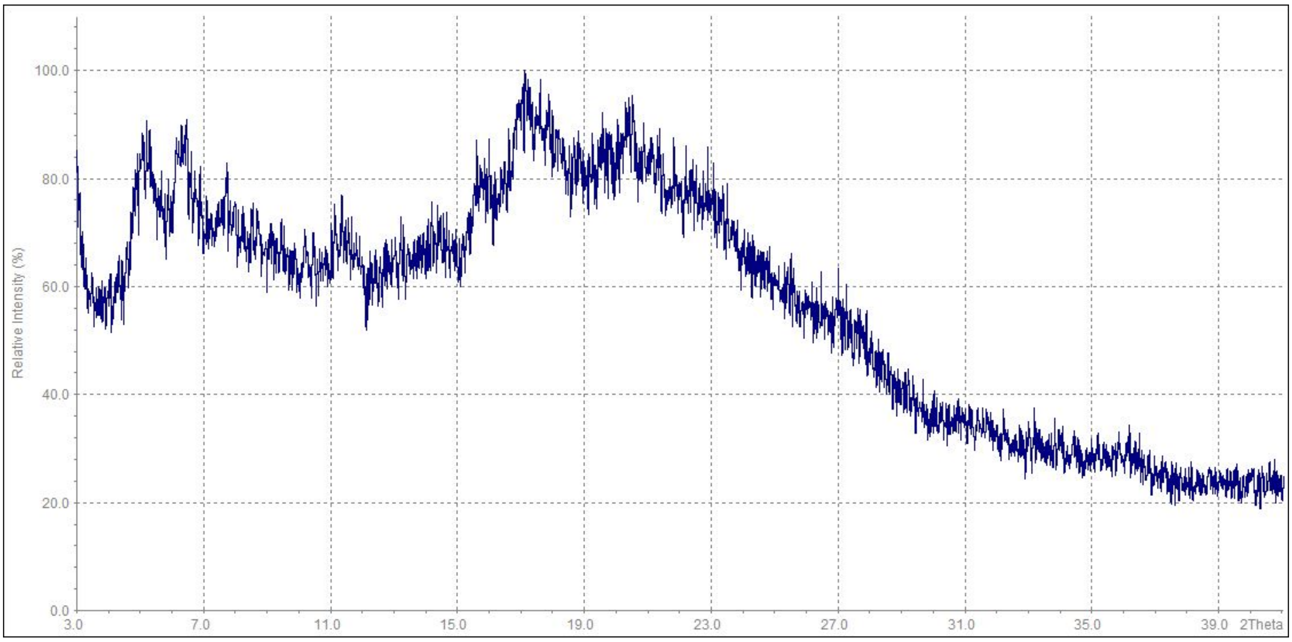

Figure S11. Powder XRD diffractogram of ACBI1 with Oxalic acid in Acetone at primary salt screening.

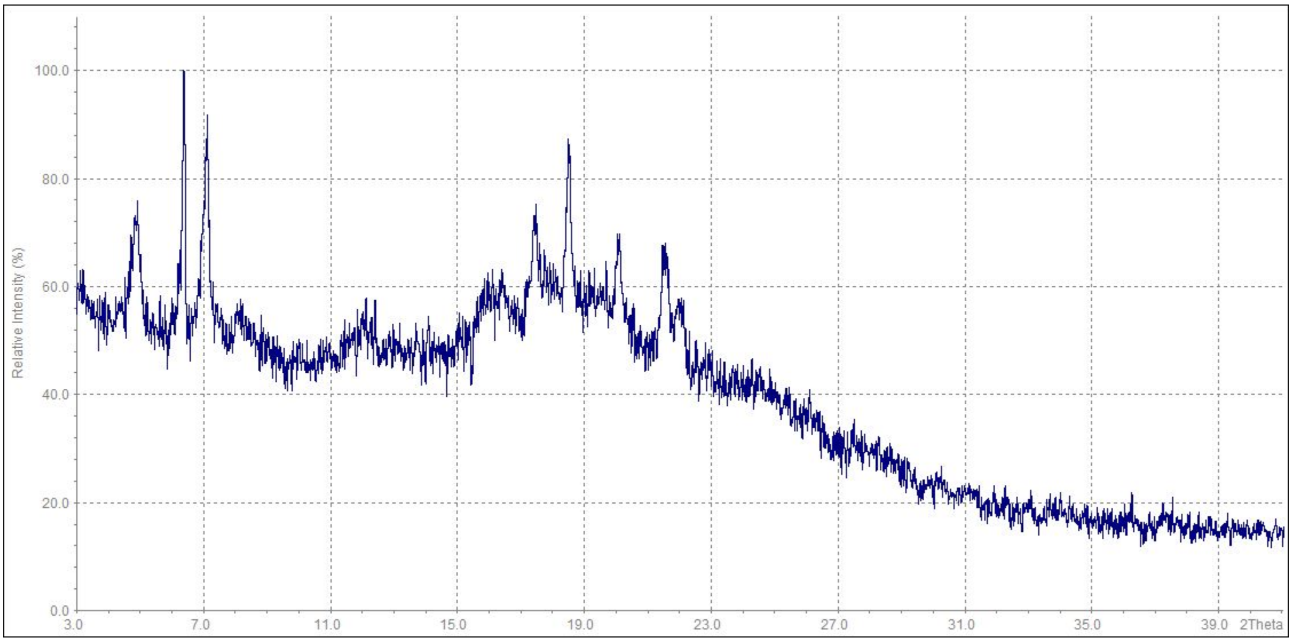

Figure S12. Powder XRD diffractogram of ACBI1 with Cyclamic acid in EtOAc at extended salt screening.

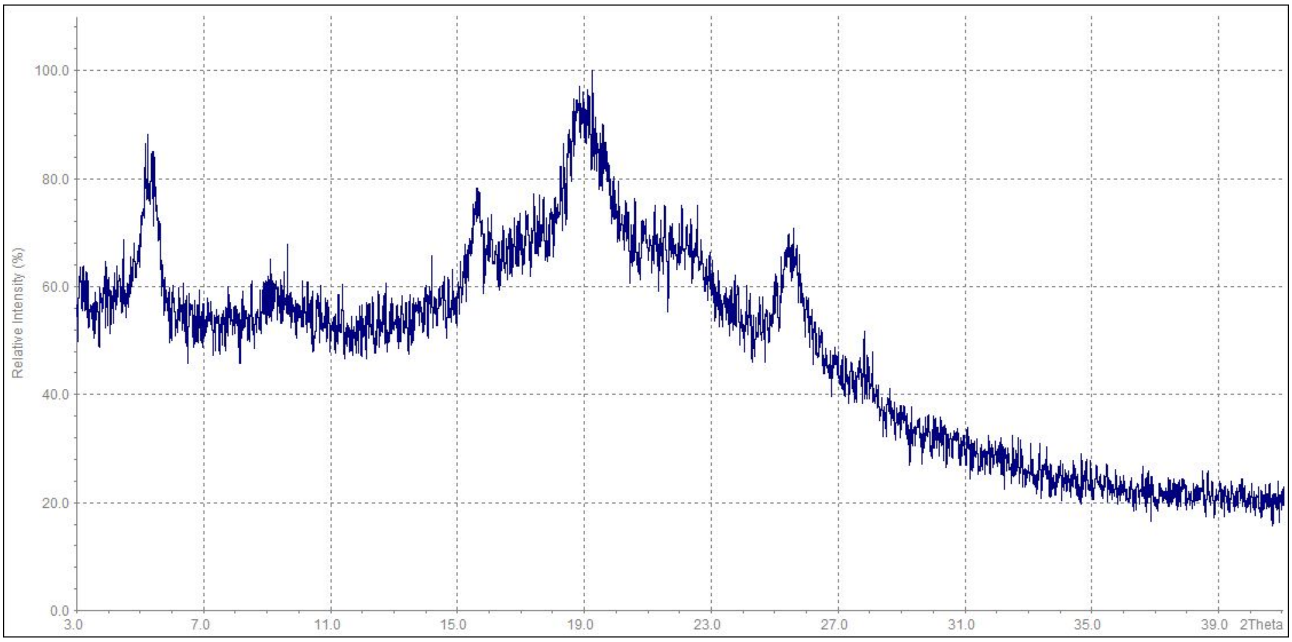

Figure S13. Powder XRD diffractogram of ACBI1 with 1,2-Ethanedisulfonic acid in EtOAc at extended salt screening.

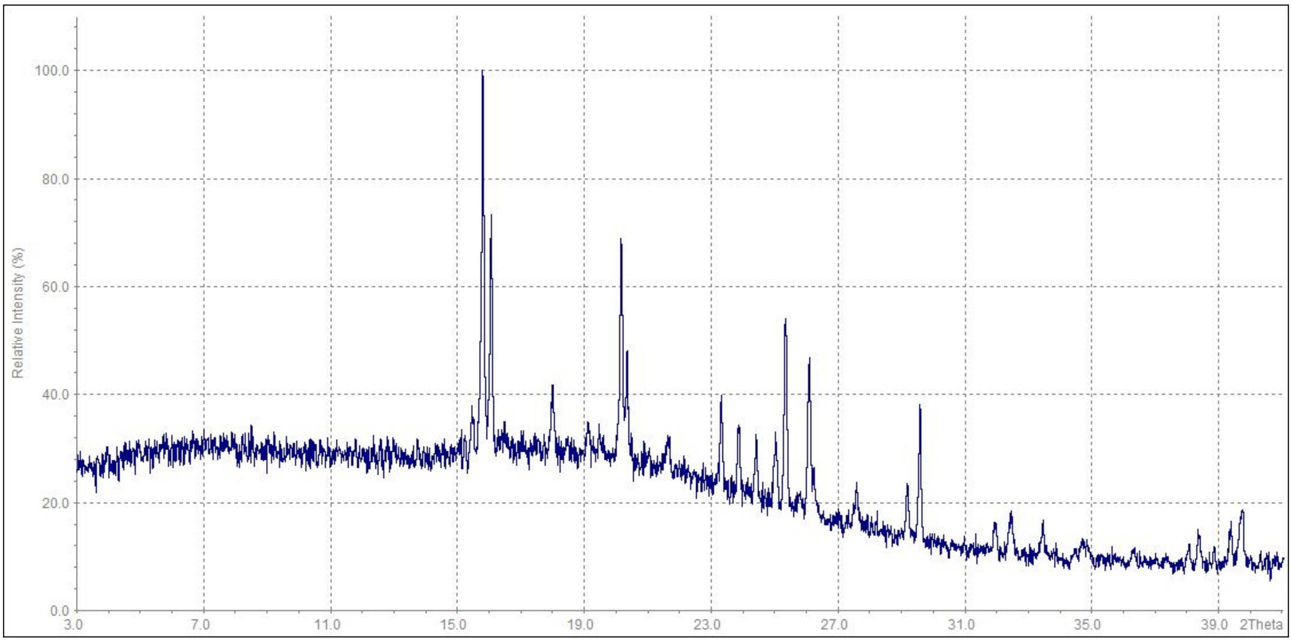

Figure S14. Powder XRD diffractogram of ACBI1 with 1,5-Naphthalenedisulfonic acid in EtOAc at extended salt screening.

1. Tyler, A. R. *et al.* Encapsulated Nanodroplet Crystallization of Organic-Soluble Small Molecules. *Chem* **6**, 1755–1765 (2020).
2. Metherall, J. P., Corner, P. A., McCabe, J. F., Hall, M. J. & Probert, M. R. High-throughput nanoscale crystallization of dihydropyridine active pharmaceutical ingredients. *Acta Crystallogr. Sect. B Struct. Sci. Cryst. Eng. Mater.* **80**, 4–12 (2024).
